# Integrative genomic approaches to unravel genomic regions and candidate genes associated with flag leaf photosynthesis at the reproductive stage in rice

**DOI:** 10.3389/fpls.2026.1752716

**Published:** 2026-04-23

**Authors:** Awais Riaz, Anuj Kumar, Yheni Dwiningsih, Alexander Silva Cordoba, Ainong Shi, Caio Canella Vieira, Andy Pereira, Julie Thomas

**Affiliations:** 1 Department of Crop, Soil, and Environmental Sciences, University of Arkansas, Fayetteville, AR, United States; 2 Department of Horticulture, University of Arkansas, Fayetteville, AR, United States

**Keywords:** chlorophyll fluorescence, genomic estimated breeding values, GWAS, haplotype, photosynthesis, rice

## Abstract

Photosynthesis is a complex polygenic trait that directly influences crop yield and remains a challenging physiological trait to dissect genetically. This study is novel in its large-scale evaluation of photosynthetic performance across 181 diverse rice accessions, representing six subpopulations, under controlled conditions. By integrating photosynthetic measurements from the flag leaf at the booting stage with genome-wide association studies (GWASs) using 3.7 million high-quality single nucleotide polymorphism (SNP) markers, candidate genes were identified for photosynthesis. A total of 18 putative quantitative trait nucleotides (QTNs) [logarithmic odds (LOD) ≥ 8.0] were identified across the MLMM, FarmCPU, and BLINK models and are dispersed on all chromosomes except 5 and 10, including two QTNs (1-2050328 and 12-11380740) that were commonly identified in two models. Following QTN identification, gene mining analysis revealed 1,091 structural and regulatory genes in flanking regions. Subsequently, fine mapping using the gene haplotype analysis suggested 43 genes, including signaling/regulatory genes (receptor like kinases, F-box, RING, and auxin-responsive genes), gene regulators (histones, NAC/NAM, and PPR), membrane trafficking/transport (Exo70 and ADP-ribosylation factor), stress and defense components (heat shock protein, thionin, and MAC/perforin), and 19 uncharacterized proteins, of which seven genes were further selected as candidate gene using ortholog and regulatory analyses. The candidate genes may associate with photosynthesis, including carotenoid isomerase and various kinases, along with genes involved in stomatal regulation (OsABA4), sugar transport (sugar transporter 14 and UDP-glucose transporter), and leaf development (auxin-responsive genes), collectively contributing to efficient photosynthate production and assimilate translocation. Furthermore, the integration of genomic prediction analyses across GWAS, ridge regression best linear unbiased prediction (RRB), and bootstrap trees (BTS) models identified 44 common SNPs corresponding to these candidate regions, thereby enhancing the accuracy of genomic breeding value estimation across the population. To further explore the phenotypic response of photosynthesis based on subpopulation, five diverse rice accessions were selected for a detailed study of light response (A/Q), carbon dioxide response (A/Ci), and non-photochemical quenching (NPQ), enabling the assessment of photosynthetic variation across diverse genetic backgrounds. This study identified significant putative genomic regions, candidate genes, and a set of SNP markers associated with high photosynthesis in rice accessions, providing valuable resources for plant breeding and genomics-assisted breeding to enhance rice yield.

## Introduction

Rice (*Oryza sativa* L.) is an important cereal crop that plays a key role in ensuring food security. It has been providing 35% to 75% of daily calorie intake, especially in Asian, Latin American, and African countries (Mohanty, 2013). Rice consumption is expected to rise significantly due to the exponentially growing population. It has been predicted that the global population will exceed 9.0 billion by 2050, hence necessitating an increase in the agricultural production of at least 1% per year to meet future food demands. A rice ideotype with a high harvest index is one of the approaches to increase yield potential by allocating a greater proportion of photosynthates to grain production. The prominent example of this approach is the green revolution, which introduced semidwarf cultivars in the mid-20th century. These cultivars were characterized by shorter stems, lodging resistance, and a significantly high harvest index due to a substantial amount of photosynthates assimilated into grain (Hedden, 2003; Long et al., 2006; Zhu et al., 2010; Acevedo-Siaca et al., 2020; Gaur et al., 2020). The flag leaf has the highest photosynthetic capacity at the heading stage and serves as the primary source of assimilate production during the grain filling phase. Consequently, measuring flag leaf photosynthesis (Pn) at the booting stage can identify genetic variation linked to yield potential, as the flag leaf plays a key role in photosynthate production and translocation that support grain filling in rice.

Rice, a C_3_ plant, fixes CO_2_ into a three-carbon compound during photosynthesis, a process strongly influenced by light intensity (I) and CO_2_ concentration (Gutteridge, 2018). Studies using light response (A/Q) and CO_2_ response (A/Ci) curves (Baly, 1935; Smith Emil, 1936; Ogren and Evans, 1993; Bassman and Zwier, 1991; Posada et al., 2009; Farquhar et al., 1980; Ye et al., 2013; Von Caemmerer, 2000; Ralph and Gademann, 2005; Ye et al., 2021) have shown how these factors regulate light and dark reactions. Non-photochemical quenching (NPQ) helps the leaf dissipate excess light, thereby maintaining the integrity of the photosynthetic apparatus under high light or stress conditions and ensuring sustained photosynthetic efficiency (Nicol et al., 2019). However, C_3_ crops like rice are less efficient than C_4_ crops due to higher photorespiration and lower photosynthesis efficiency (Wang et al., 2012). Therefore, improving photosynthetic efficiency in rice through targeted research is crucial for enhancing carbon assimilation and yield.

Identifying quantitative trait loci (QTLs)/genes controlling Pn is essential for accumulating the favorable alleles in cultivars, leading to trait improvement (Gu et al., 2012). To date, only a few genes have been functionally characterized for photosynthesis improvement, including GREEN FOR Photosynthesis (GPS), Higher yield rice (HYR), and Carbon Assimilation Rate 8 (CAR8) (Takai et al., 2013; Ambavaram et al., 2014; Adachi et al., 2017) in rice. Multiple studies have identified QTLs and genes associated with photosynthetic traits using mapping populations derived from bi-parental or multi-parental crosses (Takai et al., 2010; Adachi et al., 2017 & 2019; Yadavalli et al., 2022). These family-based QTL mapping approaches cause limited allelic diversity, leading to low resolution and inaccurate gene identification. A possible solution to these limitations is to employ diverse genetic panels together with modern high-throughput approaches to uncover the genetic determinants of polygenic traits (Korte and Farlow, 2013; Riaz et al., 2023).

Many studies on genetic variation in Pn under saturated light intensity and ambient CO_2_ concentration have been reported among rice genotypes (Adachi et al., 2011 & Adachi et al., 2017; Sasaki and Ishii, 1992; Hubbart et al., 2007; Takai et al., 2013). Leveraging this diversity through modern genomic approaches, particularly genome-wide association studies (GWASs), enables the high-resolution detection of causative loci and the identification of nucleotide, structural, and haplotype variations underlying this complex trait (Zhou et al., 2020; Wang et al., 2020). Although GWAS has been successfully applied to dissect a range of morpho-physiological traits in rice (Li et al., 2023; Kumar et al., 2021; Yi et al., 2023), only a few studies have identified or characterized genomic regions and candidate genes associated with Pn and photosynthetic-related traits (Honda et al., 2024; Hamdani et al., 2019; Wei et al., 2022), highlighting the need for further studies using diverse germplasm and integrative genomic analyses.

Alongside these advances, genomic prediction (GP) offers a complementary approach to GWAS by enabling the estimation of genomic breeding values from molecular marker data. Conventional breeding remains limited by labor-intensive phenotypic selection and inaccuracies arising from the polygenic control of complex traits. In contrast, GP uses molecular markers and training/testing populations to provide a cost-effective and efficient approach that enhances selection accuracy, breeding efficiency, and genetic gain across generations. However, GP applications in rice remain limited (Spindel et al., 2015; Nguyen et al., 2023; Bartholomé et al., 2023).

In this study, we studied the genetic basis of photosynthetic rate (Pn) in a diverse rice panel using the Li-COR 6400 XT under controlled Green house (GH) conditions along with high-density SNP genotyping. By integrating GWAS, genomic prediction models, and haplotype profiling, we aimed to identify candidate genes, superior haplotypes, and potential rice accessions for breeding programs. These results provide novel insights into the improvement of photosynthetic efficiency, a key target for enhancing rice productivity.

## Material and method

### Plant material and growth conditions

The URMC [United States Department of Agriculture (USDA) rice mini-core collection] rice diversity panel, representative of six subpopulations, was obtained from the USDA ARS Dale Bumper National Rice Research Center, Stuttgart, AR, USA (Agrama et al., 2010; **Supplementary Table 1a**). These accessions were purified and multiplied before the experiment using the single seed descent method (Kumar et al., 2021). The experiment was conducted using a randomized complete block design with three replications of each accession at the Harry R. Rosen Alternative Pest Control Center at the University of Arkansas, Fayetteville, AR, USA. Seedlings were grown in small plastic pots and transplanted 14 days post-germination, with one seedling per pot measuring 25 × 25 cm, filled with a 3:1 mixture of SunGro professional potting mix (Sun Gro Horticulture Distribution, Agawam, MA, USA) and field clay soil. These pots were placed in trays of size 15.24 cm by 30.48 cm, and each pot was considered a single replication. The GH conditions, including temperature (30 °C/22 °C ± 1 °C day/night), relative humidity (65%–70%), and light intensity [800–1,000 μmol photosynthetically active radiation (PAR) m^–2^ s^–1^] with a 14-h photoperiod, were maintained until the maturity of the crop. The established protocol of Kumar et al. (2021) was followed for irrigation, plant fertigation, and pest control at various plant stages under GH conditions.

### Gas exchange measurements

At the reproductive stage, the flag leaf of each accession was tagged for Pn measurement using Li-COR 6400 XT (LI-COR, Nebraska, USA) on sunny days from 09:00 to 14:00 h to minimize circadian effect on photosynthesis (Adachi et al., 2011). All plants were well light-adapted prior to measurement. The flag leaf was placed in the IRGA chamber, and Pn values were recorded once it stabilized, along with water contents and leaf CO_2_ level, which mostly required 3–5 minutes. Two additional Pn values were recorded from the same leaf at an interval of 1.5 minutes to improve the reliability for GWAS analysis. The IRGA chamber was set to control conditions with leaf temperature of 25 °C ± 0.1 °C, [CO_2_] ref of 410 μmol mol^−1^, a chamber flow rate of 400 μmol s^−1^, and relative humidity ranging from 60% to 70%. Photosynthetic photon flux density (PPFD) was maintained at ~1,500 photons μmol m^−2^ s^−1^, and gas leakage was monitored to ensure that it remained <0.5 CO_2_ μmol mol^−1^. The vapor pressure deficit (VPD) in air recorded during measurements ranged from 0.45 to 1.92 kPa. To ensure consistency across the panel, measurements were randomized with respect to accession and position in the greenhouse. The instrument calibration was checked periodically, and Pn values of selected high and low Pn accessions were repeatedly measured from the panel. The phenotypic data, including Pn, stomata conductance (mol H_2_O m^−2^ s^−1^), transpiration rate (mol m^−2^ s^−1^), vapor pressure deficit of air (VpdA) (kPa), were processed by removing the outlier using the box plot method, and normality was assessed using the Shapiro–Wilk test to ensure comparability across accessions (**Supplementary Tables 1b, c**). Pairwise associations among all measured traits were measured using Pearson’s correlation coefficient using the R package “ggstatsplot”. Although measurements were taken between 09:00 and 14:00 to reduce circadian effects, time and day were not included in the statistical model and may have contributed to variation.

### Photosynthetic response curves

Five rice accessions were selected from the diversity panel for exploratory photosynthetic measurements, including A/Q, A/Ci, and NPQ. These genotypes were selected after the initial screening of the diversity panel for Pn, diverse background, and contrasting grain quality response under heat stress (data not shown). Nipponbare was selected as a low-photosynthesis, stress-susceptible Tmp japonica accession, whereas Zhe733, Cypress, N22, 310045, and GSOR310080 represented medium-to-high photosynthetic performance with contrasting subpopulation and stress-response backgrounds (N22 is a drought- and heat-tolerant aus accession; Cypress, 310045, and GSOR310080 are heat-tolerant Trp japonica accessions; Zhe733 is a high-yielding indica accession). These accessions were grown with six replications in a randomized complete block design, and measurements were performed on a defined leaf area by selecting three flag leaves per accession. This subset captures both the phenotypic extremes for photosynthesis and key adaptive backgrounds relevant for breeding, as well as contrasting grain quality responses to heat stress, thereby enabling deeper investigation of the underlying physiological mechanisms.

#### Carbon dioxide (A/Ci curve) analysis

For the A/Ci curve, plant adaptation, temperature, PPFD, and relative humidity were kept consistent as mentioned above for Pn reading across a series of [CO_2_] ref levels: 400, 300, 250, 100, 50, 400, 400, 400, 500, 700, 900, 1,200, and 1,400 μmol mol^−1^. Flag leaf was held in the leaf chamber for 60 to 90 s at [CO_2_] ref from 400 to 50 μmol mol^−1^ and for 180–360 s from 400 to 1,400 μmol mol^−1^. These measurements were used for the A/Ci curve, with Ci on the x-axis and A (Pn) on the y-axis. The carboxylation rate (V_cmax_), electron transport rate (ETR), triphosphate utilization (TPU), CO_2_ compensation point (Γ), and maximum net photosynthetic rate (A_max_) were estimated following Farquhar et al. (1980) and Sharkey et al. (2007).

#### Light curve (A/Q) analysis

The light curve (A/Q) was measured to determine the photosynthetic response of the plant to a series of light intensities by following the Ye et al. (2013, 2021) model. Measurements were recorded at [CO_2_] ref of 410 μmol mol^−1^, with a series of PAR values of 0, 25, 50, 100, 150, 250, 300, 400, 500, 600, 800, 1,000, 1,200, 1,500, 1,600, 1,800, and 2,000 μmol photons m^−2^ s^−1^ with a range of 120–210 s at each PAR level. Prior to measurements, leaves were acclimated to the leaf chamber environment by placing them in the cuvette at a PPFD of 1,500 μmol photons m^−2^ s^−1^. The A/Q curve was used to determine key photosynthetic parameters, including light compensation point (Ic), quantum yield efficiency (AQY), light saturation (I_sat_), and maximum photosynthetic (A_max_). Additionally, dark respiration (R_d_) was also measured at a point when PAR = 0.

#### Photosynthetic induction and relaxation: non-photochemical quenching

The plants were placed overnight in a dark chamber prior to measuring chlorophyll fluorescence on tagged flag leaves. Minimum fluorescence (Fo) and maximum fluorescence (Fm) were recorded in the dark, followed by recording minimum fluorescence (Fo′) and maximum fluorescence (Fm′) after exposure of leaves to 1,500 μmol photons m^−2^ s^−1^ PPFD using saturation light (Schreiber et al., 1995), with dark pulses at 180-s intervals (LI-COR, Nebraska, USA). The intensity and duration of the saturation light pulse were >7,000 μmol photons m^−2^ s^−1^ and 0.8 s, respectively; modulation and filter were set to 20 kHz and 50, respectively. The duration of dark pulse was 6 s with 1-s pre-time and 4-s post-time with 6 μmol m^−2^ s^−1^ intensity of far-red light.

### SNP calling and diversity analysis

Genomic DNA was extracted from fresh leaves using the DNeasy Plant Mini kit (QIAGEN, Hilden, Germany) for whole-genome sequencing. All accessions were sequenced by Novogene, California, USA with an average depth of ~20×. A total of 6.5 million SNPs were detected after alignment of raw reads against the reference rice genome cv. Nipponbare (IRGSP 7.0) using the GATK 4.5.0.0 (BD Van Der Auwera and O’Connor, 2020) and used for genetic studies. The SNP dataset was deposited in Gramene (https://oryza.gramene.org) and published in release 8 (Kumar et al., 2021). For quality control, SNPs were filtered using PLINK v2.0. SNPs with a missing call rate greater than 20%, individuals with a missing accession rate greater than 20%, and SNPs with a minor allele frequency (MAF) less than 5% were excluded for further analyses. The resultant dataset contained approximately 9.0 million SNPs. This dataset was further pruned using a sliding-window linkage disequilibrium (LD)-based approach (10-kb window, 1-kb step, *r*^2^ > 0.2) based on a diverse rice panel rather than within a single subpopulation or individual chromosome. After LD-based pruning, 3.7 million out of 6.5 million SNPs were retrieved, reflecting the removal of strongly correlated markers within local LD blocks rather than aggressive genome-wide thinning. These SNPs were used for downstream genetic studies.

Principal component analysis (PCA) was performed using “GAPIT3” to classify subpopulations in the URMC panel (Wang and Zhang, 2021). The population structure analysis was conducted using admixture (Alexander et al., 2009) based on squared iterative methods and quasi-Newton convergence acceleration method to estimate the maximum likelihood of individual ancestries, with cross-validation (CV) 10 for one to 10 groups. The lowest cross-validation error estimated to determine the optimal population size and accessions clustering was visualized using a bar plot by applying Q matrices.

### Association and allelic profiling analyses

Genome-wide association analysis was performed using four statistical models—MLM, MLMM, FarmCPU, and BLINK—implemented in the R package “GAPIT3” (Wang and Zhang, 2021) with a stringent logarithmic odds (LOD ≥ 8.0) threshold to identify SNPs associated with the target trait. The LOD is based on Bonferroni’s correction method, which is calculated by dividing the SNPs that had a significance level of threshold (0.05) by the total number of effective SNPs (3.7 million), treating each SNP as independent in association analysis. The significant traits associated with SNPs were assigned as quantitative trait nucleotides (QTNs) for further analysis. The conditional phenotypic variance explained (PVE) of candidate QTNs were calculated using the linear mixed model for QTN j and can be written as.


$${\text{PVE}}_{\text{Conditional},j}=(R_{\text{full}}^{2}-R_{\text{reduction},j}^{2})\times 100\%$$

where $R_{\text{full}}^{2}$ is the conditional $R^{2}$ of the mixed model with all QTNs + PCs and $R_{\text{reduction },j}^{2}$ is the conditional $R^{2}$ of the same model by removing QTN j. Furthermore, allelic fingerprinting analysis of the identified QTNs was conducted to identify favorable alleles (those with positive contribution to photosynthesis) and unfavorable alleles (those with negative contribution to photosynthesis). This analysis was performed by selecting 50 contrasting accessions having high (>28 μmol m^−2^ s^−1^) and low (<18 μmol m^−2^ s^−1^) Pn values. Based on the distribution of favorable and unfavorable SNP alleles, SNP selection accuracy and efficiency were calculated by following previously reported equations (Su et al., 2016; Ravelombola et al., 2019; Riaz et al., 2023).

### Candidate gene analysis

The 200-kb flanking genomic region on either side of GWAS-derived SNPs was screened for candidate gene mining using the Nipponbare reference genome IRGSP 7.0 (Kawahara et al., 2013). The flanking region was selected based on average linkage disequilibrium blocks across subpopulations, as mentioned by Mather et al. (2007). Candidate genes were prioritized based on 1) previously identified genes contributing to photosynthesis and carbon metabolism retrieved from Database for Annotation, Visualization and Integrated Discovery (DAVID) and rice genome annotation project (Sherman et al., 2022; Hamilton et al., 2024). 2) Haplotype analysis of the identified genes was performed using geneHapR with SNPs retrieved from the corresponding genomic regions (Zhang et al., 2023). This analysis was conducted using the same set of accessions as used in the GWAS, and accessions were subsequently grouped based on mean photosynthetic rate (Pn) for each haplotype. 3) Orthologous genes in *Arabidopsis* (*Arabidopsis thaliana*), maize (*Zea mays*), and sorghum (*Sorghum bicolor*) were identified using the R package orthogene and Ensembl Plants BioMart (https://plants.ensembl.org/index.html; Kinsella et al., 2011) with additional confirmation from the Rice Genome Annotation Project (https://rice.uga.edu/) to strengthen functional annotation and cross-species inference. 4) Transcription factor (TF) binding sites within 2-kb upstream of candidate genes were identified using the Plant Transcriptional Regulatory Map database (PlantRegMap) with the “motif” method, “Target (retrieve TFs)” mode, and a significance threshold of p < 1 × 10^−6^ (Tian et al., 2020). The resulting TF–target interactions were used to construct and analyze gene regulatory networks in Cytoscape (Doncheva et al., 2018).

### Genomic prediction studies

The SNPs with LOD ≥ 4 from BLINK-GWAS models were selected for genomic selection to compute genomic estimated breeding value (GEBV). A moderate LOD threshold was selected by considering the polygenic nature of the trait, where multiple small-effect loci contribute to variation; stringent threshold parameters may exclude true associations. The GP was estimated using Bayesian methods: Bayes A (BA), Bayes B (BB), Bayes LASSO (BL), genomic best linear unbiased prediction methods (gBLUP), and random forest (RF). These methods were implemented in R-based packages “BGLR” (Pérez-Rodríguez and De Los Campos, 2022), “GAPIT3”, “RandomForest” (Breiman, 2001), and “Kernlab” (Karatzoglou et al., 2004) with iterations of 1,000 by following Riaz et al. (2023) and Shi et al. (2016).

Furthermore, GMStool, a machine/deep learning-based pipeline, was applied using SNPs derived from BLINK-based GWAS with a lower threshold (LOD ≥ 1) to identify optimum markers for predicting Pn (Jeong et al., 2020). GMStool selected the SNPs using ridge regression best linear unbiased prediction (RRB) and bootstrap trees (BTS) methods and predicted genomic value using a deep neural network and a convolutional neural network. Heuristic iterations, fivefold cross-validation, and pre-filtering of GWAS-derived SNPs were applied to maintain the model stability and reduce false-positive risk. The accuracy of these models was estimated using Pearson’s correlation coefficient (r) between GEBV from training and testing sets, and box plots were plotted using ggplot2.0 (Wickham, 2011).

Different LOD thresholds were applied depending on the analytical objective. Applying the stringent GWAS threshold may have limited the SNP set primarily to major-effect QTNs, potentially excluding biologically meaningful minor-effect SNPs. Using moderate to lower thresholds could help retain additional loci that may interact epistatically, reduce dimensionality, and control model overfitting, potentially improving the stability and predictive accuracy of genomic prediction and machine learning models for Pn.

## Results

### Genetic diversity analysis of Pn among URMC accessions

The rice mini-core collection of 217 accessions, representative of the broader USDA core and whole germplasm collection (1,794 and 18,709 accessions, respectively; Li et al., 2010), was used in this study. These accessions were collected from over 10 rice-growing geographic regions and represented six rice subpopulations. The photosynthetic rate (Pn), measured using the survey-based method with the LI-COR 6400 XT, was recorded in 181 out of 217 accessions; the remaining accessions either failed to germinate or did not reach the booting stage (**Supplementary Table 1a**). Most of these accessions were classified to the indica group (58), followed by the aus group (33) and the temperate (Tmp) japonica group (30). Both admixture (Admix) and tropical (Trp) japonica groups had the same number, “28”, of accessions, while the lowest number, “4”, of accessions were assigned to the aromatic group (**Supplementary Figure 1a**).

**Figure 1:**
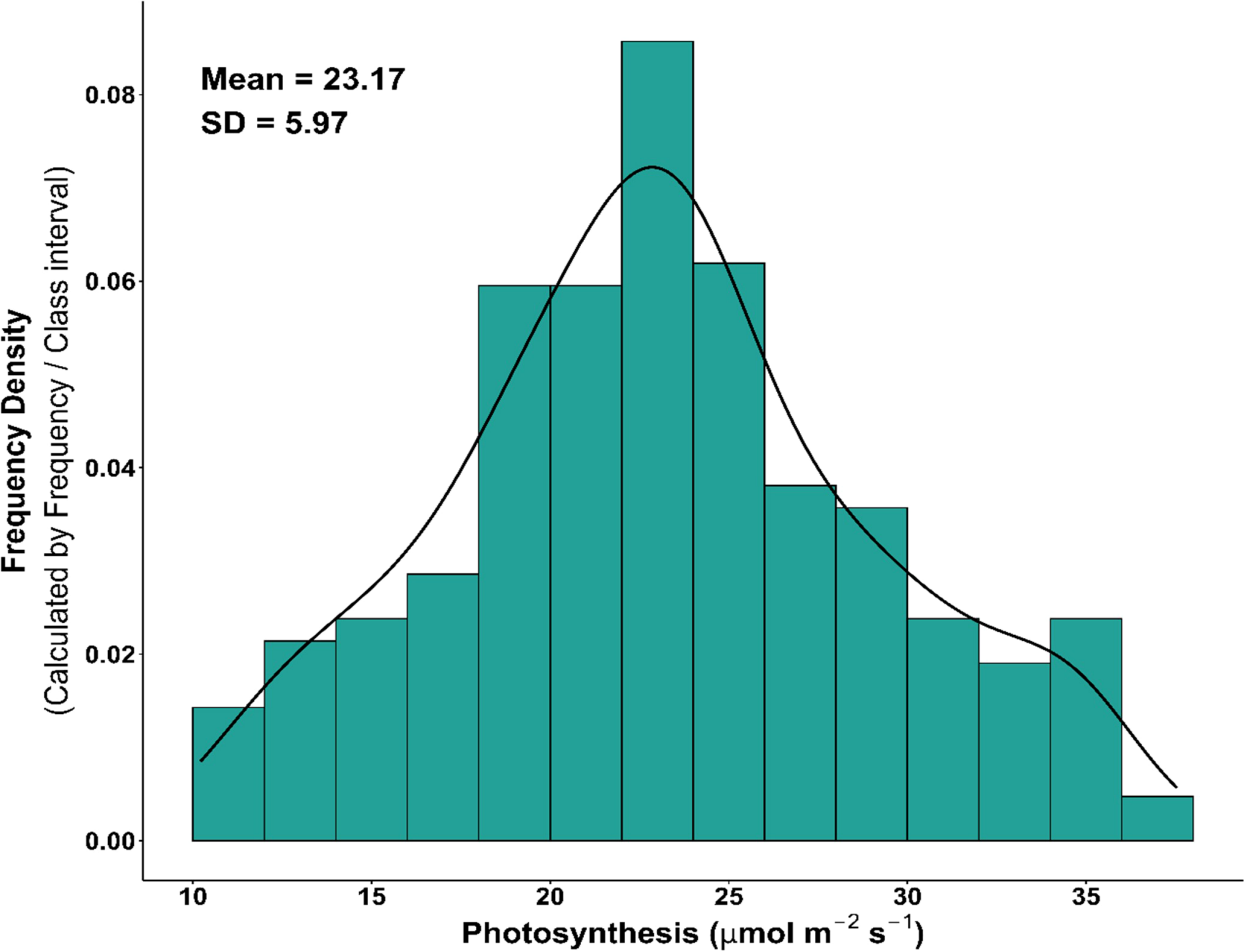
Frequency distribution of photosynthesis ranging from 10 to 37 μmol m^−2^ s^−1^ across diverse rice accessions. Each bar indicates the number of accessions present in specific Pn class. The bell-shaped histogram suggests that photosynthesis follows a normal distribution.

**Figure 2:**
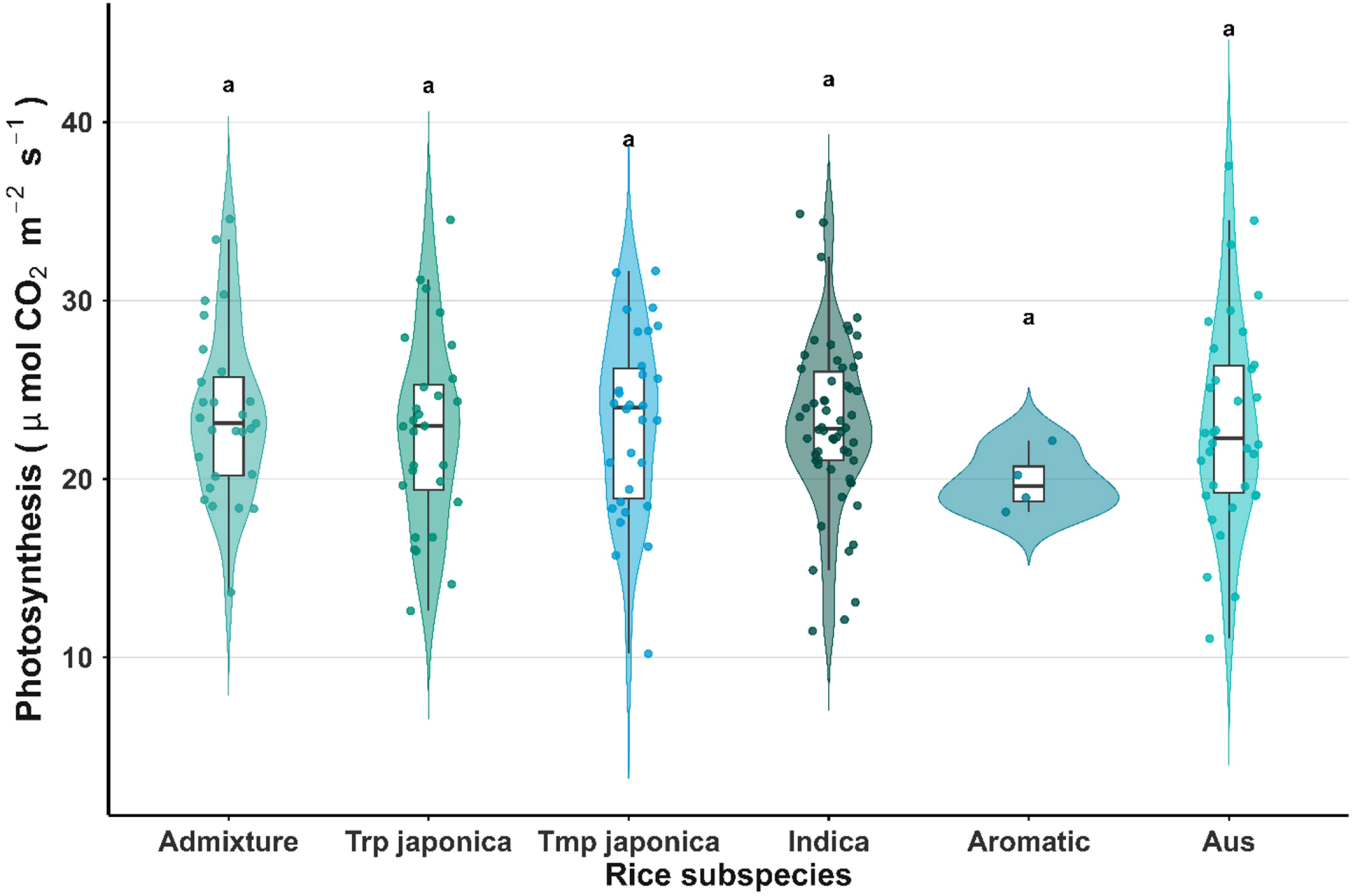
Photosynthesis rate variation across rice subspecies. The x-axis represents six rice subspecies, while the y-axis shows photosynthesis rates (μmol m^−2^ s^−1^). The violin chart indicates the range of photosynthesis values for a given subspecies, with the minimum and maximum values. Subspecies sharing the same lowercase letter are not significantly different based on ANOVA followed by Tukey’s Honest Significant Difference (HSD) test (p < 0.05).

The histogram plot revealed an approximately symmetrical distribution of Pn, ranging from 10 to 37 μmol m^−2^ s^−1^ (**Figure 1**). Within this overall range, broad Pn distributions were observed across most subpopulations—Admix 13.6 to 34.5 μmol m^−2^ s^−1^, aus 11.05 to 37.53 μmol m^−2^ s^−1^, indica 11.48 to 34.85 μmol m^−2^ s^−1^, Tmp japonica 10.21 to 31.66 μmol m^−2^ s^−1^, and Trp japonica 12.61 to 34.52 μmol m^−2^ s^−1^—indicating substantial genetic diversity and adaptive variation within these groups. In contrast, the aromatic group exhibited a narrower range of Pn (18.15 to 22.14 μmol m^−2^ s^−1^), implying lower variability and limited adaptive breadth (**Figure 2**). In addition to the histogram of Pn, the correlation plot (**Supplementary Figure 1b**) showed that Pn was positively correlated with stomatal conductance, transpiration rate, and CO_2_ conductance, but negatively correlated with VpdA. Similarly, stomatal conductance, transpiration rate, and CO_2_ conductance were all positively correlated with one another, while each exhibited a negative correlation with VpdA. Six accessions (GSOR311153, GSOR311181, GSOR311249, GSOR311586, GSOR310080, and GSOR311046) were identified as having the highest Pn values (>34.0 μmol m^−2^ s^−1^), while three accessions (GSOR311544, GSOR310846, and GSOR310861) showed the lowest Pn values (<12.0 μmol m^−2^ s^−1^). The Pn variability in this study indicated that sufficient phenotypic variation for genome-wide association and genomic prediction analyses was present in the population.

Five rice accessions (GSOR310045, GSOR310080, Zhe733, N22, and Cypress) were selected based on the mentioned criteria for exploratory physiological studies used to plot A/Q (light response) and A/Ci (CO_2_ response) curves. Nipponbare was excluded from the final analysis due to high variability in measurements within the accession. As each measurement required approximately 30–45 minutes per plant, this approach allowed for a comprehensive assessment of photosynthetic parameters. These analyses revealed marked phenotypic differences in light-use efficiency, carboxylation capacity, and photorespiration. Furthermore, chlorophyll fluorescence (NPQ) measurements confirmed variability in photoprotective capacity and PSII stability. Together, the survey-based screening and detailed response-curve analyses demonstrated ample photosynthetic and photoprotective diversity within the mini-core, providing a strong foundation for dissecting the genetic basis of photosynthetic efficiency in rice.

#### Genotype response to light intensities (A/Q curve)

**Figure 3:**
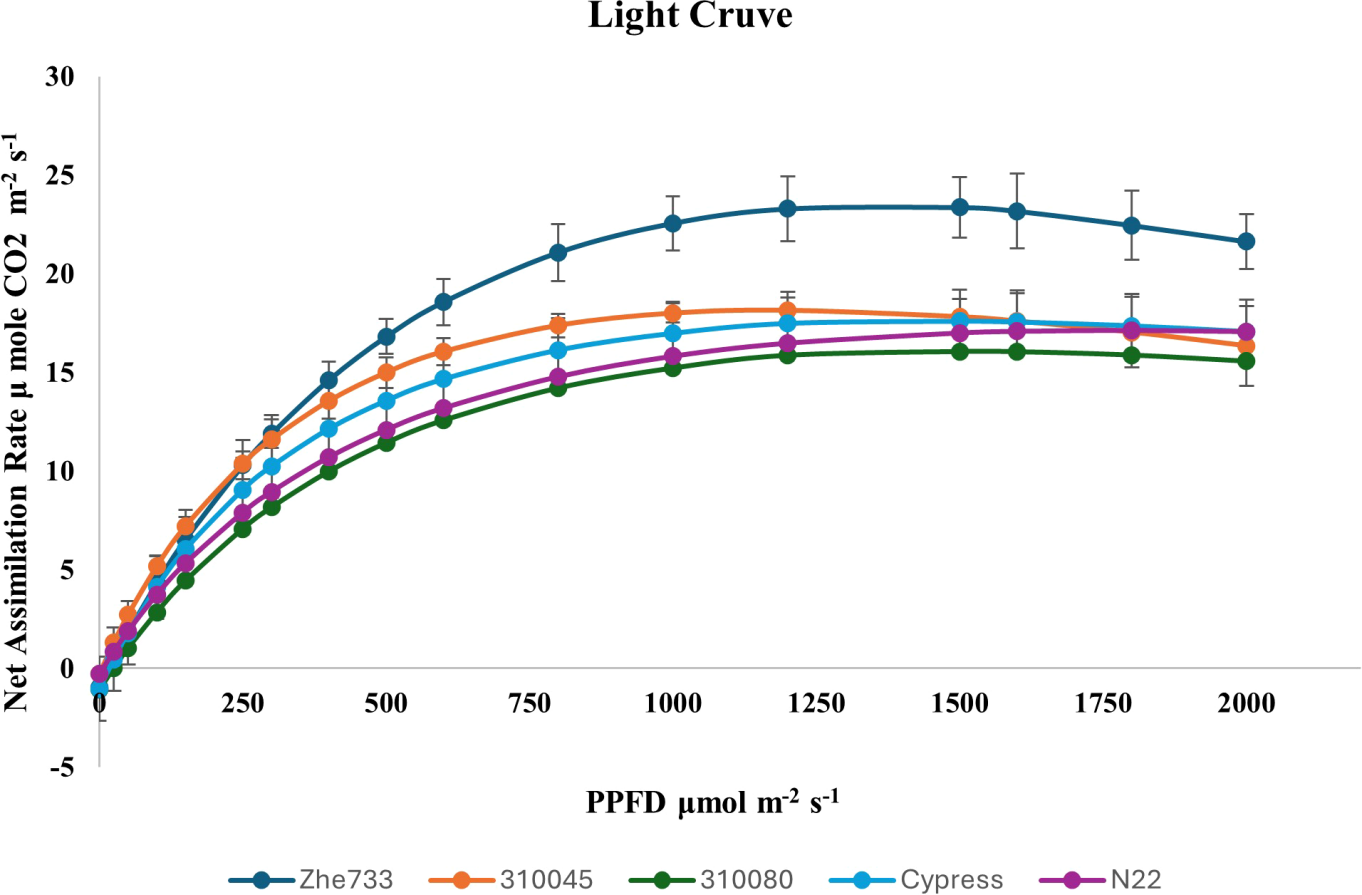
Photosynthetic response of various accessions to increasing light intensity. Zhe733 and 310045 exhibited a rapid response to an increase in light levels, whereas 310080 showed the lowest response. At saturated light intensity, Zhe733 had the highest Pn value, while 310080 had the lowest. Cypress and N22 demonstrated a moderate response to increasing light intensity, with Cypress, N22, and 310045 maintaining moderate Pn values at saturation. Overall, Pn increased with increasing light intensity from darkness to saturation, after which photoinhibition led to a decline in Pn.

Significant phenotypic variation was observed across all phases of the A/Q curve. The highest light-use efficiency under low-light conditions was observed in Cypress and GSOR310045, with initial slope values approximately 6.2 µmol CO_2_ µmol^−1^ photons, followed by Zhe733, N22, and GSOR310080. The highest photosynthetic capacity at light saturation was recorded in Zhe733 (23.45 µmol CO_2_ m^−2^ s^−1^), whereas the lowest photoinhibition (1.40) was observed in Cypress, indicating greater PSII stability and resilience to high-light stress. The highest light-saturation intensity (1.79 × 10^3^), lowest light-compensation point (6.28), and low dark respiration (0.27) were observed in N22, suggesting superior adaptation to low-light environments (**Figure 3**; **Supplementary Table 2**).

#### Genotype response to CO_2_ level (A/Ci curves)

**Figure 4:**
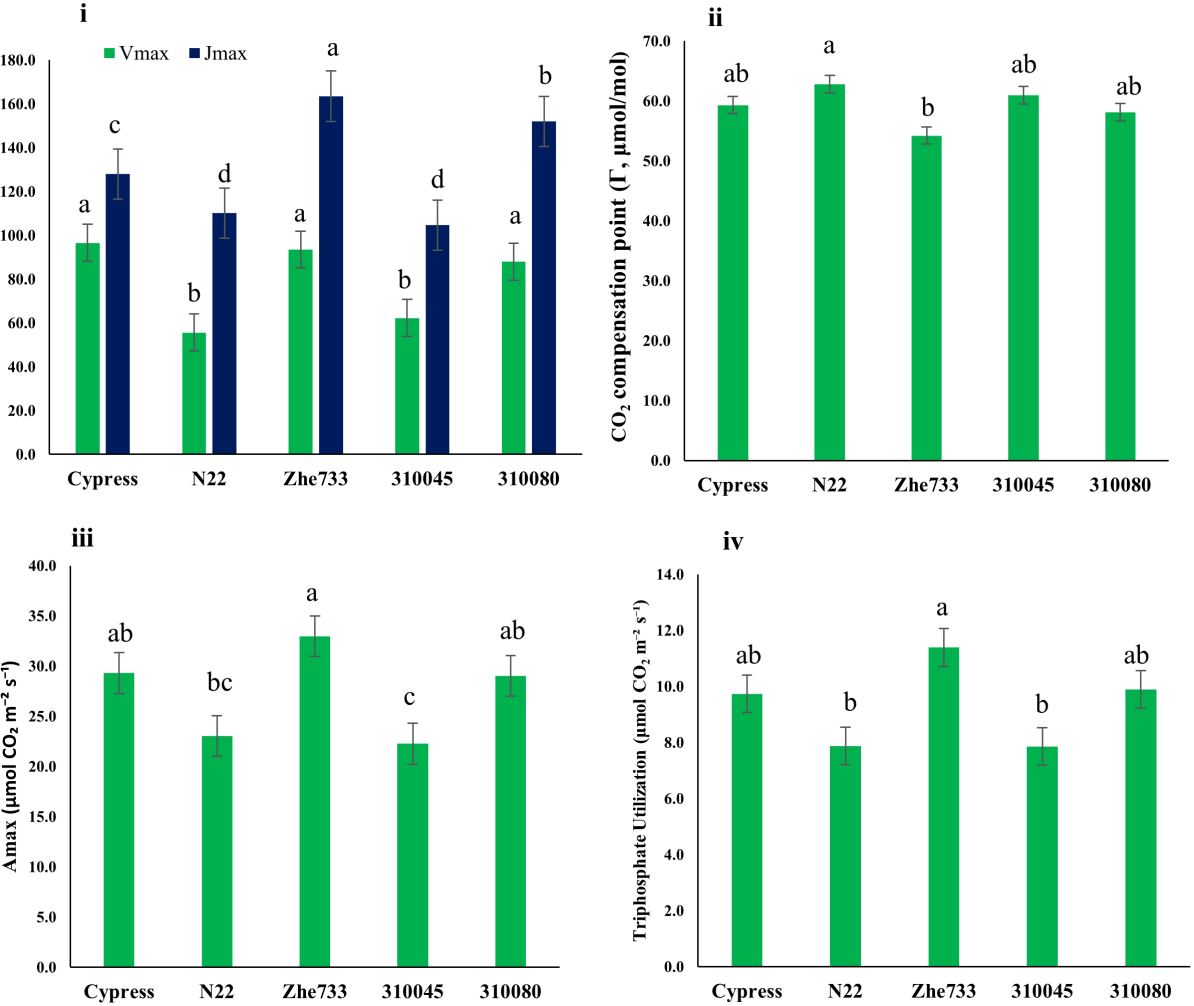
Rice accessions (x-axis) analyzed based on photosynthetic capacity parameters (y-axis) derived from A/Ci response curve. i) Vmax (maximum rate of carboxylation, mol m^−2^ s^−1^), and Cypress had highest Vmax, followed by Zhe733 and 310080; however, N22 and 310045 showed lowest Vmax. For Jmax (maximum rate of electron transport μmol m^−2^ s^−1^), Zhe733 showed highest value, followed by 310080 and Cypress, but N22 and 310045 displayed lowest Jmax value. ii) The CO_2_ compensation point (Γ*) showed N22 had the highest CO_2_ compensation point (Γ*) followed by 310045, whereas Zhe733 had the lowest among the accessions. iii) A_max_ (CO_2_ uptake in saturating [CO_2_] and light, μmol m^−2^ s^−1^) and iv) Triphosphate utilization (μmol CO_2_ m−2 s^−1^) showed similar results and indicated Zhe733 had highest value followed by 310080 and Cypress, while N22 and 310045 had lowest value in response to CO_2_ gradient concentrations (μmol mol^−1^). Accessions sharing the same letter are not significantly different based on ANOVA followed by Tukey’s HSD test (p < 0.05).

Significant phenotypic variation was observed in photosynthetic response to intercellular CO_2_ concentration. Superior CO_2_ fixation capacity was observed in Zhe733 and Cypress, with the highest carboxylation (Vmax ≈ 93–96 mol m^−2^ s^−1^) and electron transport rates (Jmax ≈ 140–160 µmol m^−2^ s^−1^), along with elevated A_max_ and TPU values, indicating efficient Rubisco regeneration and high photosynthetic potential. In contrast, the highest CO_2_ compensation point (Γ*) was observed in N22, suggesting greater photorespiration, while 310045 exhibited the lowest overall CO_2_ assimilation efficiency. These results highlight strong carbon fixation ability and low photorespiratory loss in Zhe733, and balanced efficiency was observed in Cypress across multiple parameters (**Figure 4**; **Supplementary Figure 2**).

#### Non-photochemical quenching

**Figure 5:**
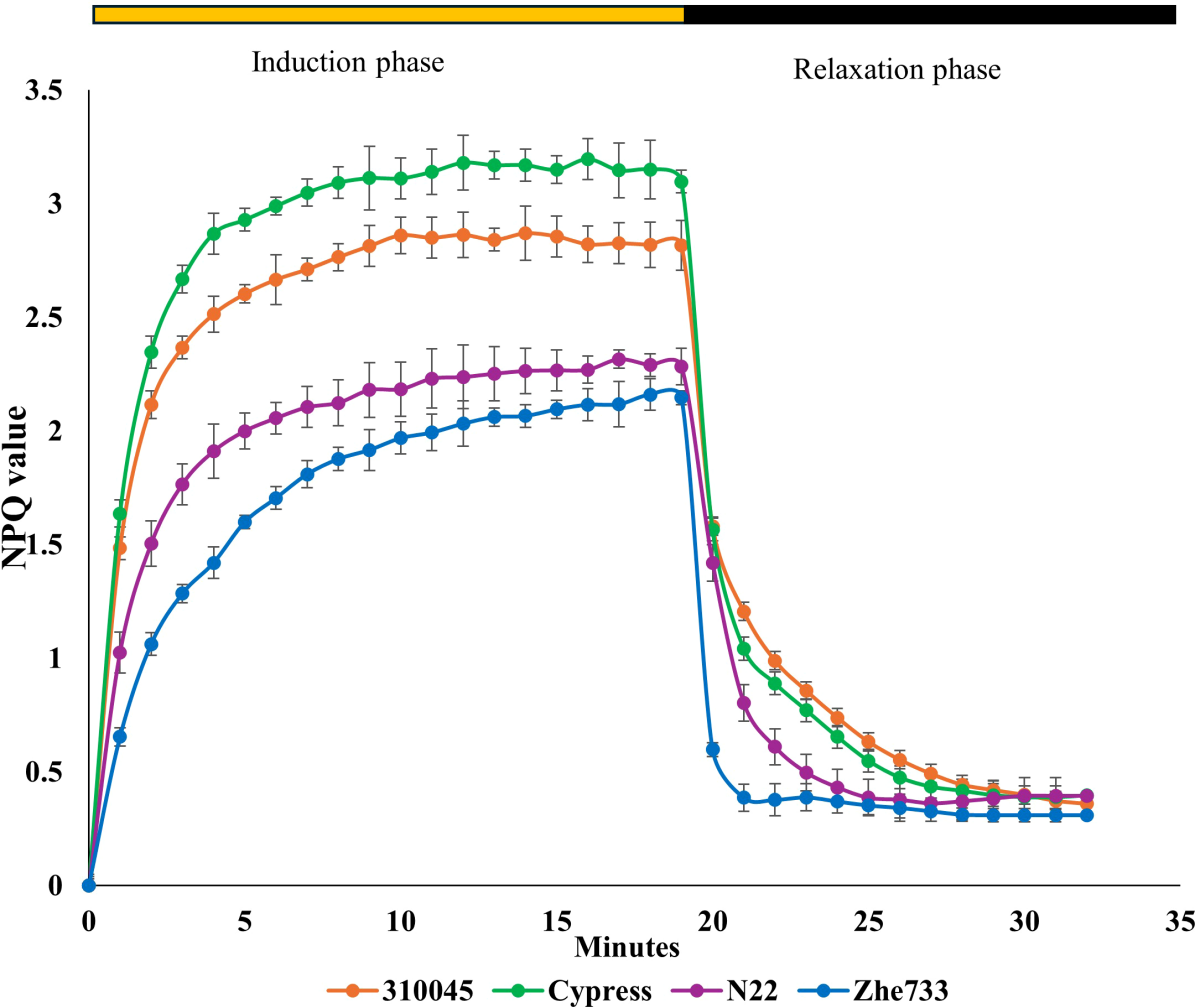
NPQ induction and relaxation curves of various accessions carried out in a period of 30 minutes after dark adaptation of plants. NPQ values rose during induction phase and declined during relaxation phase. Cypress and 310045 showed abrupt increases in NPQ compared to Zhe733 and N22. A similar pattern was also observed in light zone of induction phase. During the relaxation phase, NPQ rapidly declined in Zhe733 and N22 compared to Cypress and 310045, which showed a smoother decline in values. After 10 minutes in relaxation phase, all accessions showed similar NPQ pattern. NPQ, non-photochemical quenching.

Significant phenotypic differences were observed in NPQ induction and relaxation dynamics under high actinic light conditions. The highest NPQ magnitude and fastest induction were observed in Cypress, indicating the strong and rapid activation of photoprotective mechanisms. Moderately high NPQ with slightly slower activation was observed in GSOR310045, whereas lower NPQ and slower induction were observed in Zhe733 and N22, reflecting weaker energy dissipation capacity. During the dark relaxation phase, prolonged NPQ retention was observed in Cypress, suggesting sustained photoprotection, whereas rapid relaxation was observed in Zhe733 and N22, indicating quicker recovery to protect the photosystem (**Figure 5**). GSOR310080 was excluded from the final NPQ analysis because highly erratic NPQ induction and relaxation behaviors were observed, leading to poor model fits and non-convergent parameter estimates despite repeated measurements.

Overall, reduced responsiveness to high-light intensity was observed in Zhe733 and N22, allowing the maintenance of higher photosynthetic efficiency, whereas superior and more stable photoprotective efficiency was demonstrated by Cypress, albeit with a trade-off in photosynthetic efficiency.

### Genetic diversity analysis

**Figure 6:**
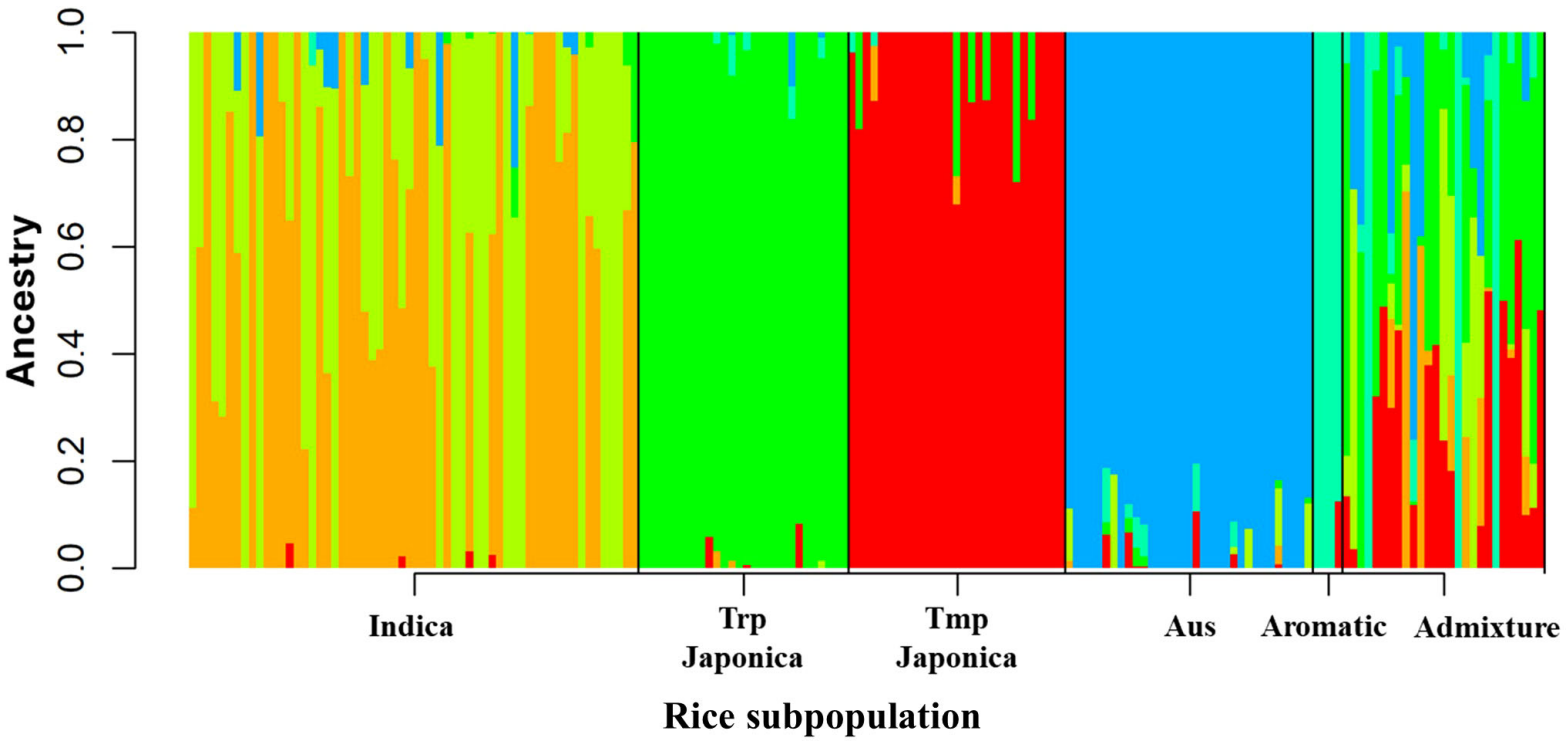
Population structured analysis of URMC diversity panel. Bar plot of accession membership coefficients for the genetic clusters inferred using admixture (K = 6) based on 3.7 million SNPs. Individual membership coefficients (Q) were sorted within each cluster. Six clusters are shown in gold, lime, turquoise, green, red, and blue to represent genetic diversity in each subpopulation.

To analyze the population structure, ancestry analysis and PCA were conducted using the SNP dataset of the panel. Six genetically distinct subpopulations were identified based on the lowest CV error, with K values ranging from 02 to 10. Based on the kinship matrix, accessions having a Q matrix value >70% were assigned to the respective group, while the remaining accessions were considered as admixture (**Figure 6**). The admixture analysis revealed that the indica and admix groups exhibited the highest levels of genetic diversity compared with the japonica, aus, and aromatic subpopulations. Within the japonica subpopulation, both Trp and Tmp were found to be genetically distinct, with the Trp group exhibiting a lower diversity than the Tmp subpopulation. The aromatic and aus subpopulations maintained their unique genetic makeup, although the aus subpopulation showed some genetic similarity to both the japonica and indica subpopulations. These results were further corroborated via PCA, which also classified the population into six subpopulations (**Supplementary Figure 3**). Overall, these results suggested that each subpopulation maintains a relatively stable genetic boundary with its own genetic composition.

#### Association studies

The observed phenotypic and genetic variations for Pn, as indicated by histogram and population structure analyses, suggested that the panel was suitable for association analyses to explore underlying genetic mechanisms across diverse accessions. Genome-wide analysis was performed using four models (MLM, MLMM, FarmCPU, and BLINK), which collectively identified 18 putative QTNs (LOD ≥ 8.0) for Pn (**Table 1**; **Supplementary Figures 4a, b**).

**Table 1:** Significantly associated SNPs for photosynthesis identified using GWASs.

Model	QTN	Chromosome	Position	P-value	LOD	MAF (%)	Effect	Phenotype_variance_explained (%)	Conditional_phenotype_variance_explained (%)
BLINK	1-2050328	1	2050328	5.90E−10	9.23	0.11	−2.43	6.699	3.24
BLINK	1-42455469	1	42455469	2.10E−10	9.68	0.07	2.81	8.7161	1.13
BLINK	3-15920400	3	15920400	1.70E−10	9.77	0.41	2.08	5.1619	2.31
BLINK	3-31894376	3	31894376	3.40E−11	10.46	0.47	−3.57	10.4285	0.74
BLINK	6-24708134	6	24708134	3.50E−11	10.46	0.15	−2.92	7.2378	0.46
BLINK	9-6063219	9	6063219	4.60E−13	12.33	0.11	3.22	14.3726	2.25
BLINK	9-9565539	9	9565539	5.20E−19	18.28	0.09	−4.49	17.4621	2.11
BLINK	11-21301005	11	21301005	1.20E−09	8.93	0.09	2.61	8.7178	1.71
BLINK	12-11380740	12	11380740	1.60E−13	12.79	0.22	3.33	10.5073	5.02
FarmCPU	1-1562037	1	1562037	2.60E−10	9.59	0.22	2.27	0.0002	1.34
FarmCPU	1-2050328	1	2050328	8.80E−10	9.05	0.11	−2.16	10.7261	3.24
FarmCPU	1-3000323	1	3000323	3.20E−09	8.49	0.24	1.92	1.7146	1.73
FarmCPU	2-24283728	2	24283728	7.60E−11	10.12	0.23	1.57	0.0007	1.28
FarmCPU	3-3891437	3	3891437	2.30E−09	8.63	0.19	−3.1	0.0003	1.46
FarmCPU	4-34940170	4	34940170	4.50E−13	12.34	0.08	−3.03	6.897	1.67
FarmCPU	6-27876287	6	27876287	1.30E−11	10.9	0.19	−2.06	0.0004	0.92
FarmCPU	7-15847900	7	15847900	1.60E−14	13.79	0.44	3.1	0.0007	2.21
FarmCPU	8-26736462	8	26736462	7.50E−11	10.13	0.23	−1.68	0.0009	2.55
FarmCPU	12-11380740	12	11380740	2.00E−15	14.7	0.22	3.09	1.4776	5.02
MLMM	12-4544265	12	4544265	5.30E−10	9.27	0.1	−5.56	8.1942	0.17

GWASs, genome-wide association studies; QTN, quantitative trait nucleotide; LOD, logarithmic odds; MAF, minor allele frequency.

**Figure 7:**
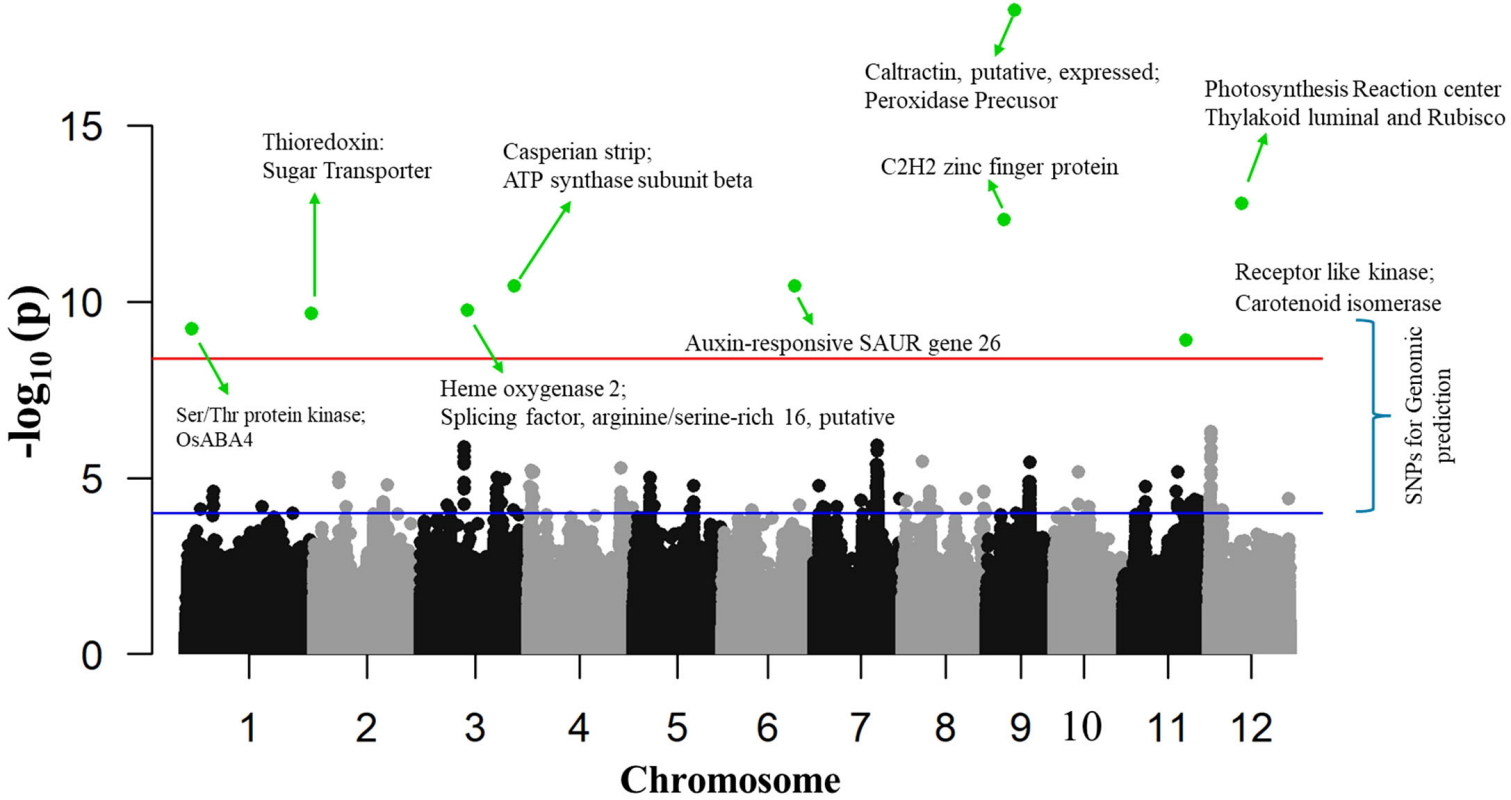
Manhattan plot of SNPs associated with photosynthesis rates (μmol m^−2^ s^−1^) using the BLINK model. The red line represents the significance threshold (p = 1 × 10^−8.0^) determined by Bonferroni’s correction method. The green dots indicate significant associated SNPs, labeled with annotated genes located in close proximity that may contribute to photosynthesis. The blue line represents the suggestive significance threshold (p = 1 × 10^−4.0^) used for selecting SNPs in genomic prediction using various machine learning models.

**Figure 8:**
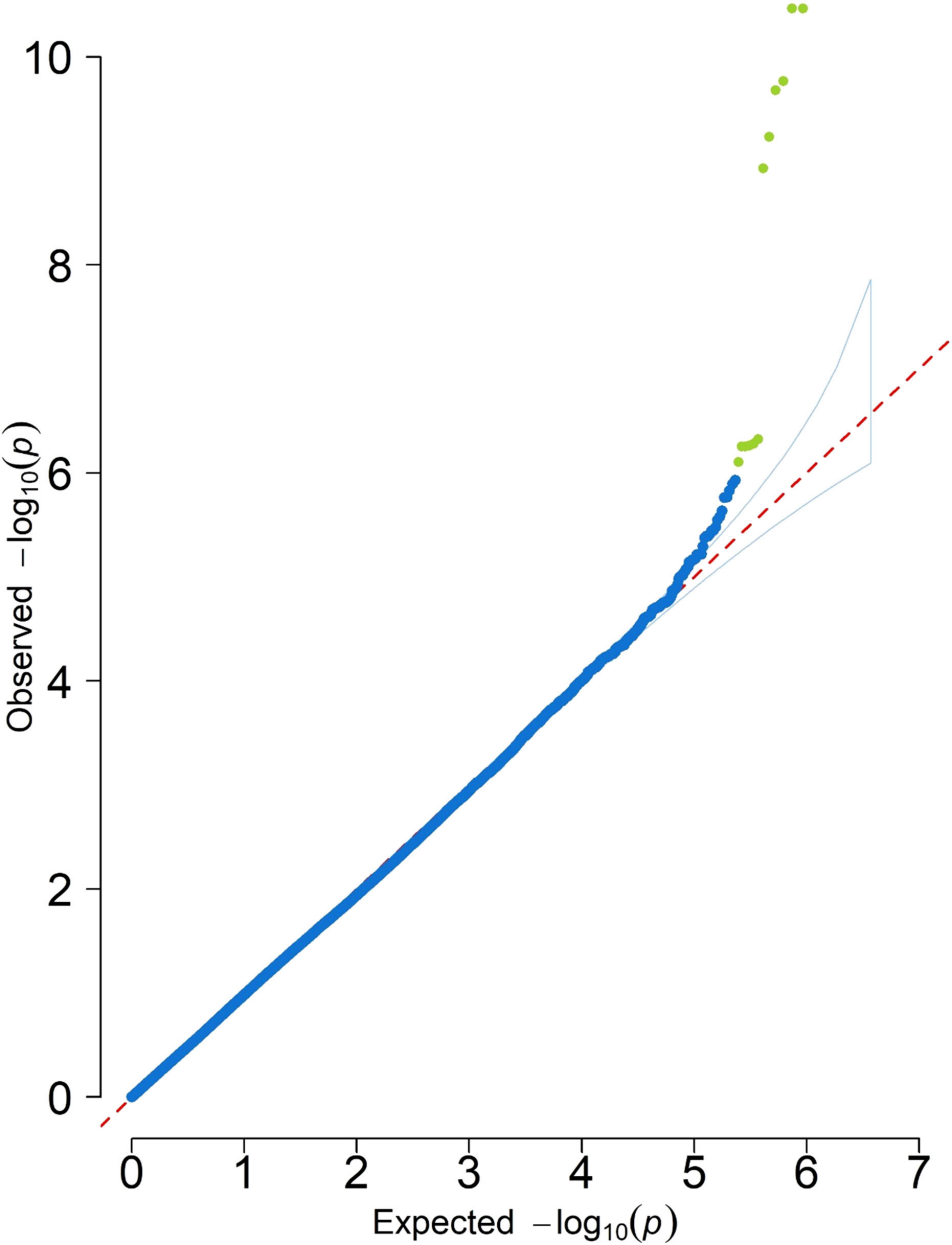
Quantile–quantile (Q-Q) plot for SNPs associated with photosynthesis rates (μmol m^−2^ s^−1^) in the BLINK model. Blue dots indicate SNPs that follow the expected distribution, while green dots represent SNPs that are more significant than expected.

These putative QTNs showed PVE values ranging from 0.0002% to 17.46%, reflecting a combination of small-, moderate-, and major-effect loci contributing to variation in Pn. Within the BLINK model, nine putative QTNs were detected, with PVE ranging from 5.17% to 17.46%, representing moderate- to major-effect loci. Notably, 9-9565539 (17.46%) and 9-6063219 (14.37%) were major-effect QTNs, indicating genomic regions with strong influence on Pn. QTNs on chromosomes 3 and 12 also showed PVE > 10%, underscoring their potential role in photosynthetic efficiency (**Figures 7**, **8**). Within the FarmCPU model, 10 QTNs were identified, but mostly QTNs explained low PVE (<5%), suggesting polygenic control with many small-effect loci. Only two QTNs, 1-2050328 (10.73%) and 4-34940170 (6.90%), exhibited moderate to high effects, implying that FarmCPU is sensitive to detecting both major and minor associations. Several QTNs (e.g., on chromosomes 2, 3, 6, 7, and 8) showed very low PVE, possibly due to stringent model correction or small-effect signals. The MLMM model detected one QTN (12-4544265) with 8.19% PVE, classifying it as a moderate-effect locus.

Furthermore, conditional PVE values were estimated by jointly fitting the 18 putative QTNs to assess the independence of their effects. The conditional PVE values ranged from 0.17% to 5.02%, with most candidate QTNs explaining approximately 0.2%–3.2% of the phenotypic variance. Several QTNs with PVE values close to 0.00% were found to exhibit substantial conditional PVE (0.92%–2.55%), indicating that these loci contribute to variation in Pn when considered jointly with other QTNs. The lowest conditional PVE was observed for QTN 12-4544265 (0.17%), despite its moderate PVE, suggesting that its effect largely overlaps with that of other loci. In contrast, the highest conditional PVE (5.02%) was observed for QTN 12-11380740, which also exhibited a high PVE, indicating a relatively independent and substantial contribution to photosynthesis. Other QTNs with high PVE (excluding 12-11380740) showed reduced conditional PVE (2.11%–3.24%), implying that their effects are partially shared with other loci. Overall, these results indicate that Pn is controlled by multiple QTNs with largely joint and partially overlapping effects rather than by a single dominant locus. These 18 putative QTNs were found to exhibit MAFs ranging from 0.07 to 0.46 in the BLINK model, 0.08 to 0.44 in FarmCPU, and 0.09 in MLMM, indicating that both rare and moderately common alleles may contribute to the observed variation in photosynthetic rate (Pn). QTNs with moderate MAF values (0.15–0.30) generally showed larger effect sizes and higher PVE, suggesting that alleles occurring at intermediate frequencies could have a more stable and substantial influence on Pn variability. In contrast, QTNs with very low MAFs (<0.10) generally had smaller effects, consistent with the limited contribution of rare alleles in population-level variation. Overall, these trait-associated putative QTNs suggested that genomic regions on chromosomes 1, 3, 11, and 12 may significantly contribute to Pn and could be valuable for marker-assisted breeding programs.

To further evaluate models’ performance for genomic prediction analyses across the population, quantile–quantile (Q-Q) plots were examined to explain the best fit model based on the distribution of observed p-values against the expected normal distribution of SNPs (**Supplementary Figure 4b**). These results suggested that BLINK is the best-fitted model and was selected for genomic prediction studies.

### Gene mining analysis for potential candidate genes

To further explore the functional relevance of the identified genomic regions, a total of 1,091 coding and non-coding genes were identified within ±200-kb flanking regions around each QTN, capturing genes likely in linkage disequilibrium (LD) with the associated loci. These regions encompass both coding and regulatory genes, potentially linked to photosynthesis (**Supplementary Table 3**).

On chromosome 1, the underlying genes associated with four QTNs (1-1562037, 1-2050328, 1-3000323, and 1-42455469) were found to include Bowman–Birk-type protease inhibitors (BBTI6–12), MYB transcription factors, multicopper oxidases, receptor kinases (WAK1 and WAK2), serine/threonine kinases, antioxidant enzymes (peroxidases and thioredoxin), heavy metal-associated domain proteins, and several expressed proteins. These genes are likely involved in photosynthetic stability, oxidative stress mitigation, and cellular protection, reflecting a balance between regulatory and defense mechanisms. For chromosome 2, the flanking region of QTN (2-24283728) was found to contain AP2-like transcription factors along with transport and metabolic enzymes, indicating coordination between gene expression regulation and primary metabolism.

A cluster of core photosynthesis-associated genes was identified in the flanking regions of QTNs located on chromosomes 3 (3-15920400, 3-31894376, and 3-38914377) and 12 (12-11380740, and 12-4544265). These genes include ribulose-phosphate 3-epimerase, malate dehydrogenase, Rubisco, ATP synthase subunit, cytochrome, flavin monooxygenase, and thylakoid lumenal 29.8-kDa protein, strongly suggesting involvement in carbon fixation, redox balance, and energy conversion processes during photosynthesis. The flanking genes of QTN (4-34940170) on chromosome 4 encode intracellular transporters, hydrolases, pyrophosphatases, heat shock proteins, and potassium efflux proteins, pointing toward adaptive responses in ion homeostasis and thermal stress regulation.

A functionally diverse set of genes was identified in the flanking region of QTN (6-24708134) on chromosome 6, including those involved in auxin signaling, potassium transport, and several transcription regulators such as MYB, helix–loop–helix (bHLH), and no apical meristem (NAM) proteins, together with cytochrome P450 genes. This region reflects cross-talk between hormone signaling, secondary metabolism, and stress response pathways. However, QTN 7-15847900 located at chromosome 7 displayed a high number of retrotransposable elements interspersed with a few coding genes, such as F-box proteins, kinases, and NAM proteins, suggesting structural genomic variability and potential epigenetic influence on nearby functional loci.

Similarly, a diverse gene landscape was also detected in the flanking region of QTN (8-26736462) on chromosome 8, encompassing intracellular transporters, lipid transfer/protease inhibitors, AP2 and NAM transcription factors, and pentatricopeptide repeat proteins, implicating roles in membrane remodeling, protein stabilization, and transcriptional regulation under variable physiological conditions.

Among all detected QTNs, two loci on chromosome 9 (9-9565539 and 9-6063219) stood out with particularly high LOD (18.28 and 12.33) and PVE (17.46 and 14.37) values, as well as contrasting effect sizes (−4.49 and 3.22). The genomic regions surrounding these QTNs harbor genes related to mitochondrial inner membrane translocase, oxidoreductase domain proteins, NAD-dependent epimerase/aldose 1-epimerase, F-box proteins (OsFBX311–316), serine/threonine protein phosphatases, Ras-related proteins, and various expressed or transposon-derived genes. Collectively, these genes are likely to contribute to energy metabolism, protein homeostasis, and stress signaling, thus maintaining photosynthetic efficiency and physiological stability under various environmental conditions.

### Haplotype and ortholog analyses for potential candidate genes

To identify the potential candidate genes associated with Pn, haplotype analysis was performed for genes identified from flanking genomic regions of the 18 putative QTNs. This approach further improved the power to detect significant regions by capturing linkage disequilibrium patterns, in which multiple SNPs are co-inherited as a haplotype block. Genes within QTN flanking regions were analyzed for SNP combinations, identifying 43 genes across 13 QTNs with haplotypes significantly associated with Pn (**Supplementary Table 4**). Chromosome 1 was found to harbor the highest number of genes (13) associated with four QTNs: “1-1562037 (four genes)”, 1-42455469 (four genes)”, “1-3000323 (three genes)”, and “1-2050328 (two genes)”. Chromosome 4 showed the second-highest number of genes (seven genes) associated with QTN “4-34940170”, followed chromosomes 9 and 11 each with six genes distributed across two QTNs “9-6063219 (two genes)” and “9-9565539 (four genes)”, respectively, and a single QTN “11-21301005”. Chromosome 6 contained five genes across two QTNs, “6-24708134 (one gene)” and “6-27876287 (four genes)”, while chromosome 3 harbored four genes linked to two QTNs (3-15920400 (three genes)” and “3-31894376 (one gene)”. Chromosomes 2 and 8 each contained a single gene linked to their respective QTNs, while, notably, no QTNs were identified on chromosomes 7 and 12.

**Figure 9:**
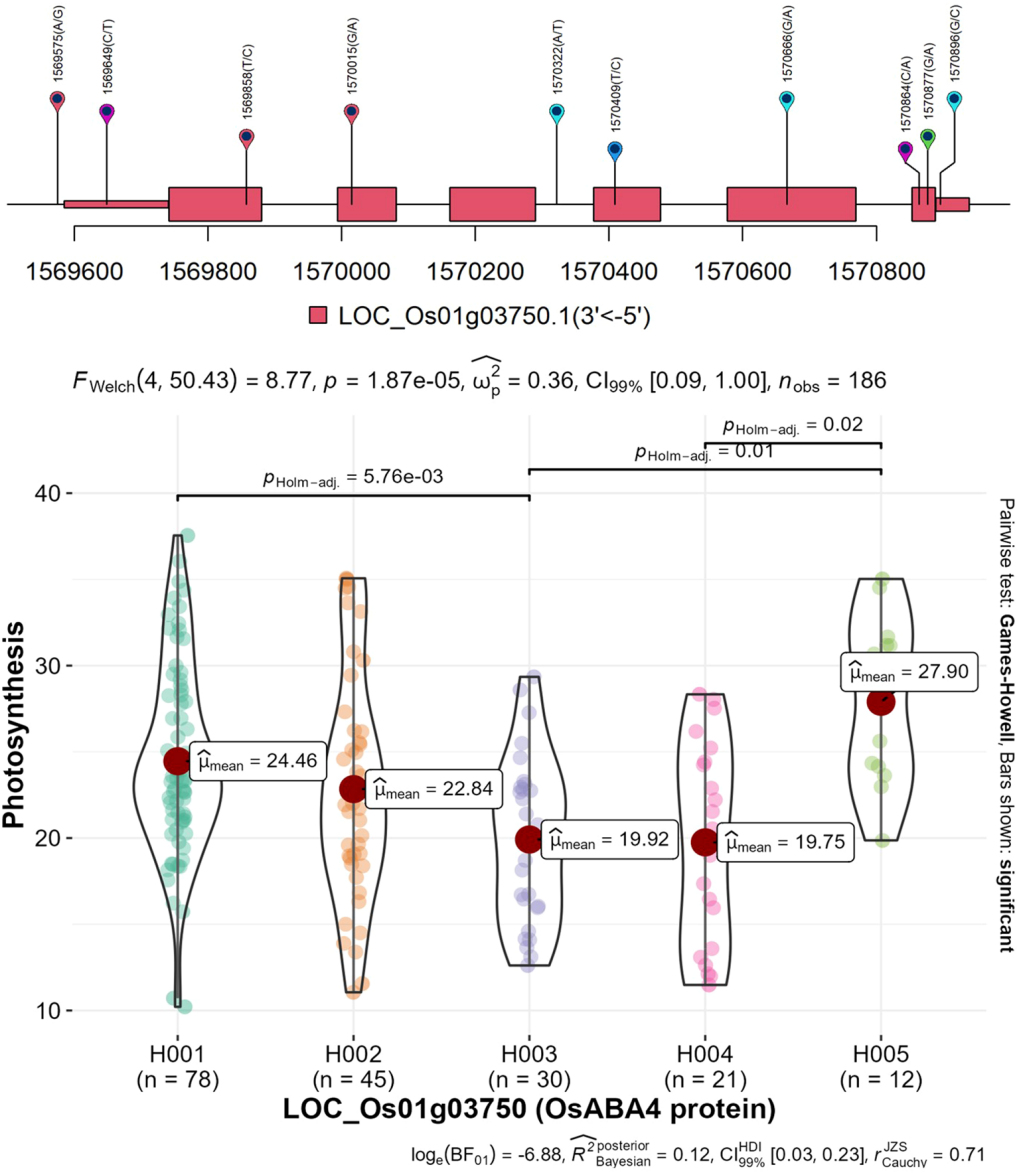
Gene haplotype analysis was performed based on GWAS results, followed by downstream analyses, including gene ortholog identification. The Games–Howell pairwise test was applied to determine substantial differences among haplotypes contributing to photosynthesis, with results visualized using violin plots. Holm’s method was used to adjust p-values for multiple comparisons, controlling the familywise error rate. a) LOC_Os01g044660: five haplotypes (H001–H005) were identified. Among them, H005 exhibited the highest mean Pn (27.90), which was significantly different from H004 (lowest mean Pn: 19.75) and H003. Additionally, H001 was significantly different from H003. GWAS, genome-wide association study.

These potential candidate genes were selected based on SNP haplotypes that caused significant differences in mean Pn values among accessions, with a threshold of at least five accessions per haplotype. For instance, LOC_Os11g36430, encoding a zinc finger protein, showed six haplotypes. Haplotype H005 exhibited the highest Pn value (30.69 μmol m^−2^ s^−1^ and n = 8), which was significantly different from H004, showing the lowest Pn value (17.79 μmol m^−2^ s^−1^ and n = 10). Similarly, LOC_Os11g36440, encoding amine oxidase (flavin-containing protein), also exhibited six haplotypes. Haplotype “H005” showed the highest Pn value (29.90 μmol m^−2^ s^−1^ and n = 9), followed by haplotype “H002” (25.63 μmol m^−2^ s^−1^, n = 16), both of which were significantly different from haplotype “H004” (17.23 μmol m^−2^ s^−1^, n = 9). Another gene, LOC_Os09g15590 encoding OsFBX316, exhibited six haplotypes, with haplotype “H005” showing the highest Pn value (33.55 μmol m^−2^ s^−1^, n = 7), which was significantly different from haplotypes “H001”, “H002”, and “H003”, having Pn values ranging from 23.26 to 22.26 μmol m^−2^ s^−1^. Detailed SNP information and haplotype analysis for all 43 genes are provided in the supplementary table (**Supplementary Table 12**) and supplementary figure (**Supplementary Figure 11**). Following the identification of 43 candidate genes, an *in silico* ortholog-based comparative analysis was performed by identifying *Arabidopsis*, maize, and sorghum orthologs to further prioritize these genes. A total of 50 paralogue expansions corresponding to 19 of the 43 rice genes were identified in *Arabidopsis*; these genes are reported to involve signaling, transportation, energy production, and cellular homeostasis pathways (**Supplementary Table 5**). There were no orthologs for the remaining 24 rice genes found in *Arabidopsis*. Among these, seven genes based on their putative functions were selected as key candidate genes for photosynthesis enhancement, and their orthologs were identified in both maize and sorghum (**Table 2**). LOC_Os01g03750 (**Figure 9**) and LOC_Os11g36440 encode proteins involved in photosystem protection via ABA and carotenoid metabolic pathways, LOC_Os02g40030 and LOC_Os09g15330 encode carbohydrate transporters, and LOC_Os06g45970 and LOC_Os01g04460 encode an auxin-responsive protein and a kinase, respectively. In addition, LOC_Os04g58760, which has uncharacterized orthologs in *Arabidopsis*, maize, and sorghum, encodes a Casparian strip membrane protein domain in rice [**Supplementary Tables 6a-g**; **Supplementary Figure 5**].

**Table 2:** Potential candidate genes identified based on flanking region of QTNs and ortholog analysis.

QTN	MSU	RAP (Os ID)	Description of rice	Ortholog in *Arabidopsis*	Descriptions of *Arabidopsis*	Maize (*Zea mays* B73 v5)	Descriptions of maize	Sorghum (*Sorghum bicolor* ssp. bicolor BTx623)	Descriptions of sorghum
1-1562037	LOC_Os01g03750	Os01g0128300	Abscisic acid-deficient 4 (OsABA4)	AT1G67080	Abscisic acid (aba)-deficient 4 protein	Zm00001eb125470	Neoxanthin synthase	SORBI_3003G087100	Similar to putative uncharacterized protein P0408F06.3
1-2050328	LOC_Os01g04460	Os01g0136900	-–-	AT1G66910	Protein kinase superfamily protein	-–	-–	SORBI_3003G080900	Receptor serine/threonine kinase PR5K
2-24283728	LOC_Os02g40030	Os02g0614100	Nucleotide sugar transporter 1 (OsNST1)	AT2G14695	UDP-*N*-acetylglucosamine/UDP-glucose/GDP-mannose transporter	Zm00001eb184620	UDP-sugar transporter	SORBI_3004G214100	UDP-sugar transporter
4-34940170	LOC_Os04g58760	Os04g0684300	Casparian strip membrane domain protein 1	AT2G27370	Uncharacterized protein family	Zm00001eb065710	-–	SORBI_3006G273300	UPF0497 membrane protein Sb06g033470
6-27876287	LOC_Os06g45970/LOC_Os06g45950	Os06g0671600	Small auxin-up RNA 25 (OsSUAR25/OsSUAR26)	AT1G16510	SAUR-like auxin-responsive protein family	Zm00001eb390170	Small auxin up RNA73	SORBI_3010G224600	SAUR25-auxin-responsive SAUR family member
9-9565539	LOC_Os09g15330	Os09g0322000	Sugar transporter protein 14 (OsSTP14)	AT1G77210	Sugar transporter 14	Zm00001eb309780	-–	SORBI_3002G169300	-–
11-21301005	LOC_Os11g36440	Os11g0572700	Carotenoid isomerase (OsPHS3)	AT1G06820	Carotenoid isomerase	Zm00001eb200170	Carotenoid isomerase 1	SORBI_3005G160500	Carotenoid isomerase 1

Furthermore, these candidate gene-based haplotypes indicated that high-photosynthesis haplotypes were not confined to a single subpopulation but were contributed by different and partly overlapping subpopulations (**Supplementary Table 6f**). For example, H001 of LOC_Os01g03750 showed a high mean Pn (27.9 μmol m^−2^ s^−1^) and largely belonged to the Trp japonica and indica group, with minor contribution from the Tmp japonica group, whereas H003 of LOC_Os01g04460 had a higher mean Pn (34.38 μmol m^−2^ s^−1^), mainly contributed by the japonica group. In contrast, H006 of LOC_Os11g36440 exhibited a high mean Pn (29.9 μmol m^−2^ s^−1^) and was predominantly associated with aus, with minor representation of the indica group. Likewise, high-Pn haplotypes of other candidate genes, such as LOC_Os04g58760, LOC_Os06g45970, and LOC_Os09g15330, were largely contributed by aus, with only a limited number of accessions from the indica and admixture groups. These results suggest that favorable haplotypes arise from different subpopulations rather than from a single, common subpopulation.

**Figure 10:**
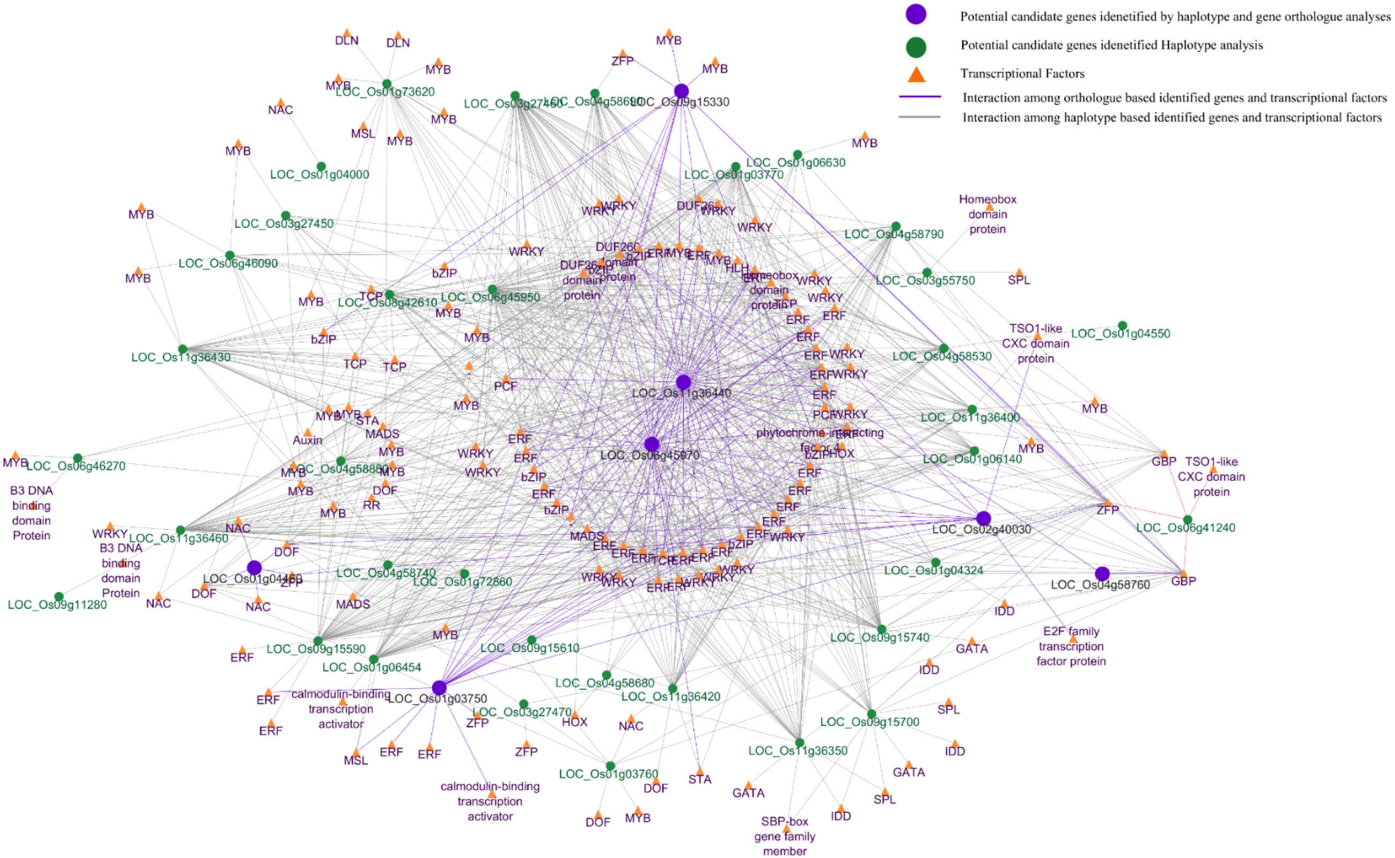
Gene network analysis of selected genes based on haplotype and ortholog analysis. A total of 43 (green circle) potential candidate genes were identified in haplotype analysis, while seven (purple circle) potential candidate genes were identified via ortholog-based analysis. Transcriptional factors (yellow triangles) were retrieved from 2-kb promoter regions. The network analysis was performed among candidate genes and transcriptional factors to highlight potential regulatory relationships and co-expression patterns.

Haplotype and ortholog analyses based on GWAS suggested potential candidate genomic regions and genes associated with Pn in rice, a complex trait that is likely involved in many interacting genes and upstream transcriptional regulators. To elucidate these regulatory interactions, *in silico* promoter motif-based analysis was performed using the 2-kb upstream regions of the 43 selected candidate genes. This analysis suggested binding motifs for several major TF families (ERF, MYB, WRKY, DOF, and MADS), and their gene annotation indicated that these TFs may highly interact with genes encoding auxin-responsive and heat shock-related proteins. Promoter regions of key candidate genes prioritized by ortholog analysis were further examined, revealing that LOC_Os04g58760, LOC_Os01g04460, LOC_Os02g40030, LOC_Os09g15330, LOC_Os01g03750, LOC_Os06g45970, and LOC_Os11g36440 contained predicted binding sites for 2, 5, 11, 30, and 41 TFs, respectively (**Figure 10**). The detailed list of transcription factors, their gene IDs, and annotations for each target gene is provided in **Supplementary Table 7**. As these TF–gene links are based on predicted cis motifs and database thresholds, further experimental validation using transcriptomic and/or TF-binding assays will be required to confirm these predicted regulatory relationships.

Haplotype analysis of these candidate genes revealed distinct patterns of genic variation in the selected five accessions, suggesting a basis for developing Kompetitive Allele Specific PCR markers for early breeding selection (**Supplementary Tables 11a-g**; **Supplementary Figure 10**). These results highlight the potential role of these selected genes in photosynthesis and related metabolic processes, which will provide a basis for functional characterization. However, detailed physiological analyses (e.g., light-response and A/Ci curves) were not performed on haplotype-based selections. Future studies incorporating contrasting haplotypes with targeted physiological measurements are needed to validate the functional effects of these loci.

### Allele profiling of QTN selection accuracy and efficiency for marker-assisted selection

To determine the practical utility of GWAS-identified QTNs in marker-assisted selection (MAS), selection accuracy and efficiency were evaluated. This analysis was used to assess how effectively each QTN could discriminate between high- and low-performing accessions for the target trait. Selection accuracy measures the proportion of individuals correctly identified as carrying the favorable allele associated with high Pn, whereas selection efficiency reflects the proportion of truly superior individuals among those selected based on the favorable allele.

Eighteen QTNs were analyzed by comparing accessions with contrasting Pn values to identify favorable alleles and estimate these two parameters. The results showed that selection accuracy and efficiency ranged 50% to 100% and 50% to 75%, with mean values of 75% and 61%, respectively (**Supplementary Figure 6**). Six QTNs—1-42455469_A_T, 9-6063219_G_A, 11-21301005_G_A, 12-11380740_C_T, 1-3000323_A_C, and 2-24283728_C_T—exhibited high accuracy (>85%) and efficiency (64%–75%), suggesting their strong potential for MAS targeting Pn improvement. Among them, 12-11380740_C_T on chromosome 12 demonstrated the highest accuracy (87%) and efficiency (72%). In contrast, three QTNs (3-15920400_C_T, 3-31894376_C_T, and 7-15847900_A_C) showed lower accuracy (<60%) but moderate efficiency (64–68%), while 8-26736462_C_T had the lowest efficiency (50%) with 60% accuracy.

Furthermore, a heat map based on photosynthetic values was constructed to visualize the distribution of favorable and unfavorable alleles among selected accessions (**Supplementary Figure 7**). The heat map revealed that accessions with high photosynthetic rates (>28 μmol m^−2^ s^−1^) carried significantly more favorable alleles than accessions with lower rates (<18 μmol m^−2^ s^−1^), suggesting that these SNPs could be valuable markers for screening high photosynthesis accessions in breeding programs. These findings also revealed that GWAS-derived SNPs provided moderate to high selection accuracy, but lower selection efficiency. The low efficiency of SNPs may be attributed to the polygenic nature of Pn, the small proportion of PVE value by each QTN, and complementary effects among the detected QTNs.

### Genomic prediction based on GWAS-derived SNPs

Genomic prediction has emerged as a transformative tool for improving polygenic traits, which are governed by numerous genes with small individual effects. The association analyses suggested that photosynthetic rate (Pn) is a polygenic trait contributing many genes. Accordingly, GP analysis was applied to assess the predictive potential of QTNs putatively associated with Pn and to evaluate model performance based on within and across populations. The relaxed thresholds in prediction-oriented frameworks were considered because of the polygenic nature of the traits, where numerous small- to moderate-effect loci collectively influence variation. For within-population prediction, six models—BA, BB, BL, gBLUP, RF, and ridge regression BLUP (rrBLUP)—were trained and tested using 1,672 SNPs (LOD > 4.0) identified using GWAS and Pn values of the association panel. The dataset was partitioned into 80% training and 20% testing subsets (**Supplementary Table 8**), and model performance was evaluated based on Pearson’s correlation coefficient (r̄_100_ ± SE) between observed and predicted Pn across 1,000 iterations. The prediction accuracy (r-value) ranged from 0.80 (RF) to 0.93 (Bayesian models and rrBLUP), indicating strong predictive capacity of GWAS-derived SNPs for Pn.

For cross-population prediction, deep learning models—convolutional neural network (CNN) and deep neural network (DNN)—were implemented using GMStool to assess model transferability across diverse genetic backgrounds. SNPs from the BLINK association model (LOD > 1.0) were selected with a fivefold CV to predict the model performance to estimate GEBV. The BLINK association model was chosen to eliminate the assumption that SNPs are evenly distributed across the genome and effectively control the population’s structure while maintaining the power to identify true genetic variants for genomic prediction across the population. The GMStool algorithms BTS and RRB identified 209 and 618 SNPs, respectively, with the highest counts on chromosome 7, followed by chromosomes 4 and 9, and the fewest on chromosomes 10, 11, and 12 (**Supplementary Figure 8**; **Supplementary Table 9**). The DNN model achieved higher prediction accuracy (r = 0.71) than the CNN (r = 0.53, **Supplementary Figure 9**).

**Figure 11:**
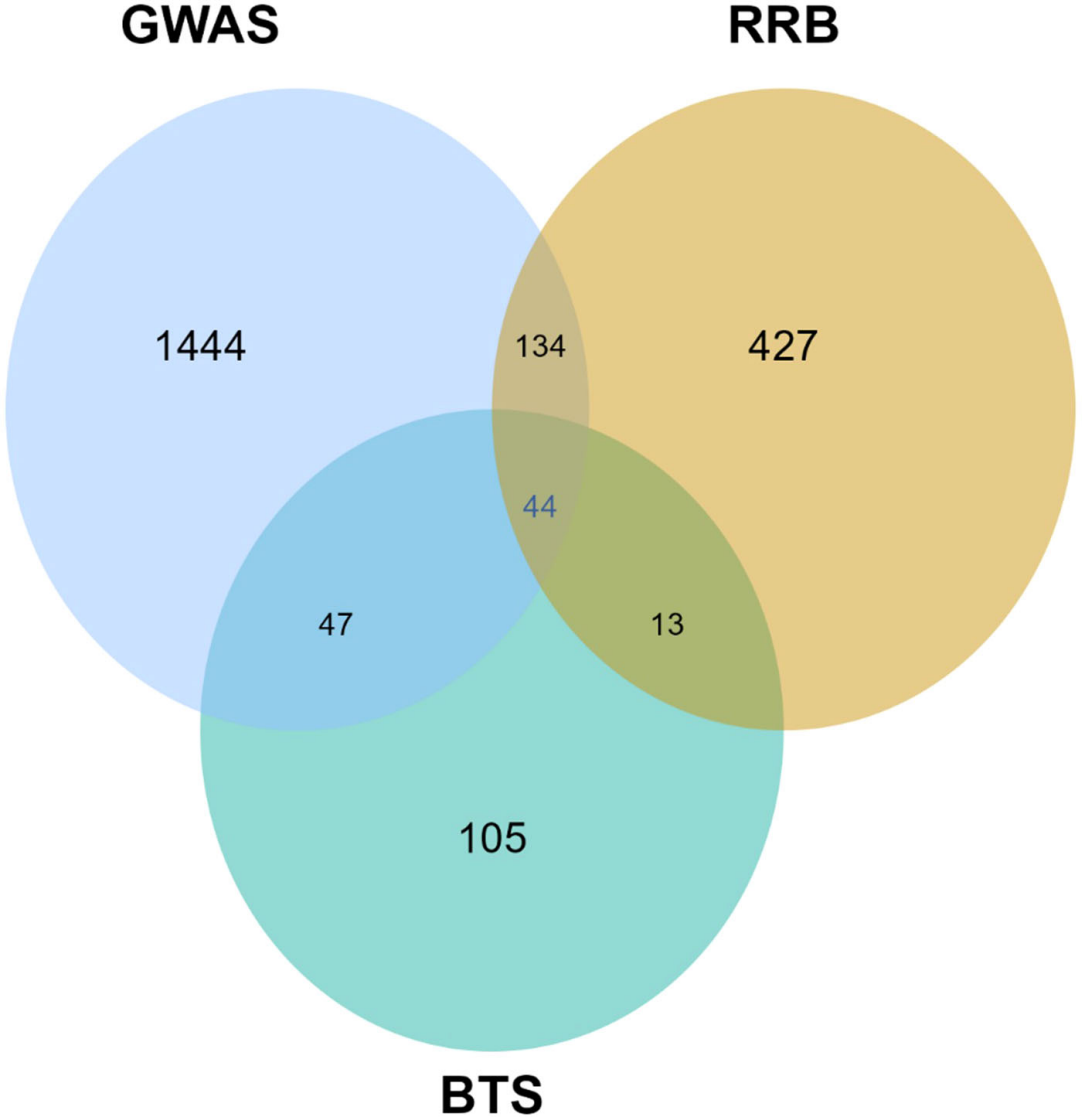
The Venn diagram illustrates the overlap of SNPs among GWAS and the genomic prediction models, BTS and RRB. A total of 44 SNPs were common across all three models. GWAS identified 1,444 unique SNPs, with 134 SNPs shared with RRB and 47 SNPs shared with BTS. The RRB model exhibited 427 unique SNPs, with 13 SNPs overlapping with BTS, while the BTS model contained 105 unique SNPs. The Venn diagram assisted in marker selection and model comparisons. GWAS, genome-wide association study.

Comparative analysis among approaches revealed an overlapping set of SNPs associated with GEBVs. A total of 134 SNPs were found common between the GWAS and RRB models, followed by 47 between the GWAS and BTS models and 13 between the RRB and BTS models. Notably, 44 SNPs were commonly detected in all three models (GWAS, BTS, and RRB), encompassing all BLINK-significant SNPs except “11-21301005” (**Figure 11**; **Supplementary Table 10**), suggesting their fundamental importance in controlling photosynthetic rate. These consensus regions, when examined within ±200-kb windows, encompassed 2,714 genes potentially associated with photosynthetic performance (**Supplementary Table 13**).

These results suggest that GWAS-derived SNPs exhibited higher correlation values compared to those selected by GMStool. This difference could potentially be attributed to overfitting models based on GMStool and tested across the population, while GMStool detected a substantially higher number of SNPs distributed across the genome. This demonstrates that the integration of both GWAS and GMS approaches offers complementary benefits for genomic prediction. While GWAS-derived markers excel in prediction accuracy within specific populations, GMStool provides broader genome coverage and potential applicability across diverse genetic backgrounds. This complementarity suggests that a combined approach may be optimal for breeding programs, where both prediction accuracy and genetic diversity are important considerations. The GWAS approach may be more suitable for immediate selection decisions within established breeding populations, while GMStool could be valuable for exploring new genetic backgrounds and maintaining broader genetic diversity in breeding programs. However, because this study was conducted in a single controlled environment, the robustness and stability of the identified QTNs and BLINK-based predictions may be uncertain under field conditions. Therefore, repeated studies across multiple locations and contrasting environments are required to ensure their reliable application in breeding programs.

## Discussion

Photosynthesis is the principal determinant of yield improvement in cereal crops, as it provides the carbohydrates necessary for grain filling and development during the reproductive stage. Numerous studies have demonstrated a strong positive correlation between crop growth rate and net CO_2_ assimilation (Murata, 1981), and a 25% increase in photosynthetic rate can result in a 22%–29% rise in biomass production (Gu et al., 2014). This strong relationship underscores the potential of improving photosynthetic efficiency as a means of enhancing crop productivity. In recent years, there has been renewed interest in optimizing photosynthetic efficiency and reallocating assimilates toward grain production to increase the harvest index and address the challenges of global food security (Long et al., 2015; Ort et al., 2015; Zhu et al., 2020).

The polygenic nature of Pn provides a rich opportunity to enhance leaf photosynthetic capacity through the exploration of natural genetic variation. Earlier studies have dissected photosynthetic mechanisms using structured populations (Adachi et al., 2011; Adachi et al., 2017; Gu et al., 2012), gene-editing approaches (Shen et al., 2019; Nayak et al., 2022), and small diversity panels (Chen et al., 2020; Lekklar et al., 2019). However, the broader genetic variation and novel favorable allelic combinations have been captured only to a limited extent in large diversity panels using advanced Pn measurement techniques in rice (Hamdani et al., 2019; Robson et al., 2023; Honda et al., 2024; Liu et al., 2024).

To address this gap, the present study employed a genetically diverse rice mini-core collection (URMC), encompassing 217 accessions that represent ~85% of global rice diversity (Agrama et al., 2010). The study combined physiological phenotyping at the reproductive stage with multi-model GWASs, haplotype and ortholog analysis, and regulatory network inference. Additionally, genomic prediction models were used to estimate GEBVs for Pn using GWAS-derived SNPs and machine learning-based feature selection. Together, these approaches enabled the identification of trait-associated putative loci, candidate genes, and predictive markers.

The phenotypic distribution of Pn observed in the URMC panel was approximately normal and ranged from 10 to 37 μmol m^−2^ s^−1^, indicating a highly polygenic control of photosynthetic rate. These results are consistent with those reported by Honda et al. (2024), Liu et al. (2024), who observed comparable variation across Tmp and Trp rice panels. The symmetric distribution of Pn values also suggested the suitability of the panel for association mapping and genomic prediction analyses. These results were consistent with previous studies that reported Pn values ranging from 21.4 to 45.5 and 11.9 to 32.1 μmol m^−2^ s^−1^ in 164 Japanese and 64 world core germplasm accessions, respectively (Honda et al., 2023; Kanemura et al., 2007).

The positive correlations among Pn, stomatal conductance, transpiration rate, and CO_2_ conductance further highlighted a coordinated regulation of carbon gain and water loss across accessions, reflecting the tight coupling between leaf hydraulics, stomatal behavior, and photosynthetic capacity in broader plant functional studies. In addition, the observed phenotypic variation in A/Q, A/Ci, and NPQ curves among the five accessions suggested adaptive potential differences in photosynthetic responses under fluctuating light and CO_2_ conditions. These physiological parameters indicated that Zhe733 had the highest photosynthetic capacity and efficient CO_2_ fixation; Cypress exhibited strong photoprotection and PSII stability; N22 displayed superior adaptation to high light and temperature with low dark respiration; GSOR310045 demonstrated high light-use efficiency; and GSOR310080 showed relatively poor photosynthetic performance in the A/Q curve but a strong response in the A/Ci curve, suggesting that this accession may perform better under elevated CO_2_ levels than under increasing light intensity. Similar findings by Kasajima et al. (2011), Weng and Chen (1987) confirm that indica accessions typically exhibit higher PSII efficiency, Pn, and lower NPQ induction than japonica types. The superior traits of Zhe733 suggested its potential as a donor line for introgression of favorable alleles into japonica backgrounds to develop high-photosynthesis cultivars.

GWASs provide a powerful framework for identifying QTLs associated with complex physiological traits such as net photosynthetic rate in diverse genetic panels (Uffelmann et al., 2021). In this study, 18 QTNs putatively associated with Pn were identified using four models—MLM, MLMM, FarmCPU, and BLINK—based on a stringent LOD score. The majority of significant QTNs were located on chromosome 1, followed by chromosomes 3, 6, and 9, suggesting that genomic regions harboring polymorphic SNPs may contribute to Pn variation in the evaluation panel. Among the identified QTNs, five major QTNs (LOD = 9–18, PVE > 10) were detected on chromosomes 1, 3, 9, and 12. Among these, only two QTN, located on chromosomes 1 and 12, were detected in different association models (BLINK and FarmCPU). Similarly, earlier studies reported that limited overlap of QTNs across different GWAS models is common for polygenic traits, as each model uses distinct strategies to account for population structure, relatedness, marker selection, and statistical power. However, given the small population size relative to marker density, the identified QTNs should be considered as preliminary candidates and require further validation in independent populations through targeted fine-mapping approaches.

The conditional PVE analysis provided important insight into the independence and joint contribution of candidate QTNs. When all putative QTNs were jointly fitted, conditional PVE values were reduced (0.17%–5.02%) relative to PVE estimates, indicating substantial sharing of phenotypic variance among loci. QTNs with low or very low PVE still explained measurable conditional PVE, suggesting that these loci contribute to Pn variation, which may be in combination with other QTNs and are likely part of a polygenic background with small but cumulative effects. QTNs exhibiting the lowest conditional PVE, despite moderate to high PVE, indicate that their apparent influence is likely attributable to linkage disequilibrium or correlation with other loci in the cumulative analysis. It may be possible that these QTNs were slightly inflated PVE due to selection bias in the GWAS analysis. In contrast, QTNs showing both high PVE and conditional PVE likely represent relatively independent loci with large contributions to photosynthesis. Collectively, these results indicate that Pn may be governed by a mixed genetic architecture in which a few large-effect QTNs act in concert with numerous loci, having minor effect.

The implementation of FarmCPU and BLINK models facilitated the minimization of confounding factors arising from population structure, kinship, and linkage disequilibrium (LD), which are frequently considered in GWASs using diverse panels. These models improved the accuracy of SNP selection and overcame the overfitting problem often encountered in traditional linear models, such as MLM and GLM, which are better suited for monogenic traits (Wang and Zhang, 2021). Previous studies have also identified genomic regions associated with Pn using various models. For example, GLM detected genomic region on chromosomes 4 and 8 for photosynthesis (Honda et al., 2023); LMM revealed loci on chromosomes 1 and 12 for Fv/Fm (Hamdani et al., 2019); MLMM identified genomic region on chromosomes 2, 3, 5, 6, 7, and 12 for PSII efficiency (Liu et al., 2023); and EMMAX, LFMM, and FarmCPU uncovered loci on chromosomes 2, 3, 5, 7, 8, 9, 10, and 11 for chlorophyll fluorescence responses to temperature (Robson et al., 2023). Likewise, MLM identified associations on chromosomes 2, 5, and 7 for NPQ (Wei et al., 2022). Our results are broadly consistent with these reports and suggest that Pn is governed by a mixed genetic architecture involving loci of varying effect sizes.

To explore functional relevance, 1,091 coding and non-coding genes were identified within ±200-kb windows around each QTN, capturing candidate genes likely in LD with the associations (**Supplementary Table 2**). The candidate genes were functional categories in three class based on annotation: 1) core photosynthetic machinery genes were identified on chromosomes 3 and 12, including components like RPE, Rubisco, ATP synthase, and cytochromes, which contribute in carbon fixation and energy conversion processes; 2) regulatory genes comprising various transcription factors (MYB, bHLH, NAM, and AP2-like) and signaling components (kinases, phosphatases, and hormone-related genes) that control gene expression and cellular responses. These regulatory genes were dispersed in locality of putative genomic region except those located on chromosomes 3 and 12; 3) cellular homeostasis and heat (produced during high light intensity) responsible genes, including antioxidant enzymes, heat shock proteins, protease inhibitors, and transporters, which may be involved in cellular stability under high light intensity or other environmental conditions, were dispersed on genomic regions located on chromosomes 1, 6, 8, and 9.

The 200-kb LD window was selected as a conservative approach across the six rice subpopulations, which show heterogeneous LD decay from ~75 to >500 kb. This choice reduces the risk of missing true candidate genes but may still underestimate candidate genes in subpopulations with very slow LD decay (for example, Tmp japonica with LD > 500 kb) and, conversely, may include non-causal candidate genes in subpopulations with faster decay (for example, indica and Trp japonica with LD ~75 and >150 kb, respectively). Therefore, future analyses should estimate LD decay separately for each subpopulation and apply subpopulation-specific, variable genomic windows around QTNs, which would improve mapping resolution and more precisely define candidate gene intervals. Subsequently, haplotype analysis of QTN-flanking genes (≥5 accessions per haplotype) highlighted 43 candidate genes across 13 putative QTNs (**Supplementary Table 3**). To further refine functional relevance, ortholog-based comparative analysis in *Arabidopsis* and gene regulatory analyses revealed that 19 out of 43 candidate genes showed 50 orthologs involved in signaling, transport, and cellular homeostasis (**Supplementary Table 4**), while the remaining 24 candidate genes had no ortholog compared to *Arabidopsis*. Seven candidate genes were prioritized based on their ortholog found in *Arabidopsis*, maize, and sorghum and their contributions in diverse processes crucial for photosynthesis, including carotenoid synthesis, sugar transport, protein kinase activity, and Casparian strip membrane protein formation. Among these prioritized candidate genes, LOC_Os01g03750 (OsABA4) is located within the 8-kb region of putative QTN “1-1562037”, which potentially involves ABA biosynthesis and PSII photoprotection pathways. Previous studies have reported that MHZ4/OsABA4 is a chloroplast-localized protein for ABA production and have suggested that it is involved in sugar signaling, ethylene–ABA cross-talk, and grain filling processes (Liu et al., 2021; Rook et al., 2001; Yang et al., 2006). Similarly, LOC_Os11g36440 (CRTISO) was identified as a putative candidate gene associated with the moderate-effect QTN 11-21301005. CRTISO is known to function within the carotenoid pathway to support chlorophyll–protein complexes and NPQ-based photoprotection. Its proximity to this QTN suggests a potential role in maintaining PSII stability and mediating the photosynthetic variations observed among the accessions (Fang et al., 2008; Niyogi, 1999; Wei et al., 2010). Additionally, two transport-related candidate genes were identified—LOC_Os02g40030 (nucleotide sugar transporter), in the flanking region of putative QTN “2-24283728” and LOC_Os09g15330 (monosaccharide transporter protein)—linked with one putative QTN “9-9565539” (LOD = 18.28, PVE = 17.46%). These genes correspond to AT2G14695 (GDP-mannose transporter) and AT1G77210 (sugar transporter 14) homologs, respectively, potentially supporting carbohydrate flux, cell-wall biosynthesis, and stress responses (Song et al., 2011; Zhang et al., 2011; Deng et al., 2019; Ren et al., 2020).

LOC_Os06g45970 (an early auxin-responsive gene) and LOC_Os01g04460 (a protein kinase) were identified in the flanking region of QTN 6-27876287 and QTN 1-2050328, respectively. These putative candidates are homologous to At1g16510 and At1g66910, suggesting that auxin signaling and phosphorylation pathways may contribute to the regulation of rice growth and photosynthesis (Jain et al., 2006). Furthermore, LOC_Os04g58760 (Casparian strip membrane domain protein), homologous to *A*t2g27370, was identified within the flanking region of the moderate-effect QTN 4-34940170. This putative candidate is known to participate in root endodermal barrier formation and nutrient homeostasis, suggesting a potential indirect link between root-mediated nutrient uptake and photosynthetic efficiency (Wang et al., 2019).

Expression analysis using public databases (RiceXPro: https://ricexpro.dna.affrc.go.jp/) indicated that these candidate genes are highly expressed in rice leaves during the reproductive stage. This expression pattern is consistent with their putative roles in supporting photosynthesis, carbon assimilation, and the increased energy demand during reproductive development. However, given that photosynthesis is highly sensitive to environmental conditions and shows strong temporal (diurnal) variation, further validation will be required using haplotype-based expression analyses under well-defined controlled conditions and across different times of the day, and by systematically varying key environmental factors such as light intensity and temperature, to determine how allelic variation influences gene expression responses and photosynthetic performance.

Additionally, promoter (2-kb) motif analysis across the 43 candidate genes revealed enrichment for ERF, MYB, WRKY, DOF, and MADS TF families, with dense TF–target connectivity with the seven prioritized candidate genes; for example, LOC_Os11g36440 was associated with 41 putative TF interactions. Haplotype analysis across the five phenotype accessions showed distinct groupings between high and low classes, providing a preliminary basis for KASP assay design. Furthermore, previous studies have indicated that the chloroplast genome, together with nuclear genes, can influence photosynthetic traits through cytonuclear interactions (Dubreuil et al., 2018; Lee et al., 2021). In this study, only nuclear SNP variation was analyzed using association analysis, so potential cytoplasmic effects on Pn could not be evaluated, which represents a limitation of this study. Marker-assisted breeding offers significant advantages over conventional breeding methods for the improvement of plant major traits. In this study, we estimated selection accuracy and efficiency to identify the potential favorable versus unfavorable alleles from GWAS-derived putative QTN regions that contribute to Pn. Our analysis identified six promising SNPs, including two consistent SNPs with high selection accuracy (>85%) and efficiency (64% to 75%) as a putative target for KAPS assay development. However, the practical application of these identified SNPs in rice breeding programs necessitates the development and validation of functional KASP marker assays, which extend beyond the scope of the current study.

GP and genomic selection (GS) enable the selection of individuals based on genomic performance without direct phenotypic evaluation (Alemu et al., 2024). In this study, we evaluated GP models for Pn using two approaches: within-population validation with strongly linked SNPs (LOD > 4) and cross-population validation using a broader SNP set (LOD > 1) from the BLINK association model. The first approach showed high accuracy (r = 0.82–0.92) across all models, likely due to the use of highly associated SNPs validated within the same population (Gu et al., 2014; Rios et al., 2021; Guo et al., 2014). In contrast, the neural network model showed lower accuracy (r = 0.56–0.72) in cross-population validation, attributed to the larger, less stringent SNP dataset and validation across different populations. Despite lower accuracy, the cross-population approach provides a more realistic assessment across diverse genetic backgrounds (Yu et al., 2024; Brault et al., 2022). Through the integration of GWAS and neural network approaches, we identified 44 candidate SNPs, with gene mining revealing 2,715 putative candidate genes with functions potentially linked to sucrose and protein transportation, Calvin cycle, growth regulation, and stress responses. Notably, this final set included all 18 putative QTNs, suggesting that this integrated approach effectively captures genetic variation across populations. These findings provide a preliminary genomic toolkit for rice improvement, with the identified loci representing high-priority targets for future functional validation and marker-assisted breeding.

As a next step, these candidate regions could be refined using subpopulation-specific LD decay together with gene expression and network profiling across key tissues and developmental stages to more precisely pinpoint causal genes. In addition, validating the effects of prioritized candidate genes across multiple locations and years under field conditions will be essential to confirm their robustness and to translate this genomic toolkit into breeding strategies for developing high-Pn, climate-resilient rice cultivars.

## Conclusion

This comprehensive study explored the genetic architecture of the rice diversity panel for Pn under controlled conditions at the booting stage. Population structure and PCA identified six distinct subpopulations explaining the broad range of Pn variation. Based on association studies and allelic profiling, we suggested 18 putative QTNs and their favorable alleles, which may improve selection accuracy for marker-assisted selection for cultivar development.

Subsequently, gene mining, haplotype, ortholog, and regulatory gene network analysis suggested seven candidate genes with their haplotypes located in the flanking region of putative QTNs that are associated with photosynthesis-related processes. These candidates provide promising targets for further validation and functional study. To enhance breeding efficiency, genomic predictions using multiple models were evaluated within and across populations, and a set of 44 candidate SNPs potentially contributing to genomic estimated breeding value was identified to improve genetic gain over the breeding pipeline.

Physiological analyses, including chlorophyll fluorescence, light curve, and CO_2_ response curve of selected accessions, suggested that Zhe733 displayed greater adaptability to environmental fluctuations compared to those of japonica background accessions, potentially reflecting broader genetic diversity and enhanced photosynthetic plasticity.

Overall, this study provides valuable insights into the associated genetic architecture of photosynthesis within the URMC diversity panel and suggests a set of KASP assays that may assist in selecting promising donor parents in breeding programs aimed at developing Pn-efficient rice cultivars. Future research should focus on validating these associations through fine mapping and functional characterization to better understand their contribution to phenotypic plasticity and yield potential.

## Data Availability

The datasets presented in this study can be found in online repositories. The names of the repository/repositories and accession number(s) can be found in the article/**Supplementary Material**.

## References

[B1] Acevedo-Siaca L. G. Coe R. Wang Y. Kromdijk J. Quick W. P. Long S. P. (2020). Variation in photosynthetic induction between rice accessions and its potential for improving productivity. New Phytol. 227, 1097–1108. doi: 10.1111/nph.16454, PMID: 32124982 PMC7383871

[B2] Adachi S. Nito N. Kondo M. Yamamoto T. Arai-Sanoh Y. Ando T. . (2011). Identification of chromosomal regions controlling the leaf photosynthetic rate in rice by using a progeny from japonica and high-yielding *indica* varieties. Plant Prod. Sci. 14, 118–127. doi: 10.1626/pps.14.118

[B3] Adachi S. Yoshikawa K. Yamanouchi U. Tanabata T. Sun J. Ookawa T. . (2017). Fine mapping of *carbon assimilation rate* 8, a quantitative trait locus for flag leaf nitrogen content, stomatal conductance and photosynthesis in rice. Front. Plant Sci. 8. doi: 10.3389/fpls.2017.00060, PMID: 28197156 PMC5282472

[B4] Agrama H. A. Yan W. Jia M. Fjellstrom R. McClung A. M. (2010). Genetic structure associated with diversity and geographic distribution in the USDA rice world collection: Natural Science. 2, 247–291. doi: 10.4236/ns.2010.24036

[B5] Alemu A. Åstrand J. Montesinos-López O. A. Sánchez J. I. Y. Fernández-González J. Tadesse W. . (2024). Genomic selection in plant breeding: Key factors shaping two decades of progress. Mol. Plant 17, 552–578. doi: 10.1016/j.molp.2024.03.007, PMID: 38475993

[B6] Alexander D. H. Novembre J. Lange K. (2009). Fast model-based estimation of ancestry in unrelated individuals. Genome Res. 19, 1655–1664. doi: 10.1101/gr.094052.109, PMID: 19648217 PMC2752134

[B7] Ambavaram M. M. R. Basu S. Krishnan A. Ramegowda V. Batlang U. Rahman L. . (2014). Coordinated regulation of photosynthesis in rice increases yield and tolerance to environmental stress. Nat. Commun. 5, 5302. doi: 10.1038/ncomms6302, PMID: 25358745 PMC4220491

[B8] Baly E. C. C. (1935). The kinetics of photosynthesis. Proc. R. Soc. Lond. 17, 218–239. doi: 10.1098/rspb.1935.0026, PMID: 34341189

[B9] Bartholomé J. Frouin J. Brottier L. Cao T. V. Boisnard A. Ahmadi N. . (2023). Genomic selection for salinity tolerance in japonica rice. PloS One 18, e0291833. doi: 10.1371/journal.pone.0291833, PMID: 37756295 PMC10530037

[B10] Bassman J. H. Zwier J. C. (1991). Gas-exchange characteristics of populus-trichocarpa, populus-deltoides and populus-trichocarpa x populus-deltoides clones. Tree Physiol. 8, 145–159. doi: 10.1093/treephys/8.2.145, PMID: 14972886

[B11] BD Van Der Auwera G. A. O’Connor B. (2020). Genomics in the cloud: using docker, GATK, and WDL in terra. 1st Edition edn (Sebastopol, CA: O’Reilly Media).

[B12] Brault C. Segura V. This P. Le Cunff L. Flutre T. François P. . (2022). Across-population genomic prediction in grapevine opens up promising prospects for breeding. Hortic. Res. 9, uhac041. doi: 10.1093/hr/uhac041, PMID: 35184162 PMC9070645

[B13] Breiman L. (2001). Random forests. Mach. Learn. 45, 5–32. doi: 10.1023/A:1010933404324, PMID: 41886696

[B14] Chen J. N. Cao F. B. Li H. L. Shan S. L. Tao Z. Lei T. . (2020). Genotypic variation in the grain photosynthetic contribution to grain filling in rice. J. Plant Physiol. 253, 153269. doi: 10.1016/j.jplph.2020.153269, PMID: 32906075

[B15] Deng X. L. An B. G. Zhong H. Yang J. Kong W. L. Li Y. S. (2019). A novel insight into functional divergence of the MST gene family in rice based on comprehensive expression patterns. Genes 10, 239. doi: 10.3390/genes10030239, PMID: 30897847 PMC6470851

[B16] Doncheva N. T. Morris J. H. Gorodkin J. Jensen L. J. (2018). Cytoscape stringApp: network analysis and visualization of proteomics data. J. Proteome Res. 18, 623–632. doi: 10.1021/acs.jproteome.8b00702, PMID: 30450911 PMC6800166

[B17] Dubreuil C. Jin X. Barajas-López J. D. Hewitt T. C. Tanz S. K. Dobrenel T. . (2018). Establishment of photosynthesis through chloroplast development is controlled by two distinct regulatory phases. Plant Physiol. 176, 1199–1214. doi: 10.1104/pp.17.00435, PMID: 28626007 PMC5813571

[B18] Fang J. Chai C. L. Qian Q. Li C. L. Tang J. Y. Sun L. . (2008). Mutations of genes in synthesis of the carotenoid precursors of ABA lead to pre-harvest sprouting and photo-oxidation in rice. Plant J. 54, 177–189. doi: 10.1111/j.1365-313X.2008.03411.x, PMID: 18208525 PMC2327239

[B19] Farquhar G. D. Caemmerer S. V. Berry J. A. (1980). A biochemical-model of photosynthetic co2 assimilation in leaves of c-3 species. Planta 149, 78–90. doi: 10.1007/BF00386231, PMID: 24306196

[B20] Gaur V. S. Channappa G. Chakraborti M. Sharma T. R. Mondal T. K. (2020). ‘Green revolution’ dwarf gene sd1 of rice has gigantic impact. Briefings Funct. Genomics 19, 390–409. doi: 10.1093/bfgp/elaa019

[B21] Gu J. F. Yin X. Y. Stomph T. J. Struik P. C. (2014). Can exploiting natural genetic variation in leaf photosynthesis contribute to increasing rice productivity? A simulation analysis. Plant Cell Environ. 37, 22–34. doi: 10.1111/pce.12173, PMID: 23937619

[B22] Gu J. F. Yin X. Y. Struik P. C. Stomph T. J. Wang H. Q. (2012). Using chromosome introgression lines to map quantitative trait loci for photosynthesis parameters in rice (*Oryza sativa* L.) leaves under drought and well-watered field conditions. J. Exp. Bot. 63, 455–469. doi: 10.1093/jxb/err292, PMID: 21984650 PMC3245479

[B23] Guo Z. Tucker D. M. Basten C. J. Gandhi H. Ersoz E. Guo B. . (2014). The impact of population structure on genomic prediction in stratified populations. Theor. Appl. Genet. 127, 749–762. doi: 10.1007/s00122-013-2255-x, PMID: 24452438

[B24] Gutteridge S. (2018). The impact of a changing atmosphere on chloroplast function, photosynthesis, yield, and food security. Chloropl.: Cap. Prod. Mod. Plants 62, 1–11. doi: 10.1042/EBC20180023, PMID: 29653966

[B25] Hamdani S. Wang H. R. Zheng G. Y. Perveen S. Qu M. N. Khan N. . (2019). Genome-wide association study identifies variation of glucosidase being linked to natural variation of the maximal quantum yield of photosystem II. Physiol. Plant. 166, 105–119. doi: 10.1111/ppl.12957, PMID: 30834537

[B26] Hamilton J. P. Li C. X. Buell C. R. (2024). The rice genome annotation project: an updated database for mining the rice genome. Nucleic Acids Res. 53, D1614–D1622. doi: 10.1093/nar/gkae1061, PMID: 39558187 PMC11701632

[B27] Hedden P. (2003). The genes of the green revolution. Trends Genet. 19, 5–9. doi: 10.1016/S0168-9525(02)00009-4, PMID: 12493241

[B28] Honda S. Imamura A. Seki Y. Chigira K. Iwasa M. Hayami K. . (2024). Genome-wide association study of leaf photosynthesis using a high-throughput gas exchange system in rice. Photosyn. Res. 159, 17–28. doi: 10.1007/s11120-023-01065-3, PMID: 38112862

[B29] Hubbart S. Peng S. Horton P. Chen Y. Murchie E. H. (2007). Trends in leaf photosynthesis in historical rice varieties developed in the Philippines since 1966. J. Exp. Bot. 58, 3429–3438. doi: 10.1093/jxb/erm192, PMID: 17875814

[B30] Jain M. Tyagi A. K. Khurana J. P. (2006). Genome-wide analysis, evolutionary expansion, and expression of early auxin-responsive SAUR gene family in rice (*Oryza sativa*). Genomics 88, 360–371. doi: 10.1016/j.ygeno.2006.04.008, PMID: 16707243

[B31] Jeong S. Kim J. Y. Kim N. (2020). GMStool: GWAS-based marker selection tool for genomic prediction from genomic data. Sci. Rep. 10, 19653. doi: 10.1038/s41598-020-76759-y, PMID: 33184432 PMC7665227

[B32] Kanemura T. Homma K. Ohsumi A. Shiraiwa T. Horie T. (2007). Evaluation of genotypic variation in leaf photosynthetic rate and its associated factors by using rice diversity research set of germplasm. Photosyn. Res. 94, 23–30. doi: 10.1007/s11120-007-9208-7, PMID: 17659450

[B33] Karatzoglou A. Smola A. Hornik K. Zeileis A. (2004). Kernlab-an S4 package for kernel methods in R. Journal of statistical software 11, 1–20.

[B34] Kasajima I. Ebana K. Yamamoto T. Takahara K. Yano M. Kawai-Yamada M. . (2011). Molecular distinction in genetic regulation of nonphotochemical quenching in rice. Proc. Natl. Acad. Sci. Utd. States America 108, 13835–13840. doi: 10.1073/pnas.1104809108, PMID: 21804028 PMC3158180

[B35] Kawahara Y. de la Bastide M. Hamilton J. P. Kanamori H. McCombie W. R. Ouyang S. . (2013). Improvement of the Oryza sativa Nipponbare reference genome using next generation sequence and optical map data. Rice 6, 4. doi: 10.1186/1939-8433-6-4, PMID: 24280374 PMC5395016

[B36] Kinsella R. J. Kähäri A. Haider S. Zamora J. Proctor G. Spudich G. . (2011). Ensembl BioMarts: a hub for data retrieval across taxonomic space Database. J. Biol. Database Curation. 2011, bar030. doi: 10.1093/database/bar030, PMID: 21785142 PMC3170168

[B37] Korte A. Farlow A. (2013). The advantages and limitations of trait analysis with GWAS: a review. Plant Methods 9, 29. doi: 10.1186/1746-4811-9-29, PMID: 23876160 PMC3750305

[B38] Kumar A. Gupta C. Thomas J. Pereira A. (2021). Genetic dissection of grain yield component traits under high nighttime temperature stress in a rice diversity panel. Front. Plant Sci. 12. doi: 10.3389/fpls.2021.712167, PMID: 34650575 PMC8508263

[B39] Lee D.-Y. Hua L. Khoshravesh R. Giuliani R. Kumar I. Cousins A. . (2021). Engineering chloroplast development in rice through cell-specific control of endogenous genetic circuits. Plant Biotechnol. J. 19, 2291–2303. doi: 10.1111/pbi.13660, PMID: 34328250 PMC8541780

[B40] Lekklar C. Suriya-arunroj D. Pongpanich M. Comai L. Kositsup B. Chadchawan S. . (2019). Comparative genomic analysis of rice with contrasting photosynthesis and grain production under salt stress. Genes 10, 562. doi: 10.3390/genes10080562, PMID: 31349693 PMC6722916

[B41] Li P. P. Jiang J. Zhang G. G. Miao S. Y. Lu J. B. Qian Y. K. . (2023). Integrating GWAS and transcriptomics to identify candidate genes conferring heat tolerance in rice. Front. Plant Sci. 13. doi: 10.3389/fpls.2022.1102938, PMID: 36699845 PMC9868562

[B42] Li X. B. Yan W. G. Agrama H. Hu B. L. Jia L. M. Jia M. L. . (2010). Genotypic and phenotypic characterization of genetic differentiation and diversity in the USDA rice mini-core collection. Genetica 138, 1221–1230. doi: 10.1007/s10709-010-9521-5, PMID: 21080033

[B43] Liu Q. Dong G. R. Ma Y. Q. Zhao S. M. Liu X. Li X. K. . (2021). Rice glycosyltransferase gene *UGT85E1* is involved in drought stress tolerance through enhancing abscisic acid response. Front. Plant Sci. 12. doi: 10.3389/fpls.2021.790195, PMID: 35003178 PMC8733621

[B44] Liu S. C. Xiong Z. Zhang Z. L. Wei Y. B. Xiong D. L. Wang F. . (2023). Exploration of chlorophyll fluorescence characteristics gene regulatory in rice (*Oryza sativa* L.): a genome-wide association study. Front. Plant Sci. 14. doi: 10.3389/fpls.2023.1234866, PMID: 37746023 PMC10513790

[B45] Liu S. S. Xu Z. Essemine J. Liu Y. M. Liu C. D. Zhang F. X. . (2024). GWAS unravels acid phosphatase ACP2 as a photosynthesis regulator under phosphate starvation conditions through modulating serine metabolism in rice. Plant Commun. 5. doi: 10.1016/j.xplc.2024.100885, PMID: 38504521 PMC11287135

[B46] Long S. P. Zhu X. G. Naidu S. L. Ort D. R. (2006). Can improvement in photosynthesis increase crop yields? Plant Cell Environ. 29, 315–330. doi: 10.1111/j.1365-3040.2005.01493.x, PMID: 17080588

[B47] Long S. P. Marshall-Colon A. Zhu X. G. (2015). Meeting the global food demand of the future by engineering crop photosynthesis and yield potential. Cell 161 (1), 56–66. doi: 10.1016/j.cell.2015.03.019, PMID: 25815985

[B48] Mather K. A. Caicedo A. L. Polato N. R. Olsen K. M. McCouch S. Purugganan M. D. (2007). The extent of linkage disequilibrium in rice (Oryza sativa L.). Genetics. 177, 2223–2232. doi: 10.1534/genetics.107.079616, PMID: 17947413 PMC2219496

[B49] Mohanty S. (2013). Trends in global rice consumption. Rice Today 12, 44–45.

[B50] Murata Y. (1981). Dependence of potential productivity and efficiency for solar-energy utilization on leaf photosynthetic capacity in crop species. Japan. J. Crop Sci. 50, 223–232. doi: 10.1626/jcs.50.223

[B51] Nayak L. Panda D. Dash G. K. Lal M. K. Swain P. Baig M. J. . (2022). A chloroplast Glycolate catabolic pathway bypassing the endogenous photorespiratory cycle enhances photosynthesis, biomass and yield in rice (Oryza sativa L.). Plant Sci. 314, 111103. doi: 10.1016/j.plantsci.2021.111103, PMID: 34895540

[B52] Nguyen V. Morantte R. I. Z. Lopena V. Verdeprado H. Murori R. Ndayiragije A. . (2023). Multi-environment genomic selection in rice elite breeding lines. Rice 16, 7. doi: 10.1186/s12284-023-00623-6, PMID: 36752880 PMC9908796

[B53] Nicol L. Nawrocki W. J. Croce R. (2019). Disentangling the sites of non-photochemical quenching in vascular plants. Nat. Plants 5, 1177–1183. doi: 10.1038/s41477-019-0526-5, PMID: 31659240 PMC6861128

[B54] Niyogi K. K. (1999). Photoprotection revisited: Genetic and molecular approaches. Annu. Rev. Plant Physiol. Plant Mol. Biol. 50, 333–359. doi: 10.1146/annurev.arplant.50.1.333, PMID: 15012213

[B55] Ogren E. Evans J. R. (1993). Photosynthetic light-response curves.1. the influence of co2 partial-pressure and leaf inversion. Planta 189, 182–190. doi: 10.1007/BF00195075, PMID: 30311153

[B56] Ort D. R. Merchant S. S. Alric J. Barkan A. Blankenship R. E. Bock R. Croce R. . (2015). Redesigning photosynthesis to sustainably meet global food and bioenergy demand. Proc. Natl. Acad. Sci. U.S.A. 112 (28), 8529–8536. doi: 10.1073/pnas.1424031112, PMID: 26124102 PMC4507207

[B57] Pérez-Rodríguez P. De Los Campos G. (2022). Multitrait Bayesian shrinkage and variable selection models with the BGLR-R package. Genetics 222, iyac112. doi: 10.1093/genetics/iyac112, PMID: 35924977 PMC9434216

[B58] Posada J. M. Lechowicz M. J. Kitajima K. (2009). Optimal photosynthetic use of light by tropical tree crowns achieved by adjustment of individual leaf angles and nitrogen content. Ann. Bot. 103, 795–805. doi: 10.1093/aob/mcn265, PMID: 19151040 PMC2707872

[B59] Ralph P. J. Gademann R. (2005). Rapid light curves: A powerful tool to assess photosynthetic activity. Aquat. Bot. 82, 222–237. doi: 10.1016/j.aquabot.2005.02.006, PMID: 38826717

[B60] Ravelombola W. S. Qin J. Shi A. Nice L. Bao Y. Lorenz A. . (2019). Genome-wide association study and genomic selection for soybean chlorophyll content associated with soybean cyst nematode tolerance. BMC Genomics 20, 904. doi: 10.1186/s12864-019-6275-z, PMID: 31775625 PMC6882315

[B61] Ren L. J. Zhao T. T. Zhang L. Du G. J. Shen Y. Tang D. . (2020). *Defective Microspore Development* 1 is required for microspore cell integrity and pollen wall formation in rice. Plant J. 103, 1446–1459. doi: 10.1111/tpj.14811, PMID: 32391618

[B62] Riaz A. Raza Q. Kumar A. Dean D. Chiwina K. Phiri T. M. . (2023). GWAS and genomic selection for marker-assisted development of sucrose enriched soybean cultivars. Euphytica 219, 97. doi: 10.1007/s10681-023-03224-y, PMID: 30311153

[B63] Rios E. F. Andrade M. H. M. L. Resende M. F. R. Kirst M. de Resende M. D. V. de Almeida Filho J. E. . (2021). Genomic prediction in family bulks using different traits and cross-validations in pine. G3 (Bethesda). 11, jkab249. doi: 10.1093/g3journal/jkab249, PMID: 34544139 PMC8496210

[B64] Robson J. K. Ferguson J. N. McAusland L. Atkinson J. A. Tranchant-Dubreuil C. Cubry P. . (2023). Chlorophyll fluorescence-based high-throughput phenotyping facilitates the genetic dissection of photosynthetic heat tolerance in African (Oryza glaberrima) and Asian (Oryza sativa) rice. J. Exp. Bot. 74, 5181–5197. doi: 10.1093/jxb/erad239, PMID: 37347829 PMC10498015

[B65] Rook F. Corke F. Card R. Munz G. Smith C. Bevan M. W. (2001). Impaired sucrose-induction mutants reveal the modulation of sugar-induced starch biosynthetic gene expression by abscisic acid signalling. Plant J. 26, 421–433. doi: 10.1046/j.1365-313X.2001.2641043.x, PMID: 11439129

[B66] Sasaki H. Ishii R. (1992). Cultivar differences in leaf photosynthesis of rice bred in Japan. Photosyn. Res. 32, 139–146. doi: 10.1007/BF00035948, PMID: 24408284

[B67] Schreiber U. Bilger W. Neubauer C. (1995). Chlorophyll fluorescence as a nonintrusive indicator for rapid assessment of in vivo photosynthesis. Schulze E. D. Caldwell M. M. . Ecophysiology of photosynthesis. Berlin, Heidelberg: Springer, 100. doi: 10.1007/978-3-642-79354-7_3, PMID:

[B68] Sharkey T. D. Bernacchi C. J. Farquhar G. D. Singsaas E. L. (2007). Fitting photosynthetic carbon dioxide response curves for C3 leaves. Plant, cell & environment, 30 (9), pp.1035–1040. 10.1111/j.1365-3040.2007.01710.x 17661745

[B69] Shen B. R. Wang L. M. Lin X. L. Yao Z. Xu H. W. Zhu C. H. . (2019). Engineering a new chloroplastic photorespiratory bypass to increase photosynthetic efficiency and productivity in rice. Mol. Plant 12, 199–214. doi: 10.1016/j.molp.2018.11.013, PMID: 30639120

[B70] Sherman B. T. Hao M. Qiu J. Jiao X. L. Baseler M. W. Lane H. C. . (2022). DDAVID: a web server for functional enrichment analysis and functional annotation of gene lists, (2021 update). Nucleic Acids Res. 50, W216–W221. doi: 10.1093/nar/gkac194, PMID: 35325185 PMC9252805

[B71] Shi A. Mou B. Correll J. Motes D. Weng Y. Qin J. (2016). SNP association analysis of resistance to Verticillium wilt (Verticillium dahliae Kleb.) in spinach. Aust. J. Crop Sci. 10, 8, 1188–1196.

[B72] Smith Emil L. (1936). Photosynthesis in relation to light and carbon dioxide. 22, 504–511. doi: 10.1073/pnas.22.8.504, PMID: 16577734 PMC1079215

[B73] Song X. Zhang B. Zhou Y. (2011). Golgi-localized UDP-glucose transporter is required for cell wall integrity in rice. Plant Signaling Behav. 6, 1097–1100. doi: 10.4161/psb.6.8.16379, PMID: 21822061 PMC3260701

[B74] Spindel J. Begum H. Akdemir D. Virk P. Collard B. Redoña E. . (2015). Genomic selection and association mapping in rice (*Oryza sativa*): effect of trait genetic architecture, training population composition, marker number and statistical model on accuracy of rice genomic selection in elite, tropical rice breeding lines. PloS Genet. 11, e1004982. doi: 10.1371/journal.pgen.1004982, PMID: 25689273 PMC4334555

[B75] Su J. J. Pang C. Y. Wei H. L. Li L. B. Liang B. Wang C. X. . (2016). Identification of favorable SNP alleles and candidate genes for traits related to early maturity via GWAS in upland cotton. BMC Genomics 17, 687. doi: 10.1186/s12864-016-2875-z, PMID: 27576450 PMC5006539

[B76] Takai T. Adachi S. Taguchi-Shiobara F. Sanoh-Arai Y. Iwasawa N. Yoshinaga S. . (2013). A natural variant of NAL1, selected in high-yield rice breeding programs, pleiotropically increases photosynthesis rate. Sci. Rep. 3, 2149. doi: 10.1038/srep02149, PMID: 23985993 PMC3756344

[B77] Takai T. Kondo M. Yano M. Yamamoto T. (2010). A quantitative trait locus for chlorophyll content and its association with leaf photosynthesis in rice. Rice 3, 172–180. doi: 10.1007/s12284-010-9047-6, PMID: 30311153

[B78] Tian F. Yang D. C. Meng Y. Q. Jin J. P. Gao G. (2020). PlantRegMap: charting functional regulatory maps in plants. Nucleic Acids Res. 48, D1104–D1113. doi: 10.1093/nar/gkz1020, PMID: 31701126 PMC7145545

[B79] Uffelmann E. Huang Q. Q. Munung N. S. de Vries J. Okada Y. Martin A. R. . (2021). Genome-wide association studies. Nat. Rev. Methods Primers 1, 59. doi: 10.1038/s43586-021-00056-9, PMID: 37880705

[B80] Von Caemmerer S. (2000). Biochemical models of leaf photosynthesis ( Csiro Publishing). doi: 10.1071/9780643103405, PMID:

[B81] Wang C. Guo L. Li Y. Wang Z. (2012). Systematic comparison of C3 and C4 plants based on metabolic network analysis. BMC Syst. Biol. 6, S9. doi: 10.1186/1752-0509-6-S2-S9, PMID: 23281598 PMC3521184

[B82] Wang Q. Tang J. L. Han B. Huang X. H. (2020). Advances in genome-wide association studies of complex traits in rice. Theor. Appl. Genet. 133, 1415–1425. doi: 10.1007/s00122-019-03473-3, PMID: 31720701

[B83] Wang Z. G. Yamaji N. K. Huang S. Zhang X. Shi M. X. Fu S. . (2019). OsCASP1 is required for casparian strip formation at endodermal cells of rice roots for selective uptake of mineral elements. Plant Cell 31, 2636–2648. doi: 10.1105/tpc.19.00296, PMID: 31484684 PMC6881135

[B84] Wang J. B. Zhang Z. W. (2021). GAPIT version 3: boosting power and accuracy for genomic association and prediction. Genomics Proteomics Bioinf. 19, 629–640. doi: 10.1016/j.gpb.2021.08.005, PMID: 34492338 PMC9121400

[B85] Wei Y. B. Liu S. C. Xiong D. L. Xiong Z. Zhang Z. L. Wang F. . (2022). Genome-wide association study for non-photochemical quenching traits in *oryza sativa* L. Agronomy-Basel 12, 3216. doi: 10.3390/agronomy12123216, PMID: 30654563

[B86] Wei J. Xu M. Zhang D. Mi H. (2010). The role of carotenoid isomerase in maintenance of photosynthetic oxygen evolution in rice plant. Acta Biochim. Biophys. Sin. (Shanghai). 42, 457–463. doi: 10.1093/abbs/gmq044, PMID: 20705584

[B87] Weng J. H. Chen C. Y. (1987). Differences between indica and japonica rice varieties in co2 exchange-rates in response to leaf nitrogen and temperature. Photosyn. Res. 14, 171–178. doi: 10.1007/BF00032321, PMID: 24430670

[B88] Wickham H. (2011). ggplot2: Wiley interdisciplinary reviews: computational statistics. 180–185. doi: 10.1002/wics.147, PMID:

[B89] Yadavalli V. R. Balakrishnan D. Surapaneni M. Addanki K. Mesapogu S. Beerelli K. . (2022). Mapping QTLs for yield and photosynthesis-related traits in three consecutive backcross populations of Oryza sativa cultivar Cottondora Sannalu (MTU1010) and *Oryza rufipogon*. Planta 256, 68–84. doi: 10.1007/s00425-022-03983-3, PMID: 36070104

[B90] Yang J. C. Zhang J. H. Wang Z. Q. Liu K. Wang P. (2006). Post-anthesis development of inferior and superior spikelets in rice in relation to abscisic acid and ethylene. J. Exp. Bot. 57, 149–160. doi: 10.1093/jxb/erj018, PMID: 16330527

[B91] Ye Z. P. Duan S. H. Chen X. M. Duan H. L. Gao C. P. Kang H. J. . (2021). Quantifying light response of photosynthesis: addressing the long-standing limitations of non-rectangular hyperbolic model. Photosynthetica 59, 185–191. doi: 10.32615/ps.2021.009

[B92] Ye Z. P. Suggett D. J. Robakowski P. Kang H. J. (2013). A mechanistic model for the photosynthesis-light response based on the photosynthetic electron transport of photosystem II in C3 and C4 species. New Phytol. 199, 110–120. doi: 10.1111/nph.12242, PMID: 23521402

[B93] Yi Y. M. Hassan M. A. Cheng X. X. Li Y. R. Liu H. Fang W. Y. . (2023). QTL mapping and analysis for drought tolerance in rice by genome-wide association study. Front. Plant Sci. 14. doi: 10.3389/fpls.2023.1223782, PMID: 37560028 PMC10408195

[B94] Yu G. Li F. Wang X. Zhang Y. Zhou K. Yang W. . (2024). Enhancing across-population genomic prediction for maize hybrids. Plants (Basel). 13, 3105. doi: 10.3390/plants13213105, PMID: 39520023 PMC11548338

[B95] Zhang R. L. Jia G. Q. Diao X. M. (2023). geneHapR: an R package for gene haplotypic statistics and visualization. BMC Bioinf. 24, 199. doi: 10.1186/s12859-023-05318-9, PMID: 37189023 PMC10186671

[B96] Zhang B. C. Liu X. L. Qian Q. A. Liu L. F. Dong G. J. Xiong G. Y. . (2011). Golgi nucleotide sugar transporter modulates cell wall biosynthesis and plant growth in rice. Proc. Natl. Acad. Sci. Utd. States America 108, 5110–5115. doi: 10.1073/pnas.1016144108, PMID: 21383162 PMC3064376

[B97] Zhou Y. Chebotarov D. Kudrna D. Llaca V. Lee S. Rajasekar S. . (2020). A platinum standard pan-genome resource that represents the population structure of Asian rice. Sci. Data 7, 113. doi: 10.1038/s41597-020-0438-2, PMID: 32265447 PMC7138821

[B98] Zhu X. G. Long S. P. Ort D. R. Merchant S. Briggs W. R. Ort D. (2010). Improving photosynthetic efficiency for greater yield. Annu. Rev. Plant Biol. 61, 235–261. doi: 10.1146/annurev-arplant-042809-112206, PMID: 20192734

[B99] Zhu X. G. Ort D. R. Parry M. A. von Caemmerer S. (2020). A wish list for synthetic biology in photosynthesis research. J. Exp. Bot. 71 (7), 2219–2225. 32060550 10.1093/jxb/eraa075 PMC7134917

