# Supplementary material for: Integrative genomic approaches to unravel genomic regions and candidate genes associated with flag leaf photosynthesis at the reproductive stage in rice
Source: Front Plant Sci. 2026 Apr 23;17:1752716. doi: 10.3389/fpls.2026.1752716 (PMC13149379; doi:10.3389/fpls.2026.1752716)
Supplement: Supplementary file 1 [file DataSheet1.docx]

Supplementary Fig 1b: Pearson correlation heatmap for net photosynthetic rate (Pn, µmol CO₂ m⁻² s⁻¹), stomatal conductance (mol H₂O m⁻² s⁻¹), transpiration rate (mol m⁻² s⁻¹), and vapour pressure deficit of air (VpdA, kPa) that measured in 181 greenhouse-grown accessions. The heatmap indicated that Pn, stomatal conductance, and transpiration rate were all positively correlated with each other, whereas VpdA exhibited negative correlations with each of these gas-exchange traits.


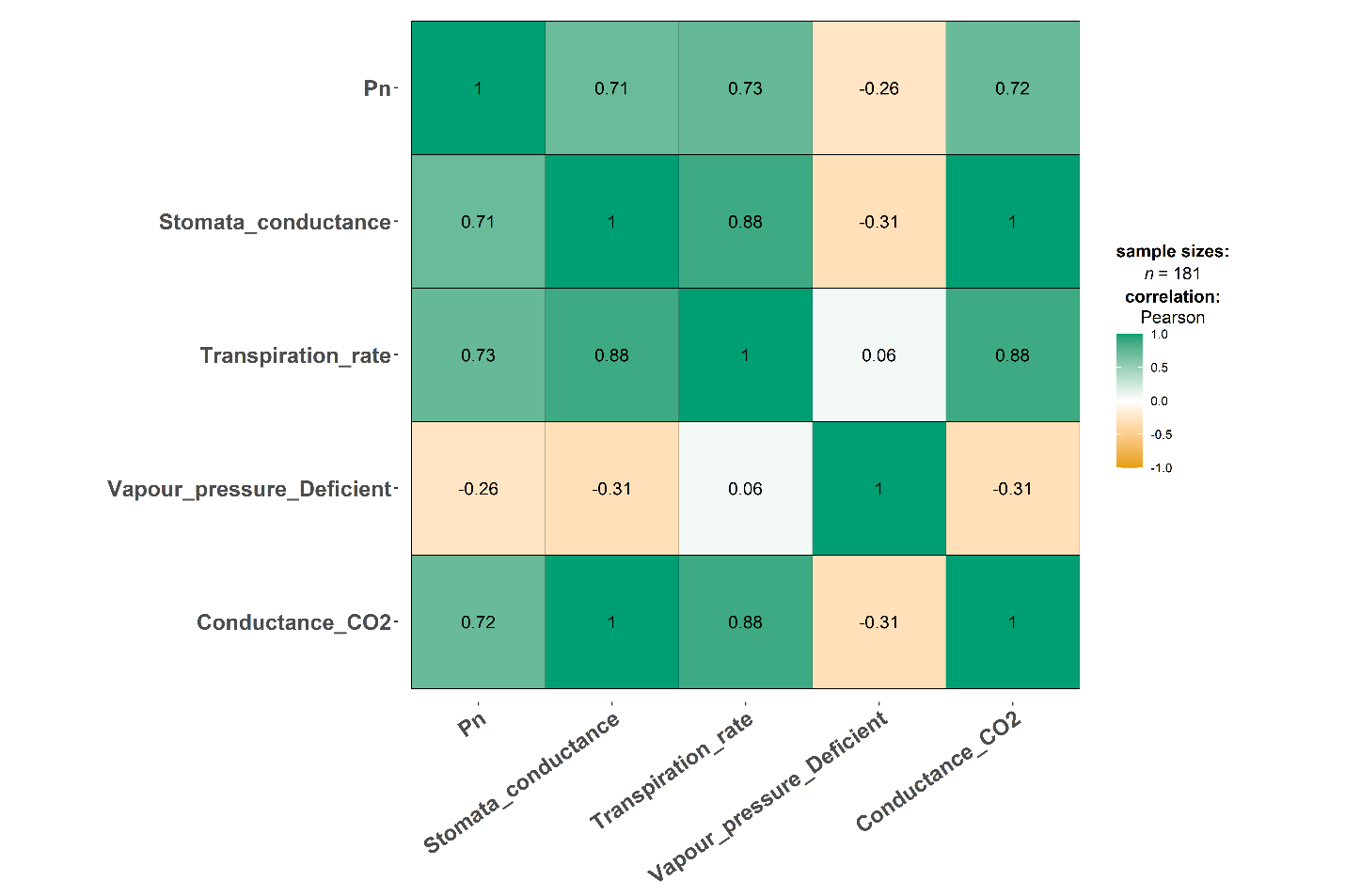


Supplementary Fig 2: Photosynthetic response of various genotypes at different level of intercellular CO_2_ (Ci) concentration (ppm). The figure showed that Pn values increased across all genotypes with increased in CO2 levels from 0 to 1400 ppm. All genotypes showed similar Pn response at low to ambient CO_2_ levels except N22, which exhibited low Pn value. A similar pattern was observed at high CO_2_ levels, where 310080 showed high Pn value followed by Zhe733, cypress and 310045. While N22 had lowest Pn value.


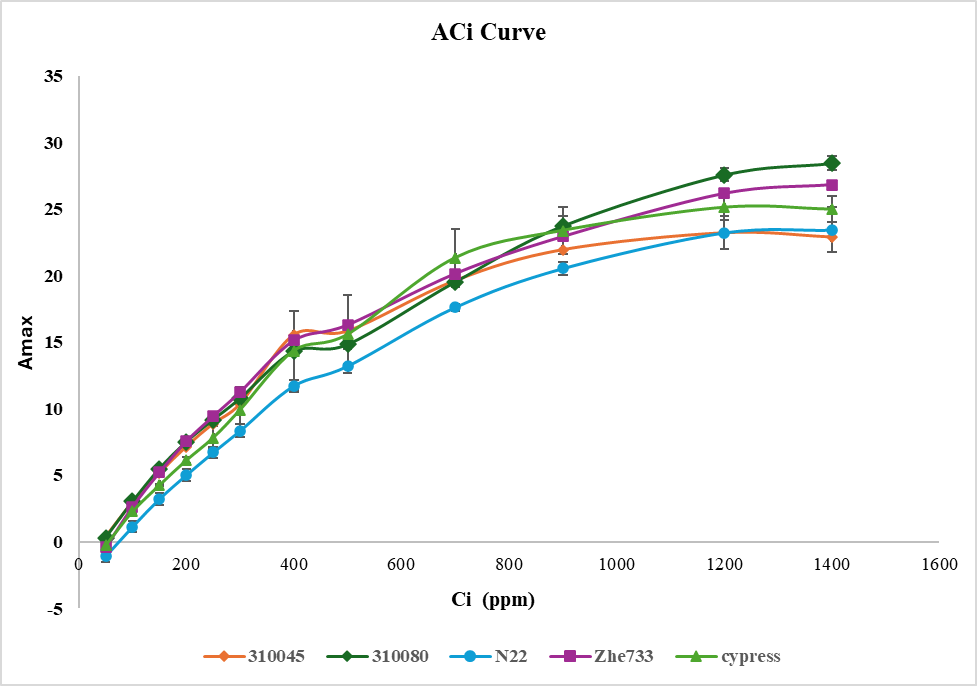

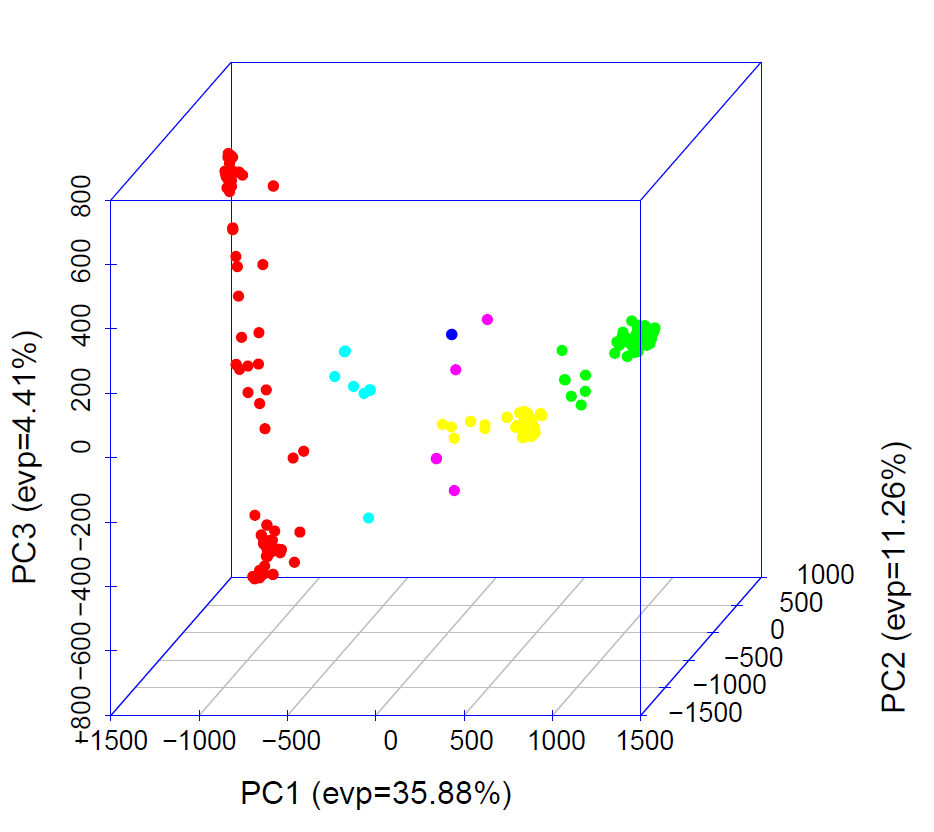


Supplementary Fig 3: Principal component analysis (PCA) of URMC diversity panel. PC1 and PC2 accounted for majority of variation in population with 35.88% and 11.26% respectively, while the PC3 contributed only 4.41 % to total variation in population diversity. PCA analysis indicated that the study population had six subpopulations.


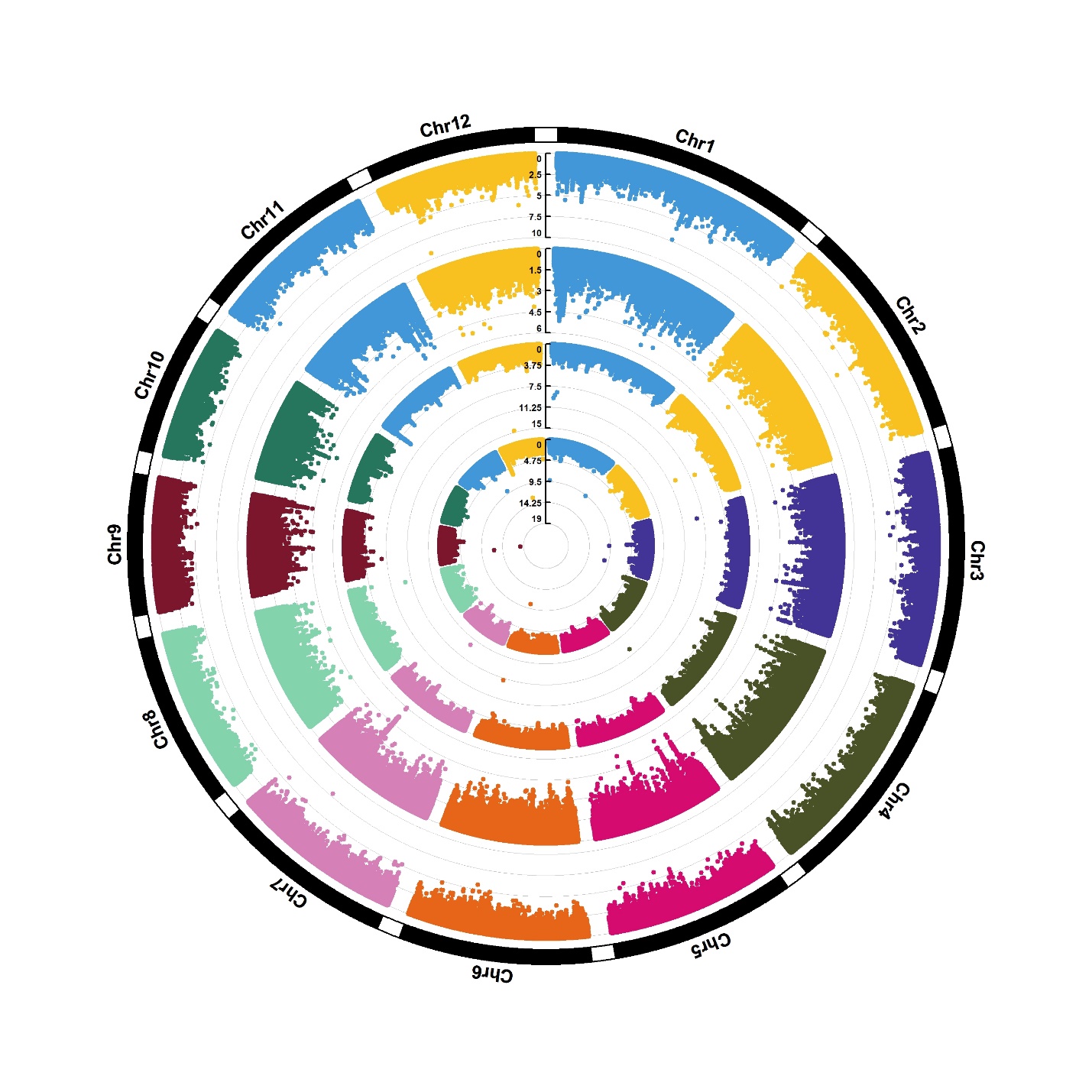


Supplementary Fig 4a: Manhattan plots for GWAS analysis based on four association models MLM, MLMM. FarmCPU and BLINK.

Supplementary Fig 4b: QQ plots showed distribution of SNPs expected values against observed values. The plot illustrated the deviation of observed SNPs’ values from expected null distribution. Significant associated SNPs across all models was deviated to right side from expected SNPs values (diagonal red spaced bar lines).


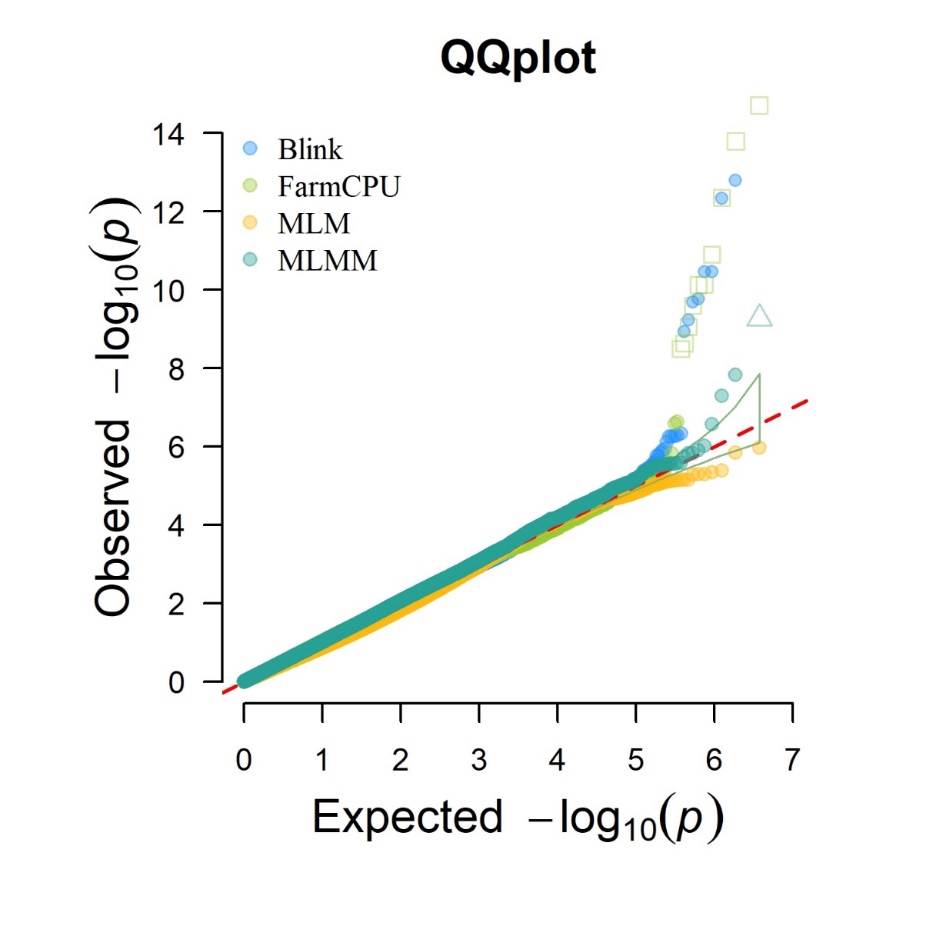

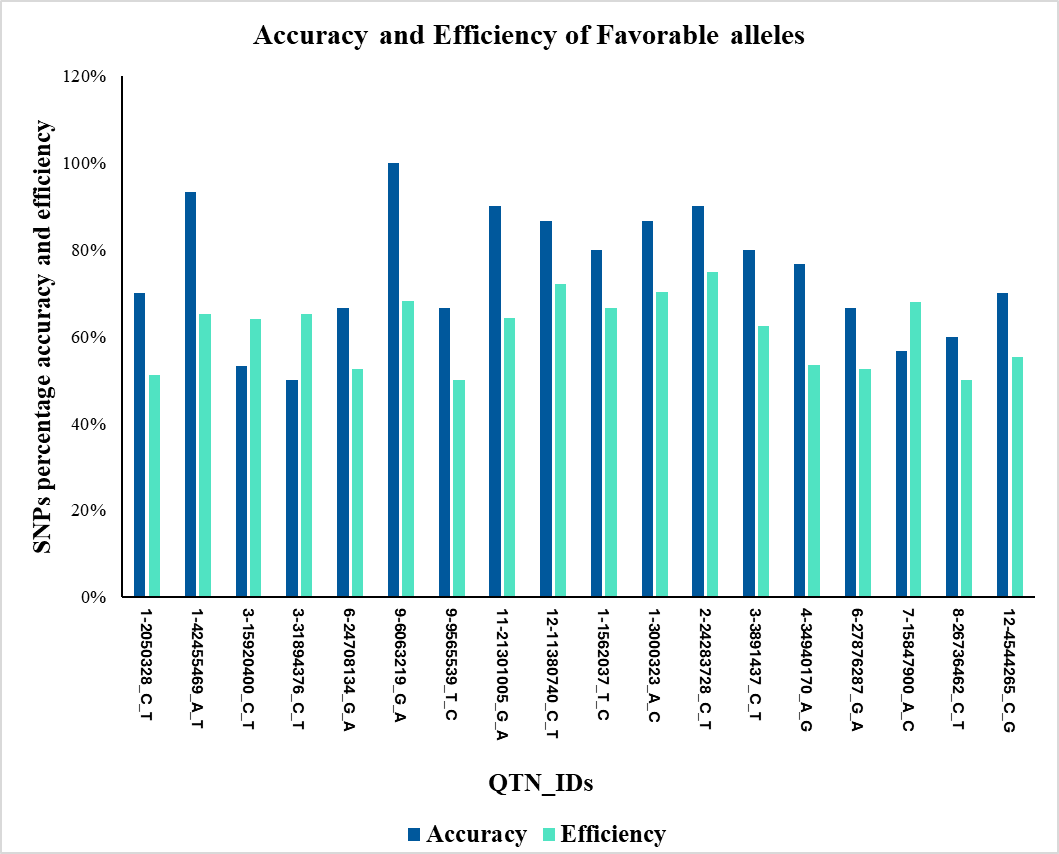


Supplementary Fig 6: GWAS derived SNP accuracy and efficiency. The bar graph illustrated that accuracy of all SNPs higher than efficiency except for SNPs on chromosome 3 and 7. The SNP “9-6063219_G_A” and “1-42455469_A_T” showed the highest accuracy, while SNPs “3-31894379” and “3-15920400” had the lowest accuracy. Similarly, SNPs “2-24283728_C_T” and “12-11380740_C_T” showed highest efficiency, whereas SNPs “8-26736462_C_T” and “9-9565539_T_C” had the lowest accuracy. Moreover, the SNPs “3-15920400_C_T”, “3-31894376_G_A”, and “7-15847900_A_C” showed higher efficiency compared to accuracy.


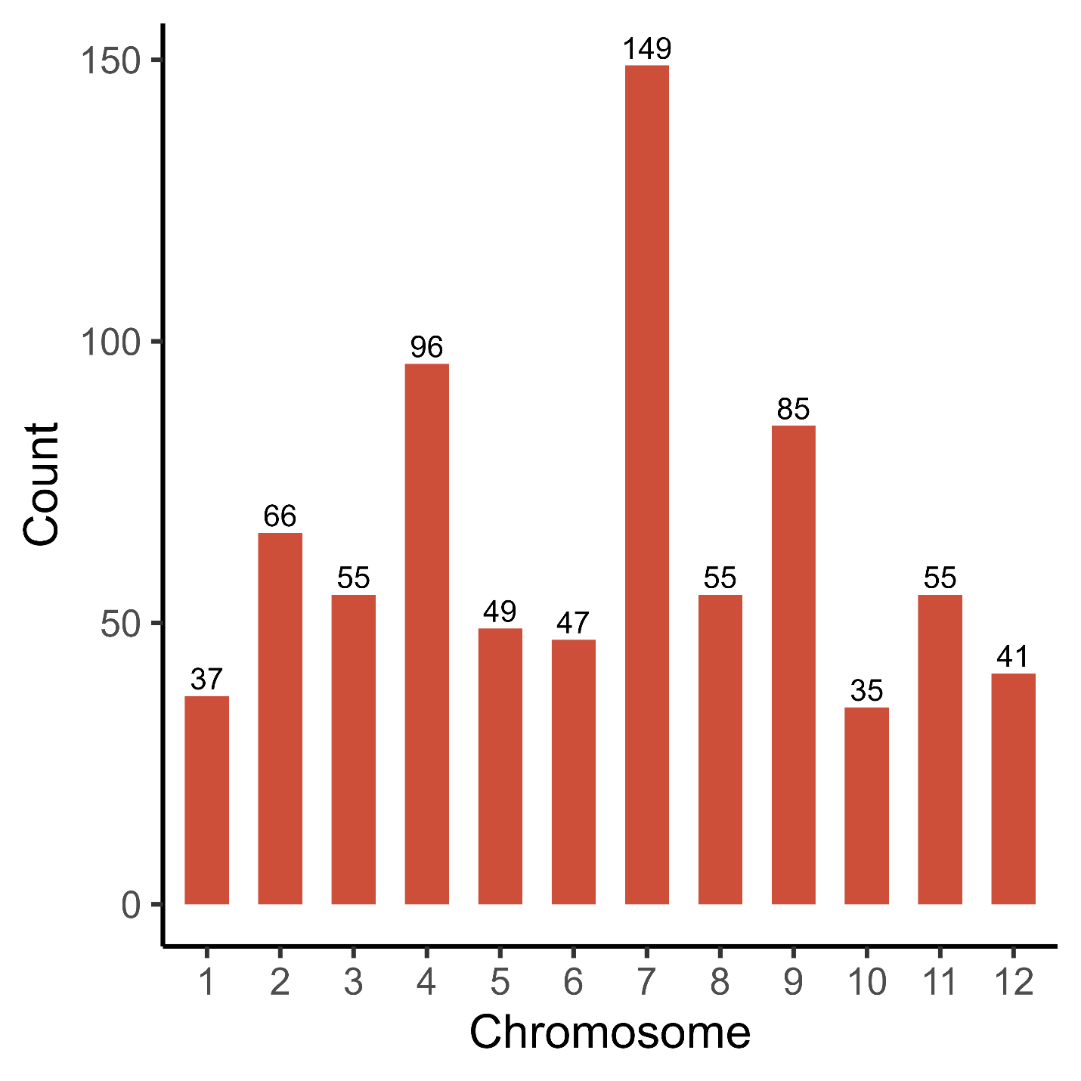


Supplementary Fig 8: SNP distribution across the genome using the GSM tools for genomic prediction using the across population. The highest number of Pn-associated SNPs were identified on chromosome 7, followed by chromosome 4 and 9, whereas the lowest number of SNPs were found on chromosome 1, followed by chromosomes 10 and 12.

**Genomic prediction models**


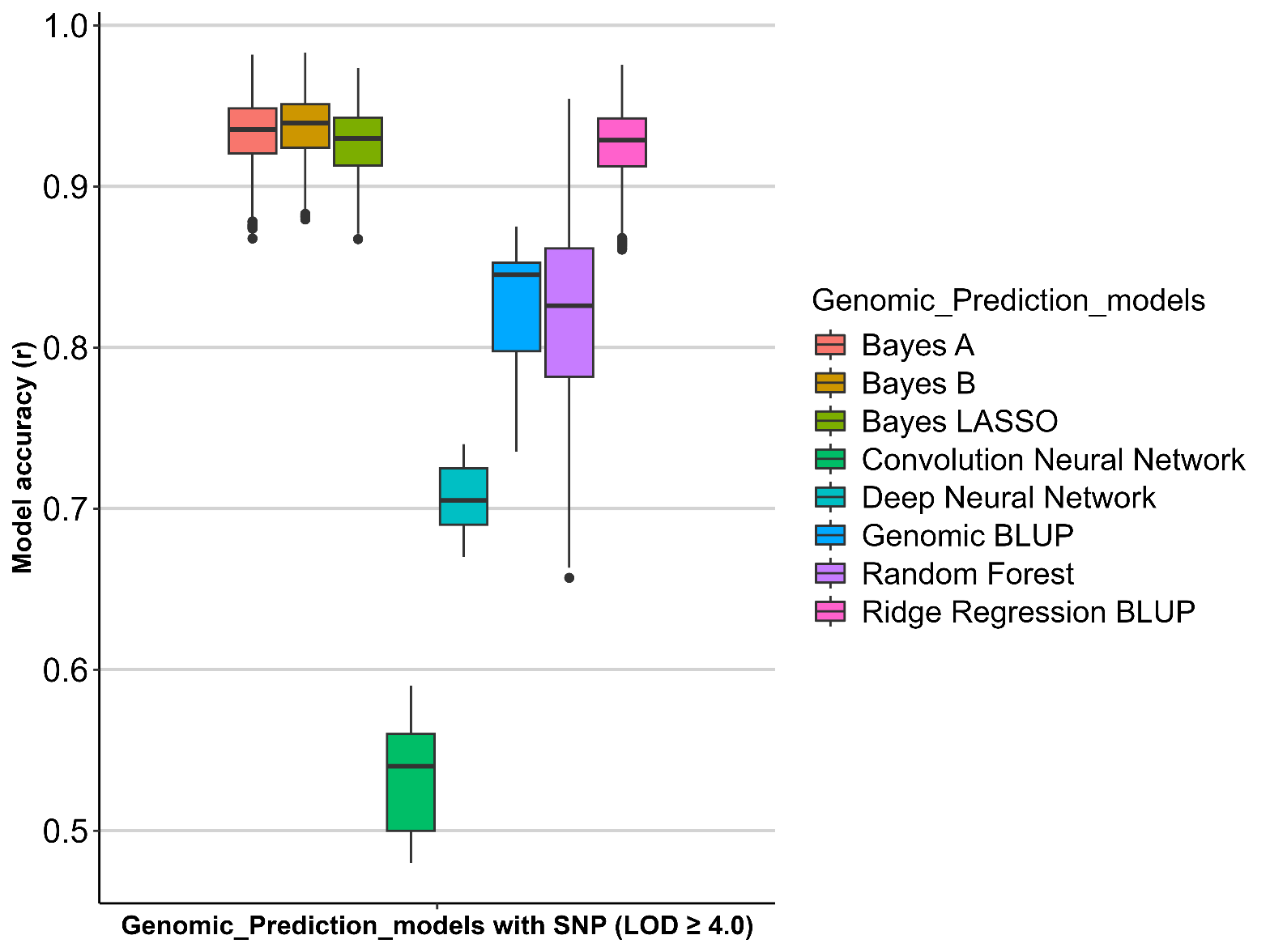


Supplementary Fig 9: Accuracy comparison among various genomic prediction models based on GWAS derived SNPs with LOD >4.0. The bayes (A, B & LASSO) models and Ridge Regression BLUP showed highest accuracy followed by Genomic BLUP and Random Forest model. The Convolution and Deep Neural Network exhibited lowest accuracy among them.
