# Supplementary material for: Integrative genomic approaches to unravel genomic regions and candidate genes associated with flag leaf photosynthesis at the reproductive stage in rice
Source: Front Plant Sci. 2026 Apr 23;17:1752716. doi: 10.3389/fpls.2026.1752716 (PMC13149379; doi:10.3389/fpls.2026.1752716)
Supplement: Supplementary file 2 [file Presentation1.pptx]

## Slide 1
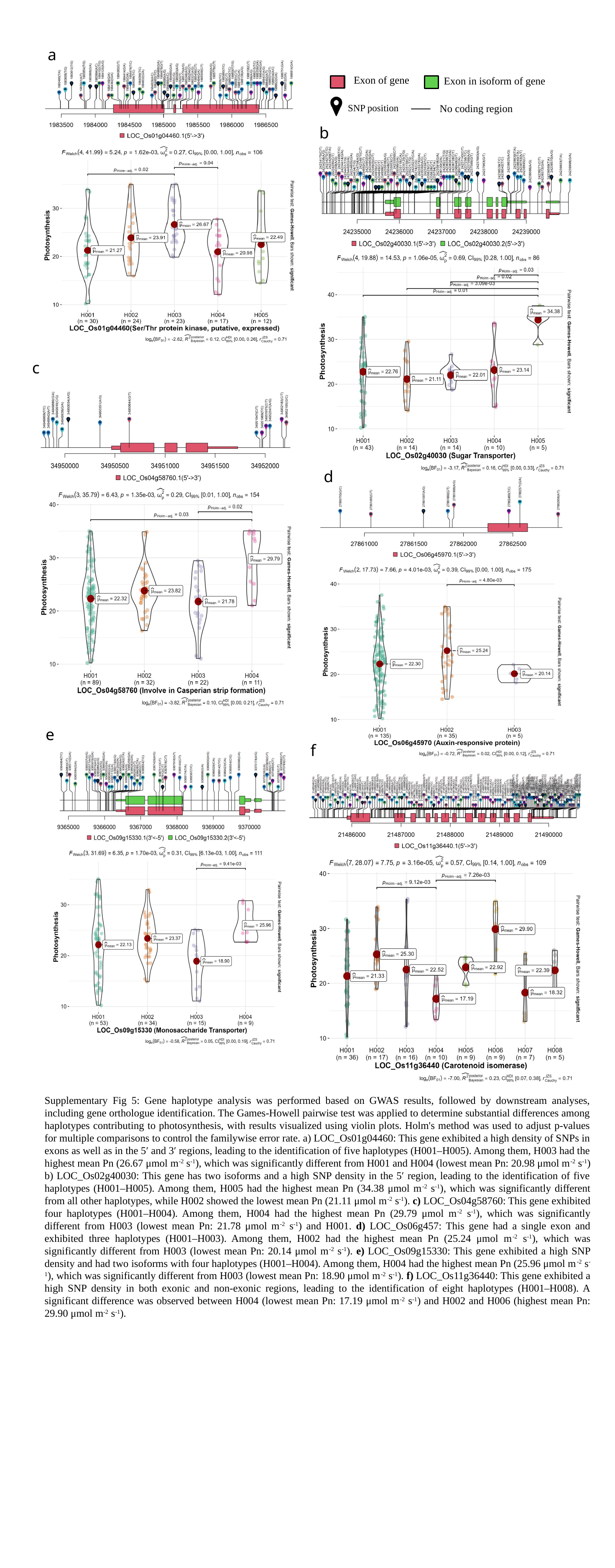

a
Exon of gene
Exon in isoform of gene
No coding region
SNP position
b
c
d
e
f
Supplementary Fig 5: Gene haplotype analysis was performed based on GWAS results, followed by downstream analyses, including gene orthologue identification. The Games-Howell pairwise test was applied to determine substantial differences among haplotypes contributing to photosynthesis, with results visualized using violin plots. Holm's method was used to adjust p-values for multiple comparisons to control the familywise error rate. a) LOC_Os01g04460: This gene exhibited a high density of SNPs in exons as well as in the 5′ and 3′ regions, leading to the identification of five haplotypes (H001–H005). Among them, H003 had the highest mean Pn (26.67 μmol m-2 s-1), which was significantly different from H001 and H004 (lowest mean Pn: 20.98 μmol m-2 s-1) b) LOC_Os02g40030: This gene has two isoforms and a high SNP density in the 5′ region, leading to the identification of five haplotypes (H001–H005). Among them, H005 had the highest mean Pn (34.38 μmol m-2 s-1), which was significantly different from all other haplotypes, while H002 showed the lowest mean Pn (21.11 μmol m-2 s-1). c) LOC_Os04g58760: This gene exhibited four haplotypes (H001–H004). Among them, H004 had the highest mean Pn (29.79 μmol m-2 s-1), which was significantly different from H003 (lowest mean Pn: 21.78 μmol m-2 s-1) and H001. d) LOC_Os06g457: This gene had a single exon and exhibited three haplotypes (H001–H003). Among them, H002 had the highest mean Pn (25.24 μmol m-2 s-1), which was significantly different from H003 (lowest mean Pn: 20.14 μmol m-2 s-1). e) LOC_Os09g15330: This gene exhibited a high SNP density and had two isoforms with four haplotypes (H001–H004). Among them, H004 had the highest mean Pn (25.96 μmol m-2 s-1), which was significantly different from H003 (lowest mean Pn: 18.90 μmol m-2 s-1). f) LOC_Os11g36440: This gene exhibited a high SNP density in both exonic and non-exonic regions, leading to the identification of eight haplotypes (H001–H008). A significant difference was observed between H004 (lowest mean Pn: 17.19 μmol m-2 s-1) and H002 and H006 (highest mean Pn: 29.90 μmol m-2 s-1).
