# Supplementary material for: Integrative genomic approaches to unravel genomic regions and candidate genes associated with flag leaf photosynthesis at the reproductive stage in rice
Source: Front Plant Sci. 2026 Apr 23;17:1752716. doi: 10.3389/fpls.2026.1752716 (PMC13149379; doi:10.3389/fpls.2026.1752716)
Supplement: Supplementary file 3 [file Presentation2.pptx]

## Slide 1
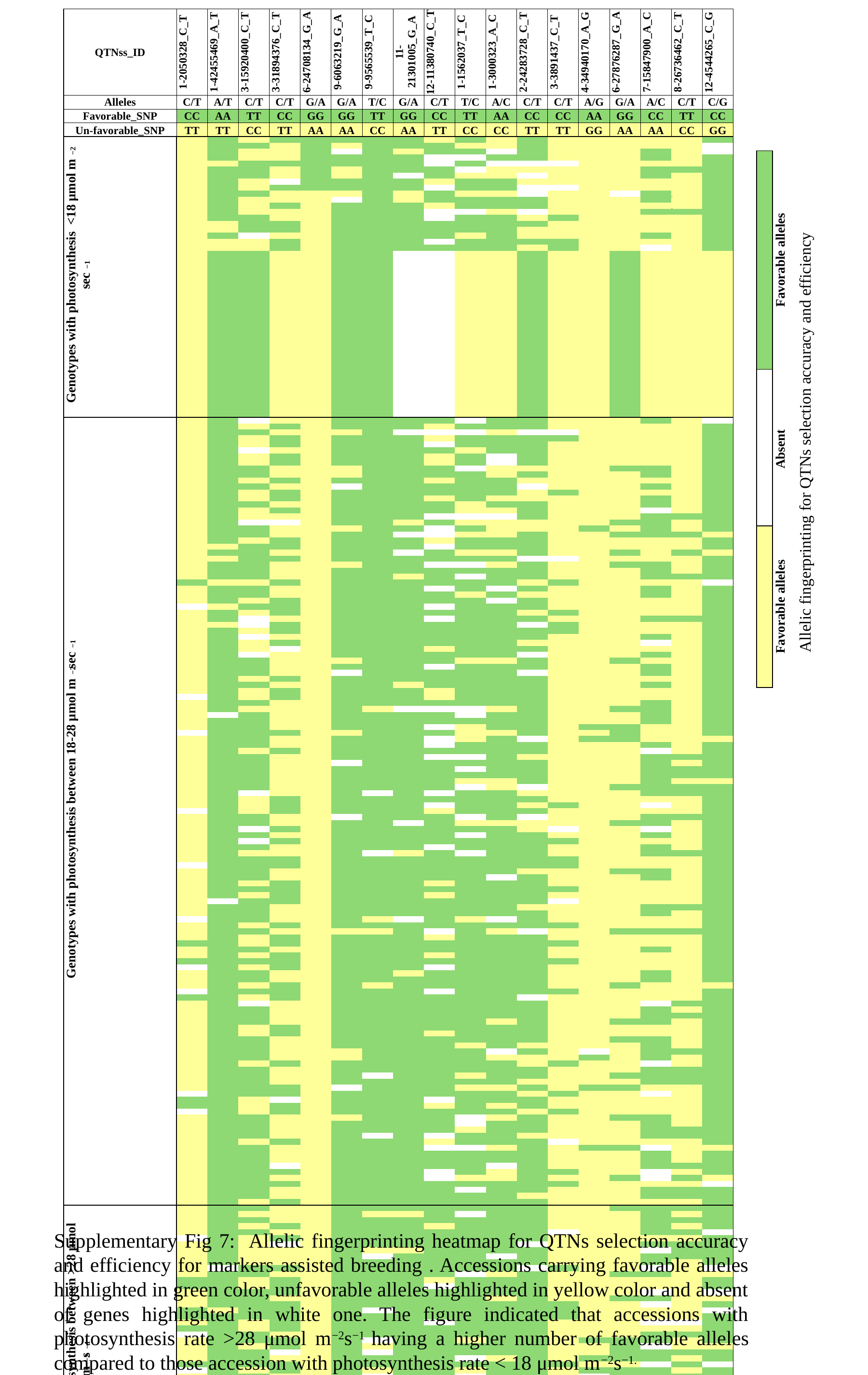

| QTNss\_ID | 1-2050328\_C\_T | 1-42455469\_A\_T | 3-15920400\_C\_T | 3-31894376\_C\_T | 6-24708134\_G\_A | 9-6063219\_G\_A | 9-9565539\_T\_C | 11-21301005\_G\_A | 12-11380740\_C\_T | 1-1562037\_T\_C | 1-3000323\_A\_C | 2-24283728\_C\_T | 3-3891437\_C\_T | 4-34940170\_A\_G | 6-27876287\_G\_A | 7-15847900\_A\_C | 8-26736462\_C\_T | 12-4544265\_C\_G |
| --- | --- | --- | --- | --- | --- | --- | --- | --- | --- | --- | --- | --- | --- | --- | --- | --- | --- | --- |
| Alleles | C/T | A/T | C/T | C/T | G/A | G/A | T/C | G/A | C/T | T/C | A/C | C/T | C/T | A/G | G/A | A/C | C/T | C/G |
| Favorable\_SNP | CC | AA | TT | CC | GG | GG | TT | GG | CC | TT | AA | CC | CC | AA | GG | CC | TT | CC |
| Un-favorable\_SNP | TT | TT | CC | TT | AA | AA | CC | AA | TT | CC | CC | TT | TT | GG | AA | AA | CC | GG |
| Genotypes with photosynthesis <18 μmol m−2 sec−1 | | | | | | | | | | | | | | | | | | |
| | | | | | | | | | | | | | | | | | | |
| | | | | | | | | | | | | | | | | | | |
| | | | | | | | | | | | | | | | | | | |
| | | | | | | | | | | | | | | | | | | |
| | | | | | | | | | | | | | | | | | | |
| | | | | | | | | | | | | | | | | | | |
| | | | | | | | | | | | | | | | | | | |
| | | | | | | | | | | | | | | | | | | |
| | | | | | | | | | | | | | | | | | | |
| | | | | | | | | | | | | | | | | | | |
| | | | | | | | | | | | | | | | | | | |
| | | | | | | | | | | | | | | | | | | |
| | | | | | | | | | | | | | | | | | | |
| | | | | | | | | | | | | | | | | | | |
| | | | | | | | | | | | | | | | | | | |
| | | | | | | | | | | | | | | | | | | |
| | | | | | | | | | | | | | | | | | | |
| | | | | | | | | | | | | | | | | | | |
| | | | | | | | | | | | | | | | | | | |
| Genotypes with photosynthesis between 18-28 μmol m−2 sec−1 | | | | | | | | | | | | | | | | | | |
| | | | | | | | | | | | | | | | | | | |
| | | | | | | | | | | | | | | | | | | |
| | | | | | | | | | | | | | | | | | | |
| | | | | | | | | | | | | | | | | | | |
| | | | | | | | | | | | | | | | | | | |
| | | | | | | | | | | | | | | | | | | |
| | | | | | | | | | | | | | | | | | | |
| | | | | | | | | | | | | | | | | | | |
| | | | | | | | | | | | | | | | | | | |
| | | | | | | | | | | | | | | | | | | |
| | | | | | | | | | | | | | | | | | | |
| | | | | | | | | | | | | | | | | | | |
| | | | | | | | | | | | | | | | | | | |
| | | | | | | | | | | | | | | | | | | |
| | | | | | | | | | | | | | | | | | | |
| | | | | | | | | | | | | | | | | | | |
| | | | | | | | | | | | | | | | | | | |
| | | | | | | | | | | | | | | | | | | |
| | | | | | | | | | | | | | | | | | | |
| | | | | | | | | | | | | | | | | | | |
| | | | | | | | | | | | | | | | | | | |
| | | | | | | | | | | | | | | | | | | |
| | | | | | | | | | | | | | | | | | | |
| | | | | | | | | | | | | | | | | | | |
| | | | | | | | | | | | | | | | | | | |
| | | | | | | | | | | | | | | | | | | |
| | | | | | | | | | | | | | | | | | | |
| | | | | | | | | | | | | | | | | | | |
| | | | | | | | | | | | | | | | | | | |
| | | | | | | | | | | | | | | | | | | |
| | | | | | | | | | | | | | | | | | | |
| | | | | | | | | | | | | | | | | | | |
| | | | | | | | | | | | | | | | | | | |
| | | | | | | | | | | | | | | | | | | |
| | | | | | | | | | | | | | | | | | | |
| | | | | | | | | | | | | | | | | | | |
| | | | | | | | | | | | | | | | | | | |
| | | | | | | | | | | | | | | | | | | |
| | | | | | | | | | | | | | | | | | | |
| | | | | | | | | | | | | | | | | | | |
| | | | | | | | | | | | | | | | | | | |
| | | | | | | | | | | | | | | | | | | |
| | | | | | | | | | | | | | | | | | | |
| | | | | | | | | | | | | | | | | | | |
| | | | | | | | | | | | | | | | | | | |
| | | | | | | | | | | | | | | | | | | |
| | | | | | | | | | | | | | | | | | | |
| | | | | | | | | | | | | | | | | | | |
| | | | | | | | | | | | | | | | | | | |
| | | | | | | | | | | | | | | | | | | |
| | | | | | | | | | | | | | | | | | | |
| | | | | | | | | | | | | | | | | | | |
| | | | | | | | | | | | | | | | | | | |
| | | | | | | | | | | | | | | | | | | |
| | | | | | | | | | | | | | | | | | | |
| | | | | | | | | | | | | | | | | | | |
| | | | | | | | | | | | | | | | | | | |
| | | | | | | | | | | | | | | | | | | |
| | | | | | | | | | | | | | | | | | | |
| | | | | | | | | | | | | | | | | | | |
| | | | | | | | | | | | | | | | | | | |
| | | | | | | | | | | | | | | | | | | |
| | | | | | | | | | | | | | | | | | | |
| | | | | | | | | | | | | | | | | | | |
| | | | | | | | | | | | | | | | | | | |
| | | | | | | | | | | | | | | | | | | |
| | | | | | | | | | | | | | | | | | | |
| | | | | | | | | | | | | | | | | | | |
| | | | | | | | | | | | | | | | | | | |
| | | | | | | | | | | | | | | | | | | |
| | | | | | | | | | | | | | | | | | | |
| | | | | | | | | | | | | | | | | | | |
| | | | | | | | | | | | | | | | | | | |
| | | | | | | | | | | | | | | | | | | |
| | | | | | | | | | | | | | | | | | | |
| | | | | | | | | | | | | | | | | | | |
| | | | | | | | | | | | | | | | | | | |
| | | | | | | | | | | | | | | | | | | |
| | | | | | | | | | | | | | | | | | | |
| | | | | | | | | | | | | | | | | | | |
| | | | | | | | | | | | | | | | | | | |
| | | | | | | | | | | | | | | | | | | |
| | | | | | | | | | | | | | | | | | | |
| | | | | | | | | | | | | | | | | | | |
| | | | | | | | | | | | | | | | | | | |
| | | | | | | | | | | | | | | | | | | |
| | | | | | | | | | | | | | | | | | | |
| | | | | | | | | | | | | | | | | | | |
| | | | | | | | | | | | | | | | | | | |
| | | | | | | | | | | | | | | | | | | |
| | | | | | | | | | | | | | | | | | | |
| | | | | | | | | | | | | | | | | | | |
| | | | | | | | | | | | | | | | | | | |
| | | | | | | | | | | | | | | | | | | |
| | | | | | | | | | | | | | | | | | | |
| | | | | | | | | | | | | | | | | | | |
| | | | | | | | | | | | | | | | | | | |
| | | | | | | | | | | | | | | | | | | |
| | | | | | | | | | | | | | | | | | | |
| | | | | | | | | | | | | | | | | | | |
| | | | | | | | | | | | | | | | | | | |
| | | | | | | | | | | | | | | | | | | |
| | | | | | | | | | | | | | | | | | | |
| | | | | | | | | | | | | | | | | | | |
| | | | | | | | | | | | | | | | | | | |
| | | | | | | | | | | | | | | | | | | |
| | | | | | | | | | | | | | | | | | | |
| | | | | | | | | | | | | | | | | | | |
| | | | | | | | | | | | | | | | | | | |
| | | | | | | | | | | | | | | | | | | |
| | | | | | | | | | | | | | | | | | | |
| | | | | | | | | | | | | | | | | | | |
| | | | | | | | | | | | | | | | | | | |
| | | | | | | | | | | | | | | | | | | |
| | | | | | | | | | | | | | | | | | | |
| | | | | | | | | | | | | | | | | | | |
| | | | | | | | | | | | | | | | | | | |
| | | | | | | | | | | | | | | | | | | |
| | | | | | | | | | | | | | | | | | | |
| | | | | | | | | | | | | | | | | | | |
| | | | | | | | | | | | | | | | | | | |
| | | | | | | | | | | | | | | | | | | |
| | | | | | | | | | | | | | | | | | | |
| | | | | | | | | | | | | | | | | | | |
| | | | | | | | | | | | | | | | | | | |
| | | | | | | | | | | | | | | | | | | |
| | | | | | | | | | | | | | | | | | | |
| | | | | | | | | | | | | | | | | | | |
| | | | | | | | | | | | | | | | | | | |
| | | | | | | | | | | | | | | | | | | |
| Genotypes with photosynthesis between >28 μmol m−2s−1 | | | | | | | | | | | | | | | | | | |
| | | | | | | | | | | | | | | | | | | |
| | | | | | | | | | | | | | | | | | | |
| | | | | | | | | | | | | | | | | | | |
| | | | | | | | | | | | | | | | | | | |
| | | | | | | | | | | | | | | | | | | |
| | | | | | | | | | | | | | | | | | | |
| | | | | | | | | | | | | | | | | | | |
| | | | | | | | | | | | | | | | | | | |
| | | | | | | | | | | | | | | | | | | |
| | | | | | | | | | | | | | | | | | | |
| | | | | | | | | | | | | | | | | | | |
| | | | | | | | | | | | | | | | | | | |
| | | | | | | | | | | | | | | | | | | |
| | | | | | | | | | | | | | | | | | | |
| | | | | | | | | | | | | | | | | | | |
| | | | | | | | | | | | | | | | | | | |
| | | | | | | | | | | | | | | | | | | |
| | | | | | | | | | | | | | | | | | | |
| | | | | | | | | | | | | | | | | | | |
| | | | | | | | | | | | | | | | | | | |
| | | | | | | | | | | | | | | | | | | |
| | | | | | | | | | | | | | | | | | | |
| | | | | | | | | | | | | | | | | | | |
| | | | | | | | | | | | | | | | | | | |
| | | | | | | | | | | | | | | | | | | |
| | | | | | | | | | | | | | | | | | | |
| | | | | | | | | | | | | | | | | | | |
| | | | | | | | | | | | | | | | | | | |
| | | | | | | | | | | | | | | | | | | |
| | Favorable alleles |
| --- | --- |
| | Absent |
| | Favorable alleles |
Allelic fingerprinting for QTNs selection accuracy and efficiency
Supplementary Fig 7: Allelic fingerprinting heatmap for QTNs selection accuracy and efficiency for markers assisted breeding . Accessions carrying favorable alleles highlighted in green color, unfavorable alleles highlighted in yellow color and absent of genes highlighted in white one. The figure indicated that accessions with photosynthesis rate >28 μmol m−2s−1 having a higher number of favorable alleles compared to those accession with photosynthesis rate < 18 μmol m−2s−1.
