# Supplementary material for: Integrative genomic approaches to unravel genomic regions and candidate genes associated with flag leaf photosynthesis at the reproductive stage in rice
Source: Front Plant Sci. 2026 Apr 23;17:1752716. doi: 10.3389/fpls.2026.1752716 (PMC13149379; doi:10.3389/fpls.2026.1752716)
Supplement: Supplementary file 4 [file Presentation3.pptx]

## Slide 1
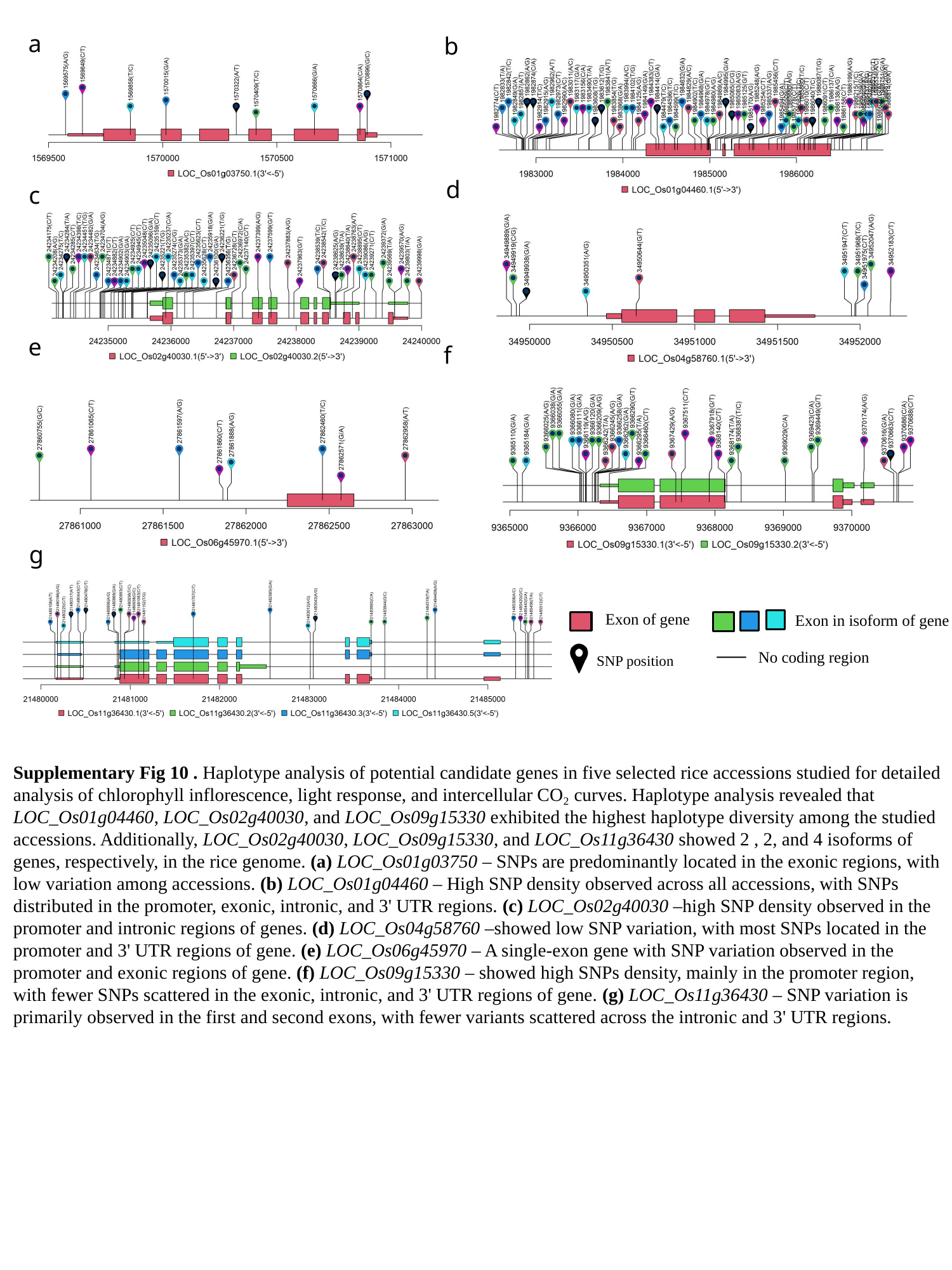

a
b
d
c
e
f
g
Exon of gene
Exon in isoform of gene
No coding region
SNP position
Supplementary Fig 10 . Haplotype analysis of potential candidate genes in five selected rice accessions studied for detailed analysis of chlorophyll inflorescence, light response, and intercellular CO₂ curves. Haplotype analysis revealed that LOC_Os01g04460, LOC_Os02g40030, and LOC_Os09g15330 exhibited the highest haplotype diversity among the studied accessions. Additionally, LOC_Os02g40030, LOC_Os09g15330, and LOC_Os11g36430 showed 2 , 2, and 4 isoforms of genes, respectively, in the rice genome. (a) LOC_Os01g03750 – SNPs are predominantly located in the exonic regions, with low variation among accessions. (b) LOC_Os01g04460 – High SNP density observed across all accessions, with SNPs distributed in the promoter, exonic, intronic, and 3' UTR regions. (c) LOC_Os02g40030 –high SNP density observed in the promoter and intronic regions of genes. (d) LOC_Os04g58760 –showed low SNP variation, with most SNPs located in the promoter and 3' UTR regions of gene. (e) LOC_Os06g45970 – A single-exon gene with SNP variation observed in the promoter and exonic regions of gene. (f) LOC_Os09g15330 – showed high SNPs density, mainly in the promoter region, with fewer SNPs scattered in the exonic, intronic, and 3' UTR regions of gene. (g) LOC_Os11g36430 – SNP variation is primarily observed in the first and second exons, with fewer variants scattered across the intronic and 3' UTR regions.
