# Supplementary material for: Integrative genomic approaches to unravel genomic regions and candidate genes associated with flag leaf photosynthesis at the reproductive stage in rice
Source: Front Plant Sci. 2026 Apr 23;17:1752716. doi: 10.3389/fpls.2026.1752716 (PMC13149379; doi:10.3389/fpls.2026.1752716)

## Slide 1
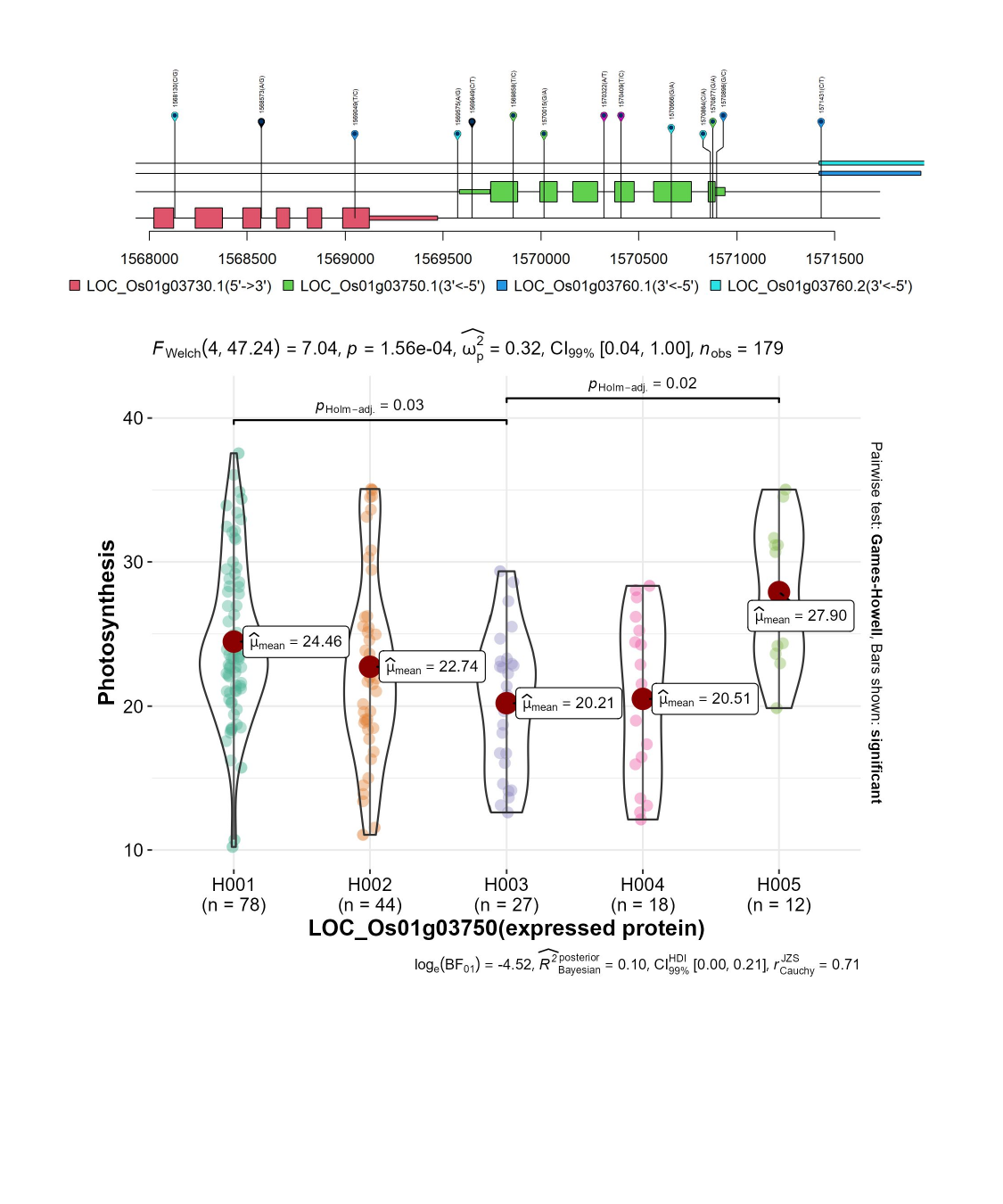

## Slide 2
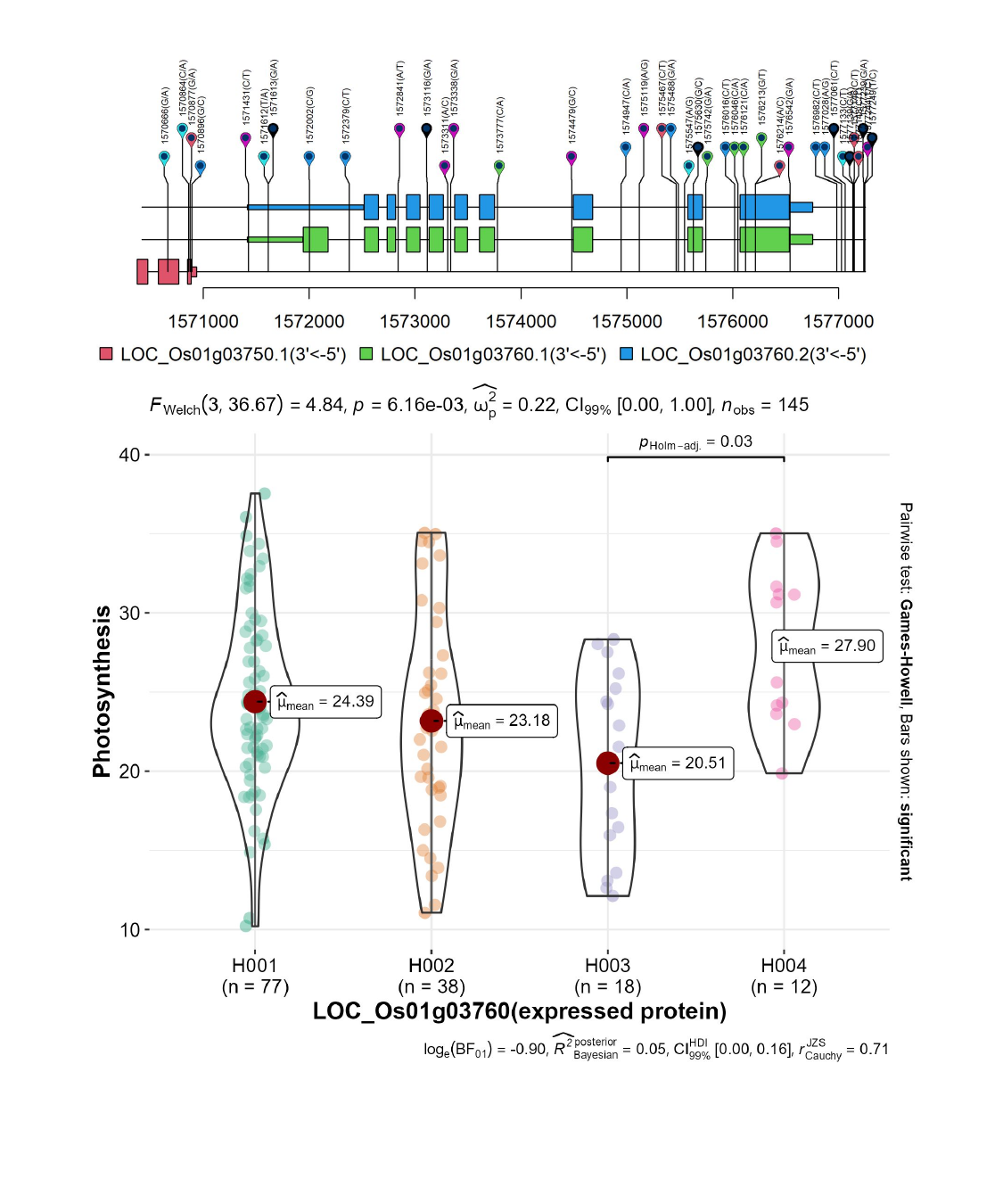

## Slide 3
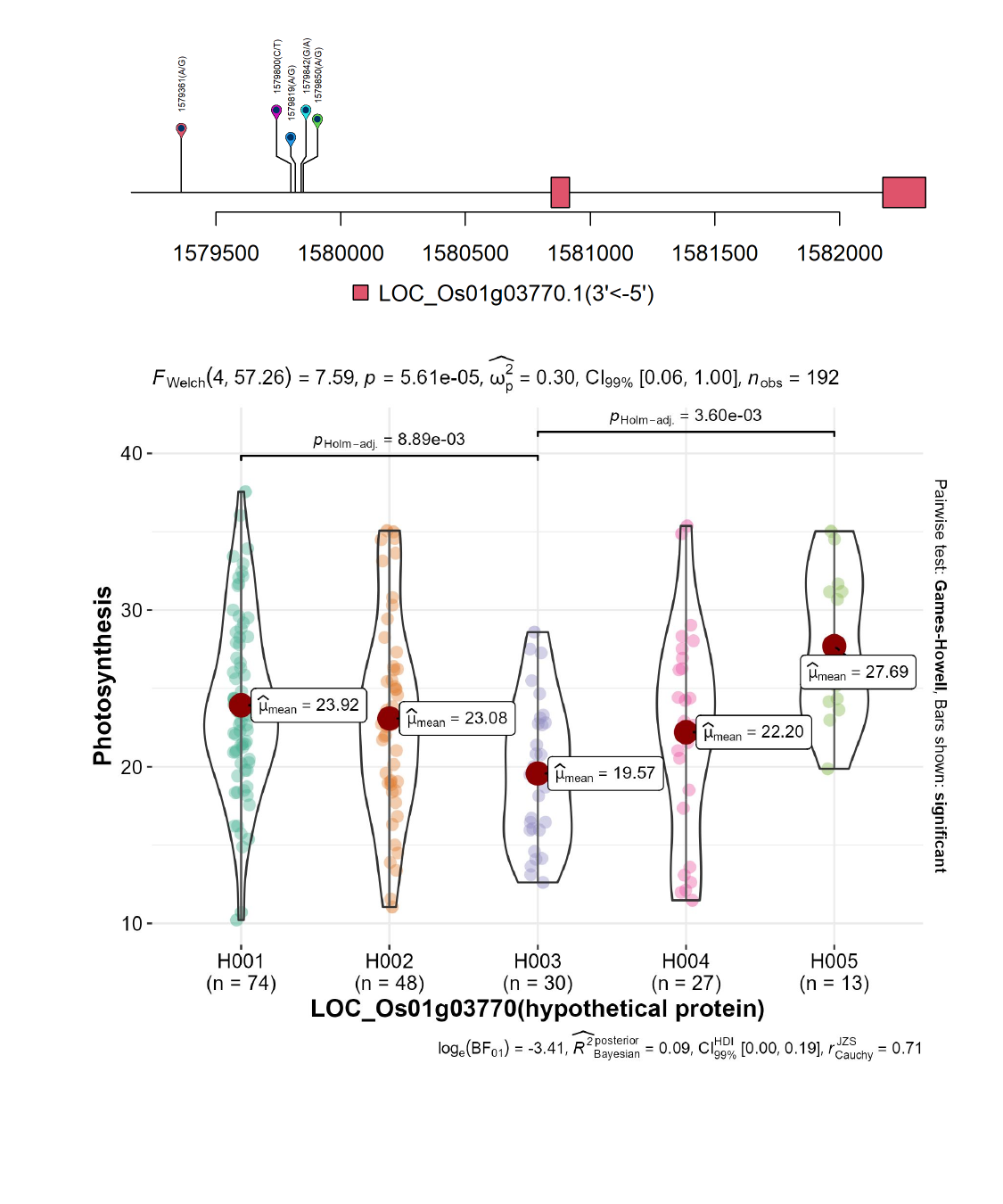

## Slide 4
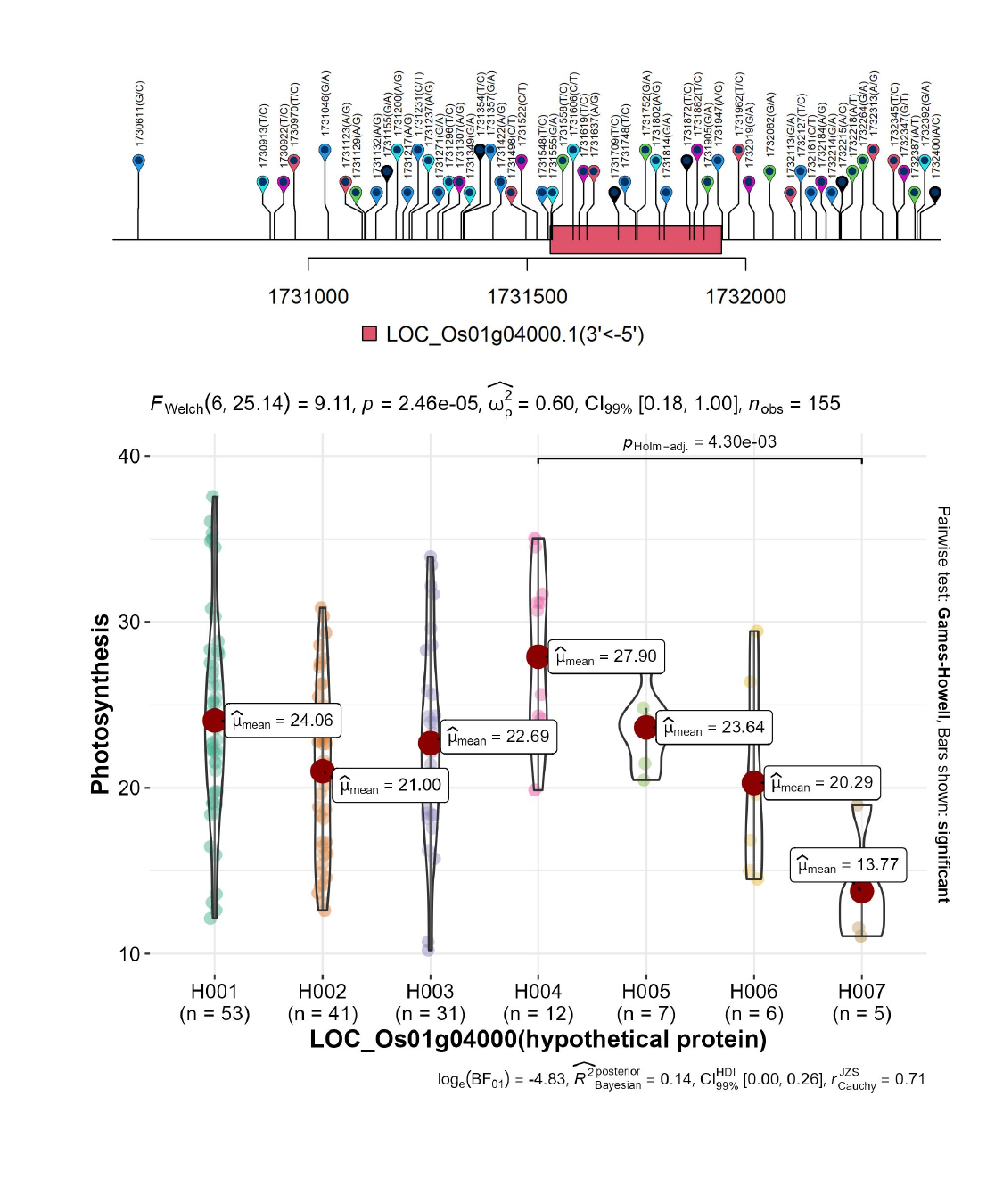

## Slide 5
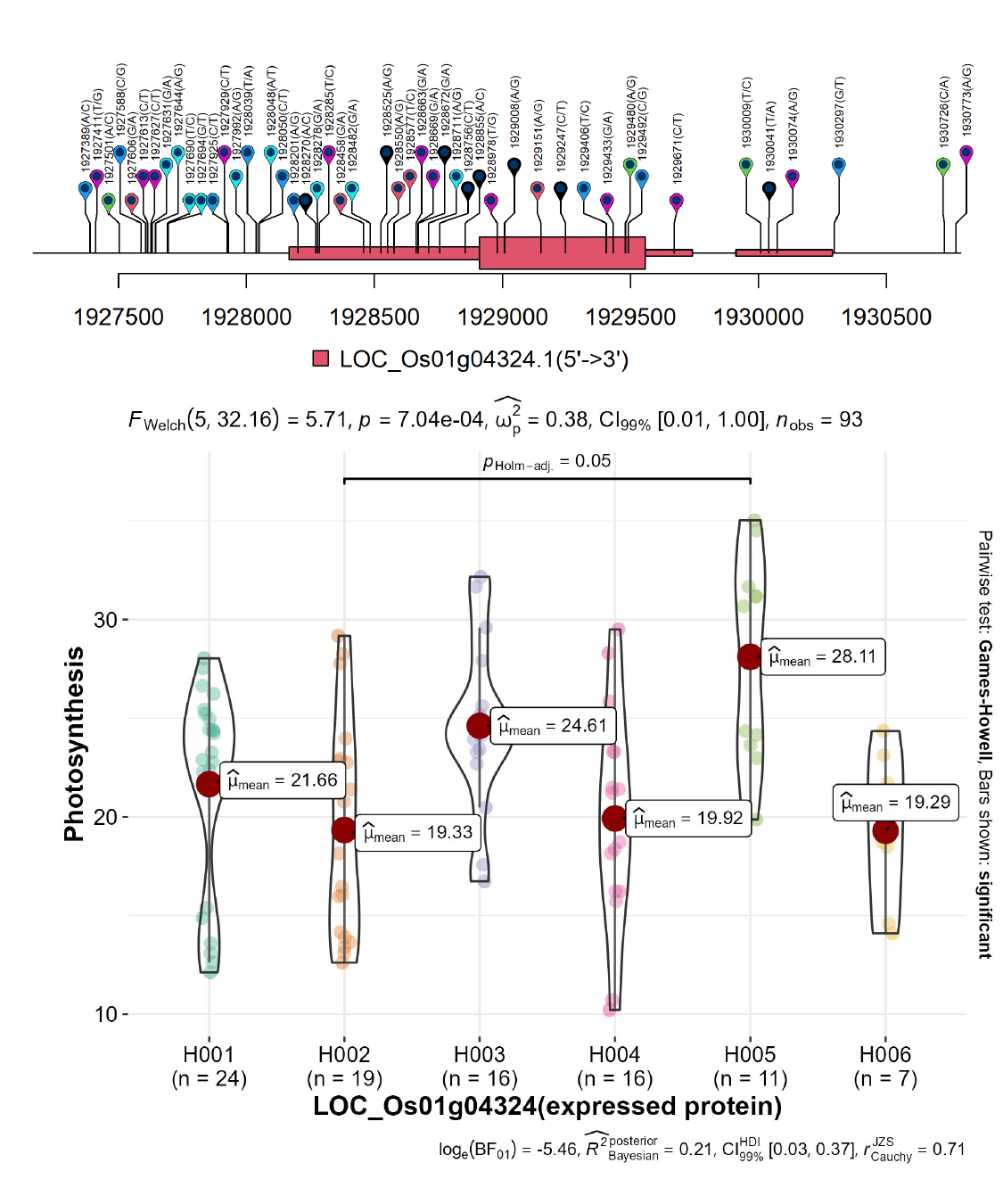

## Slide 6
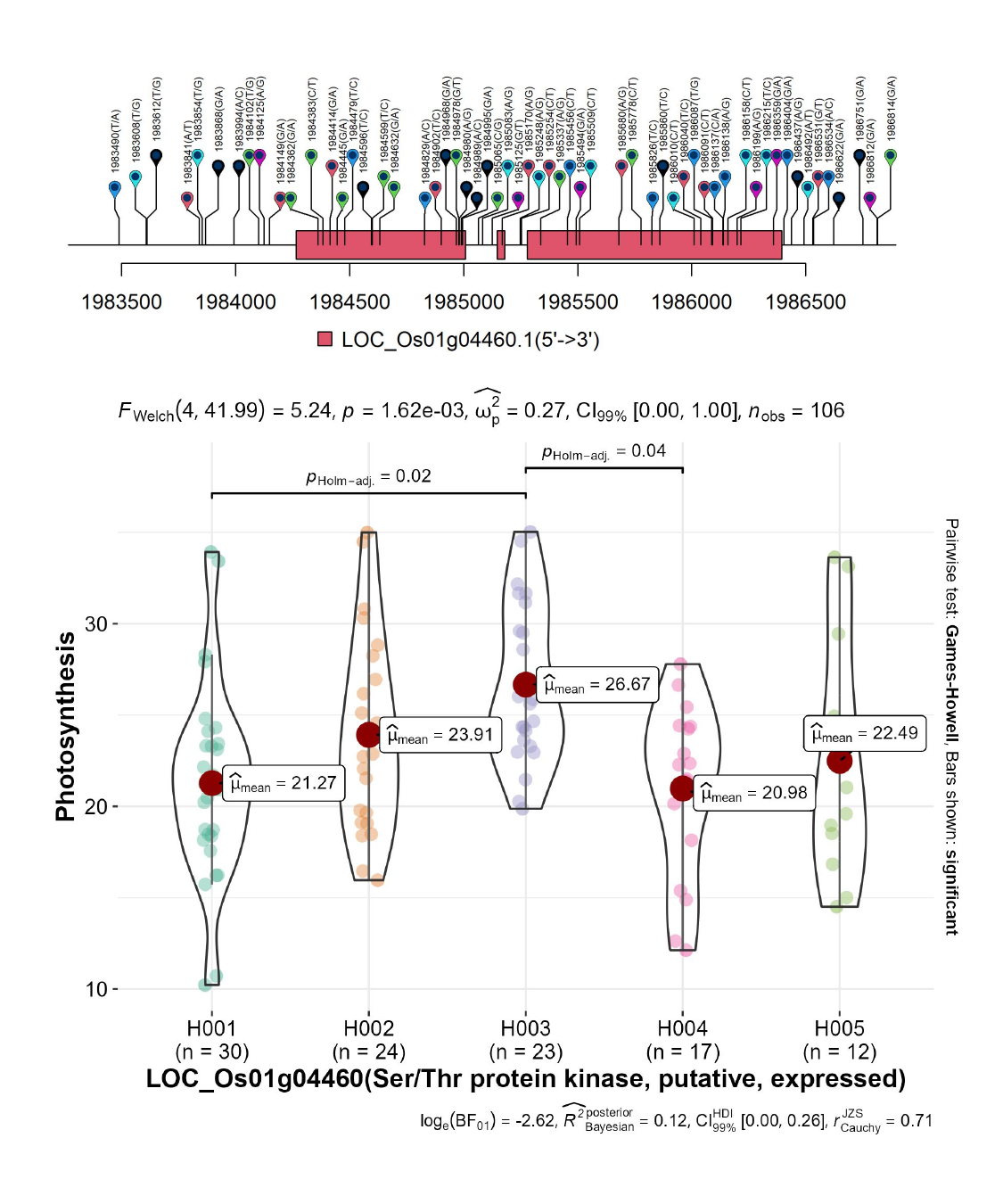

## Slide 7
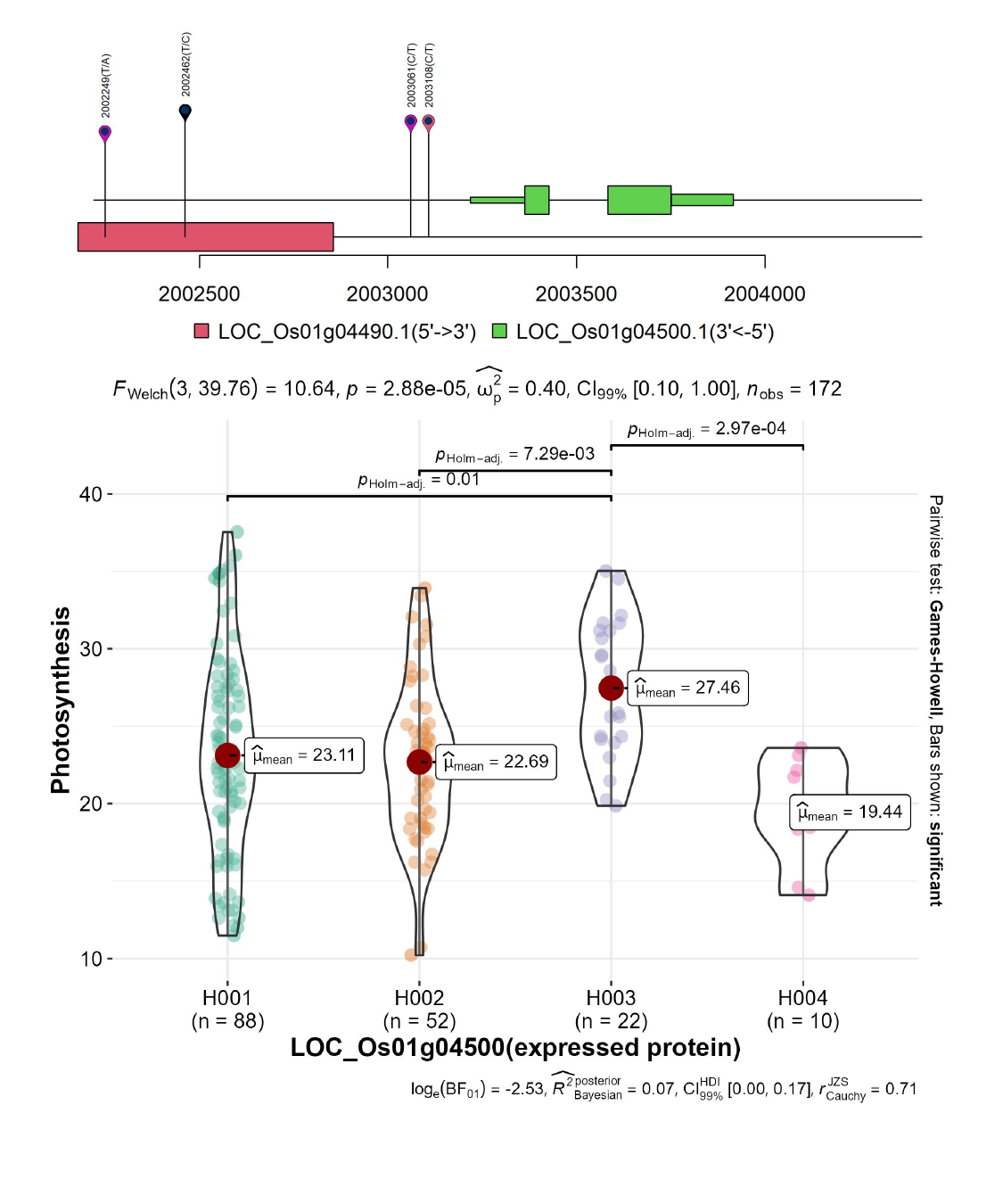

## Slide 8
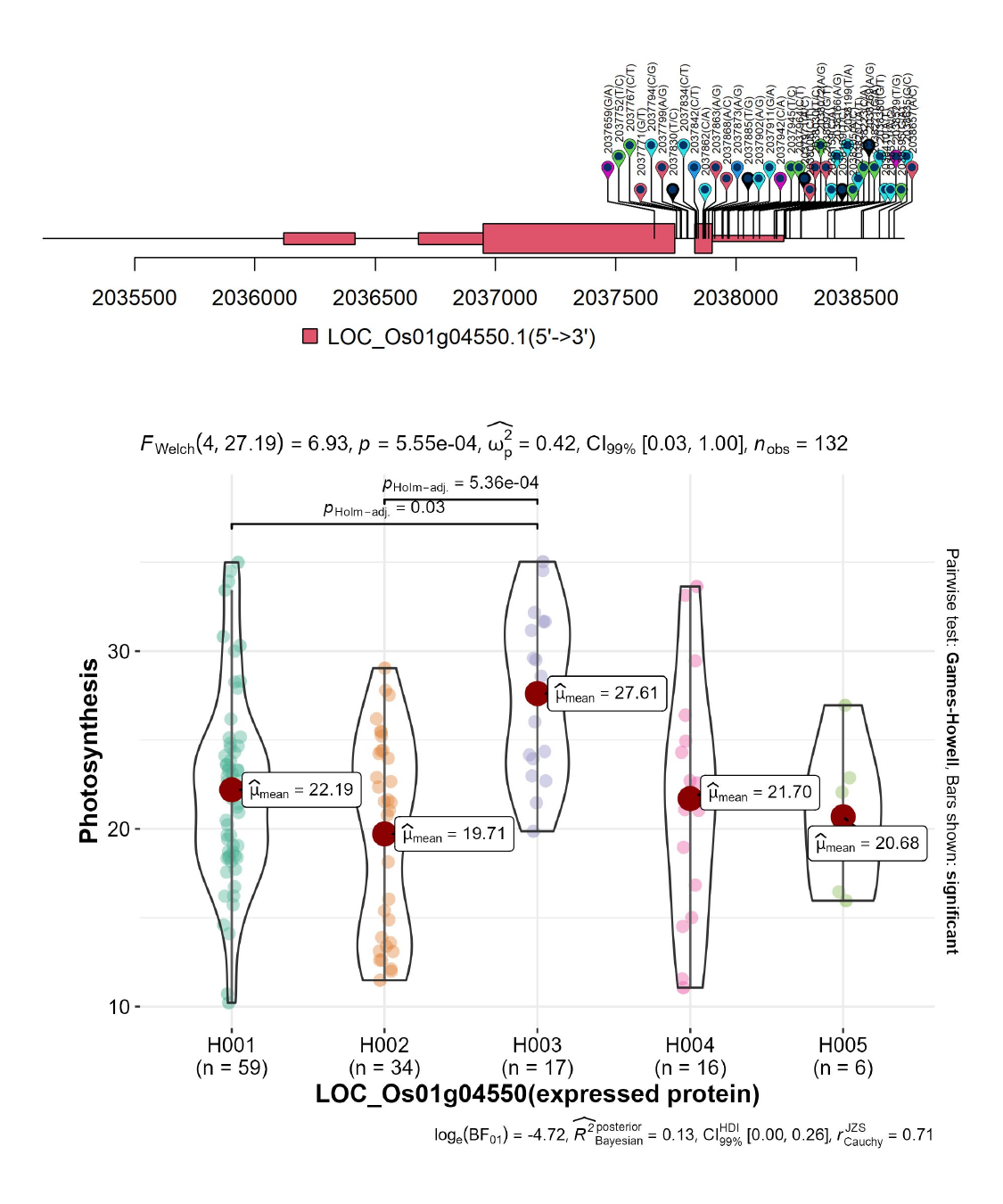

## Slide 9
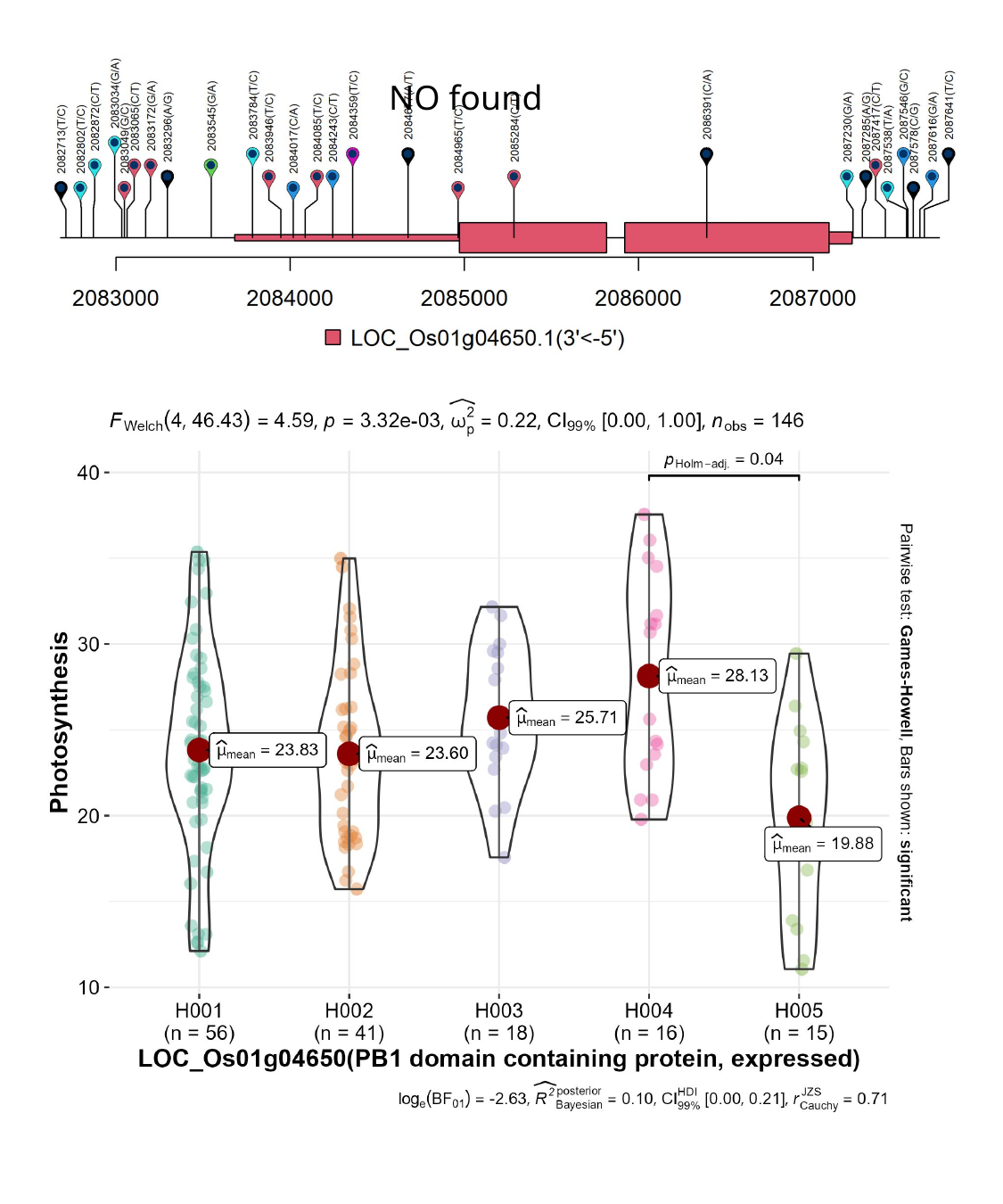

NO found

## Slide 10
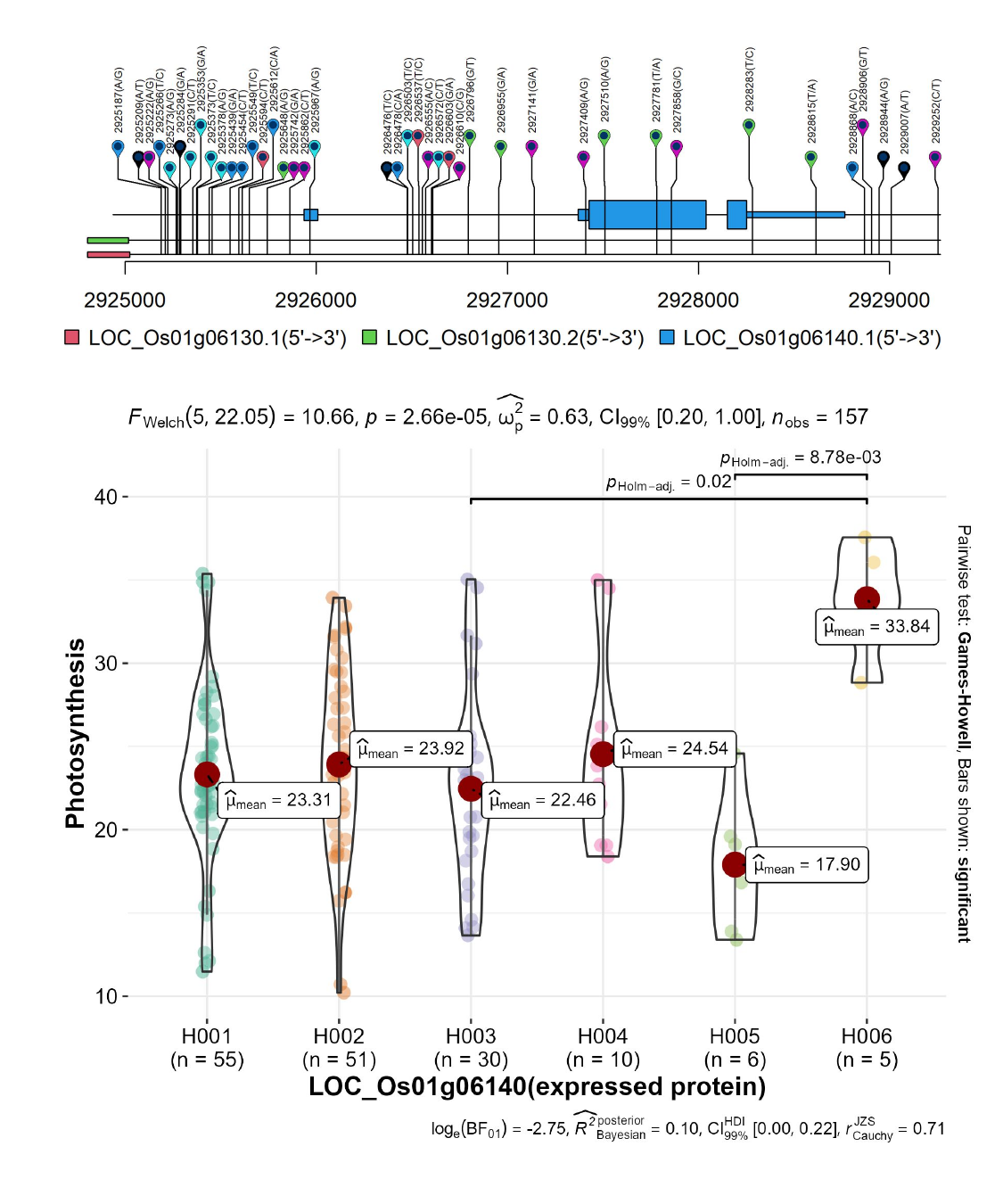

## Slide 11
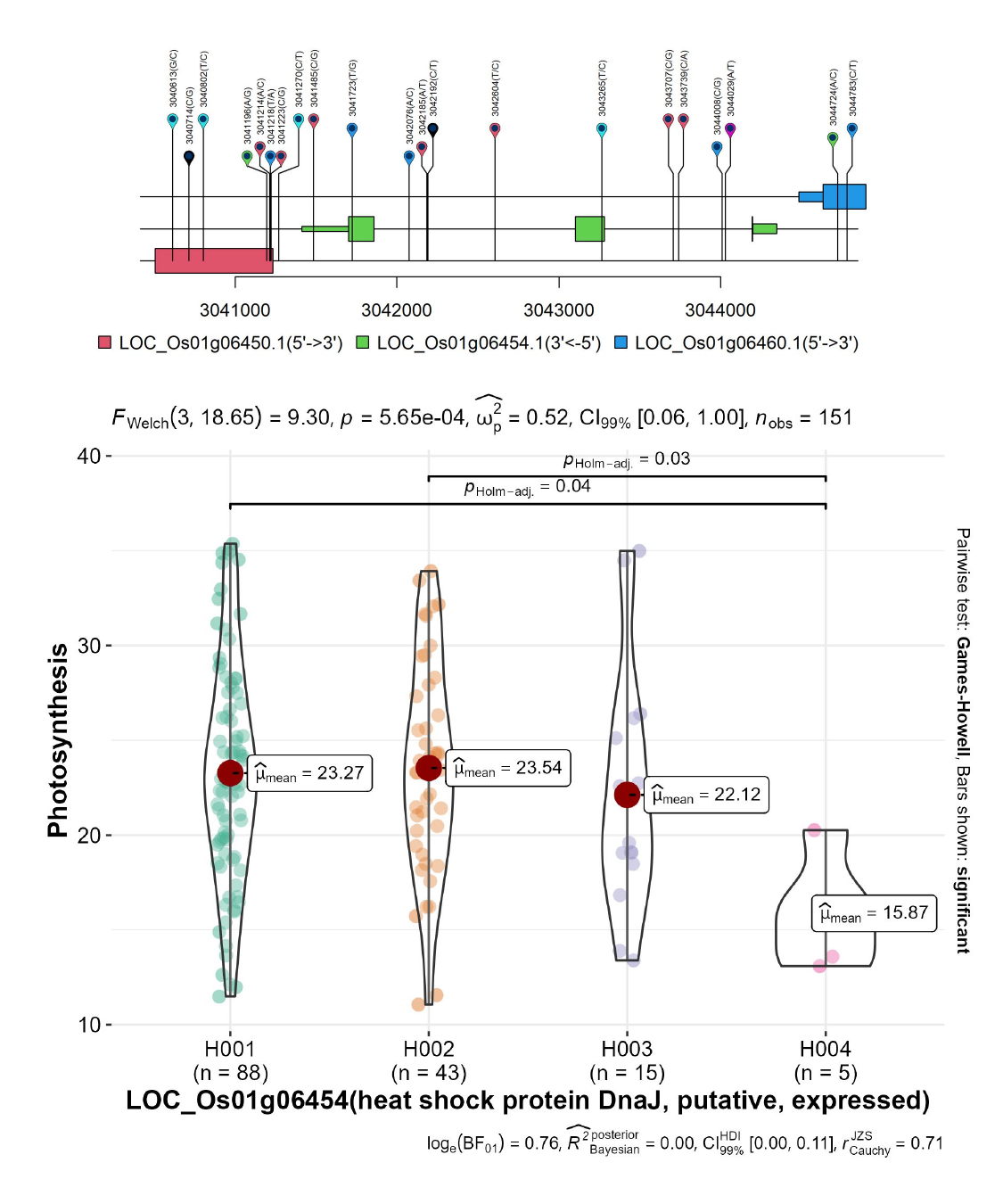

## Slide 12
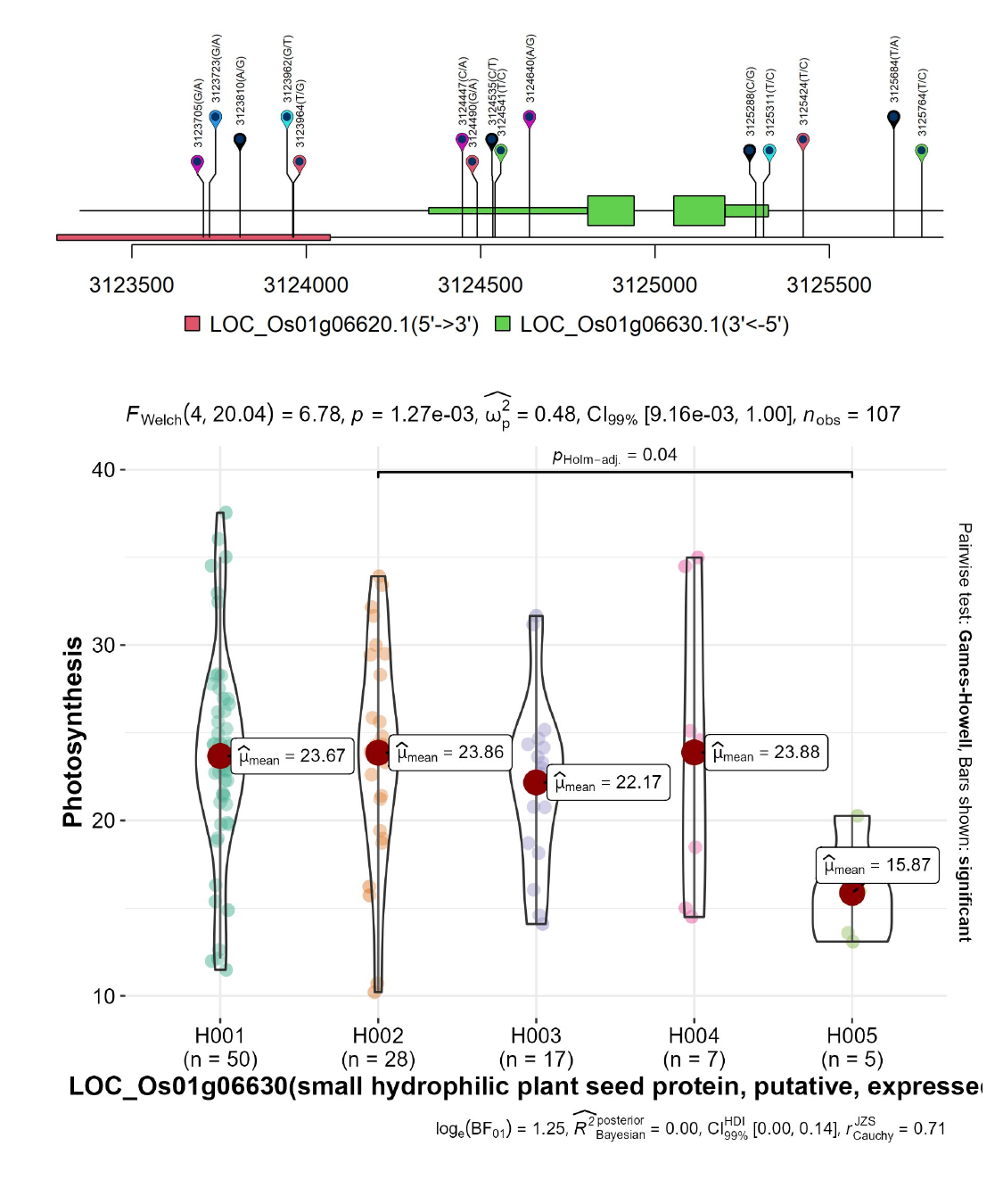

## Slide 13
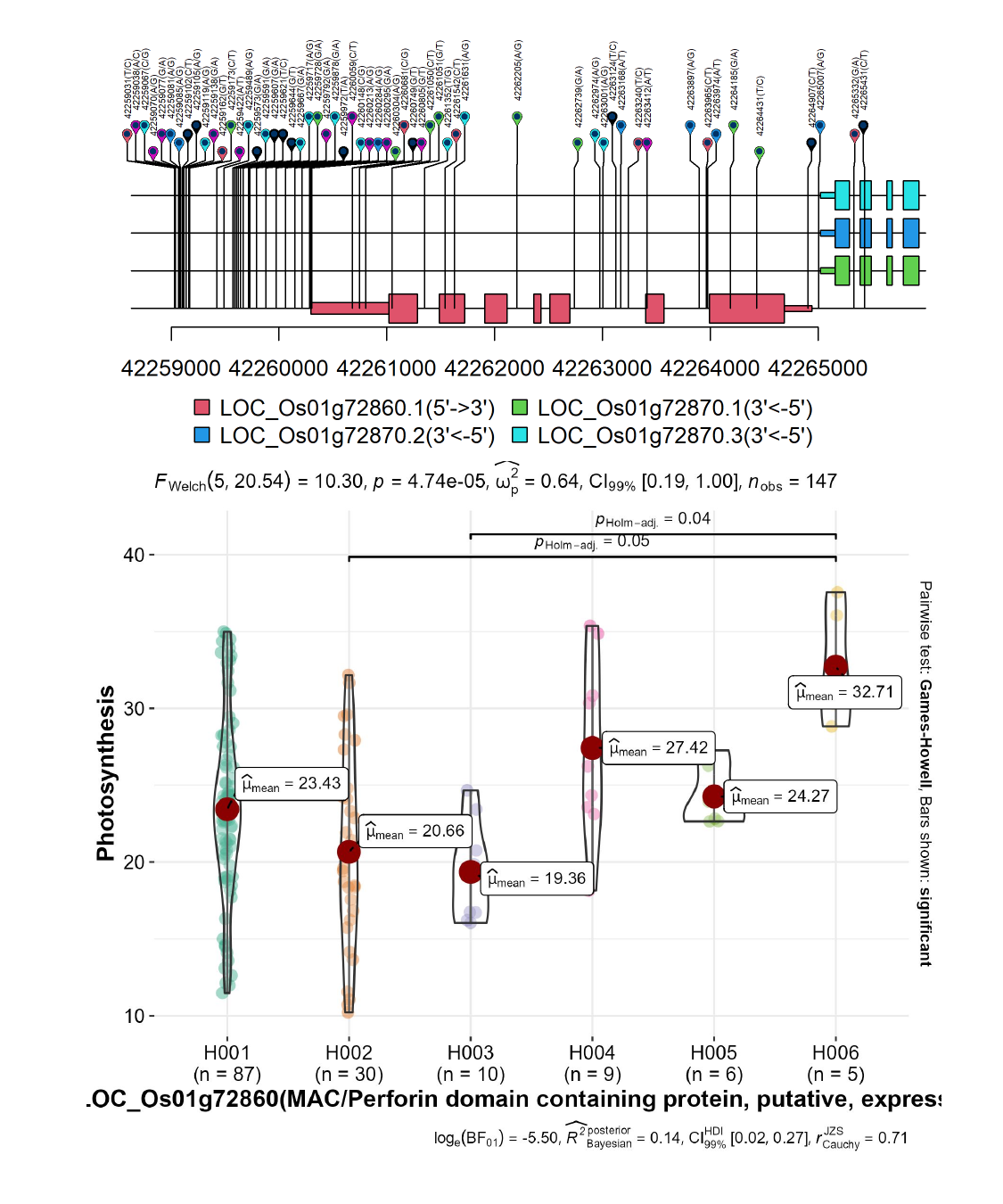

## Slide 14
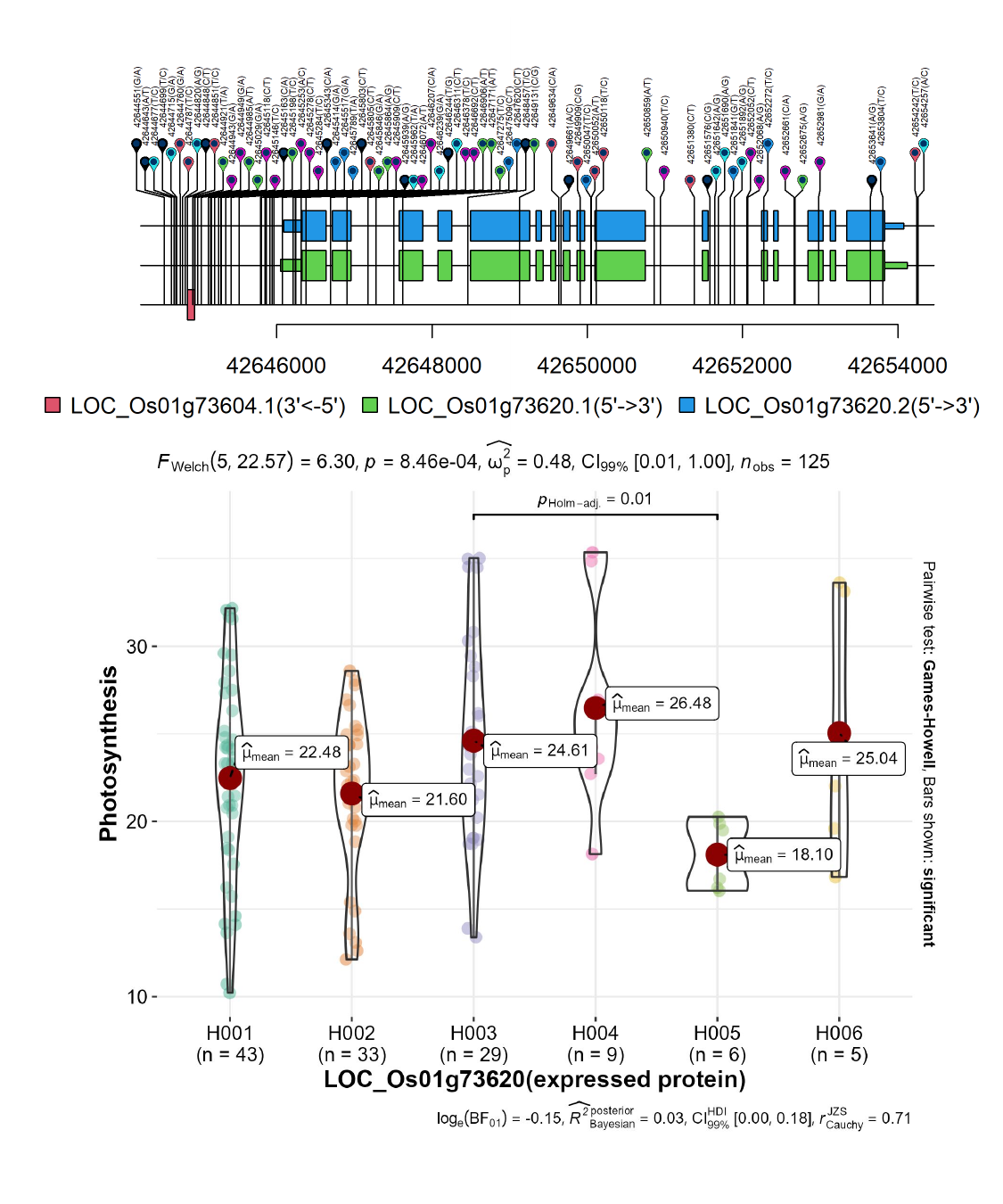

## Slide 15
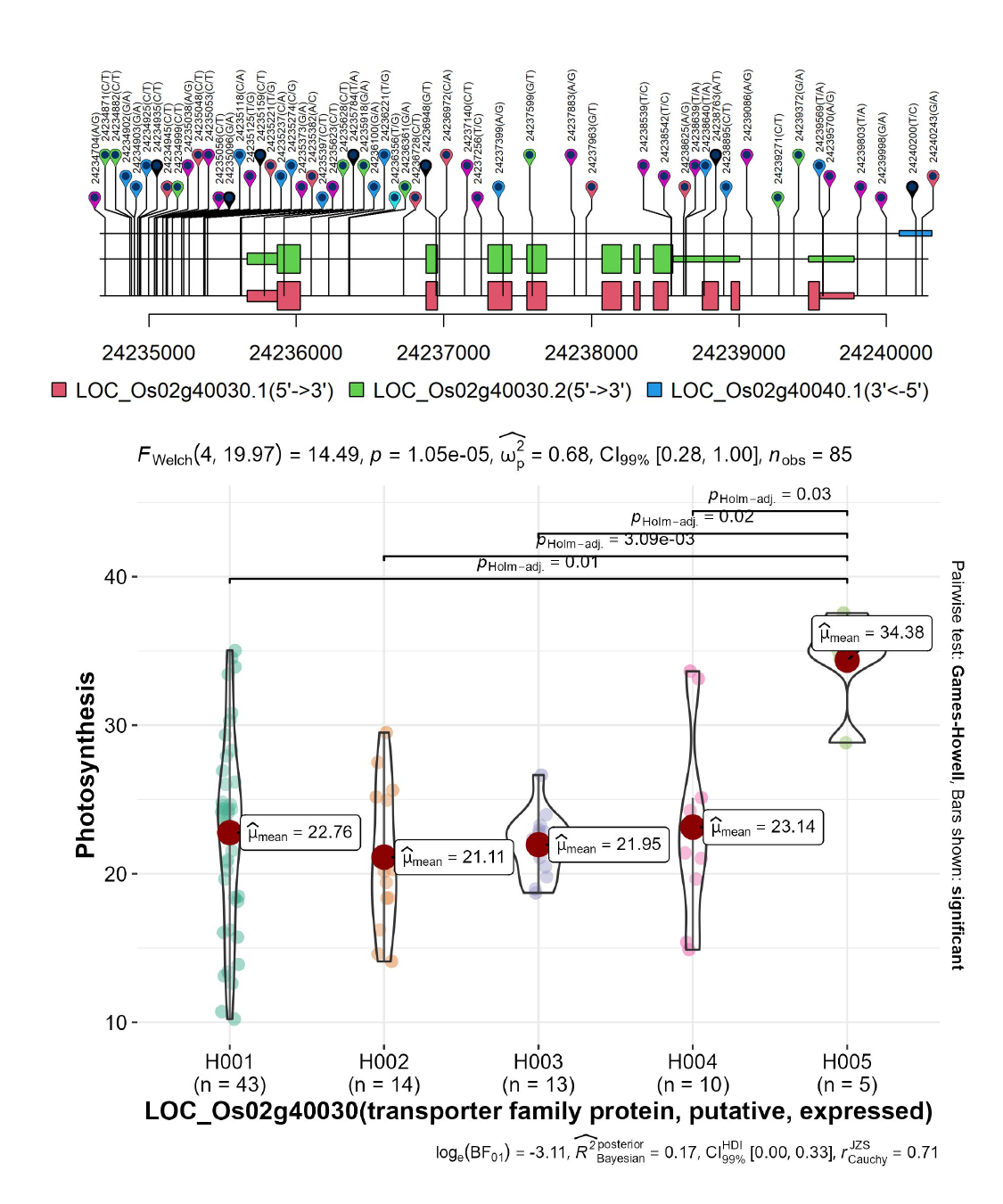

## Slide 16
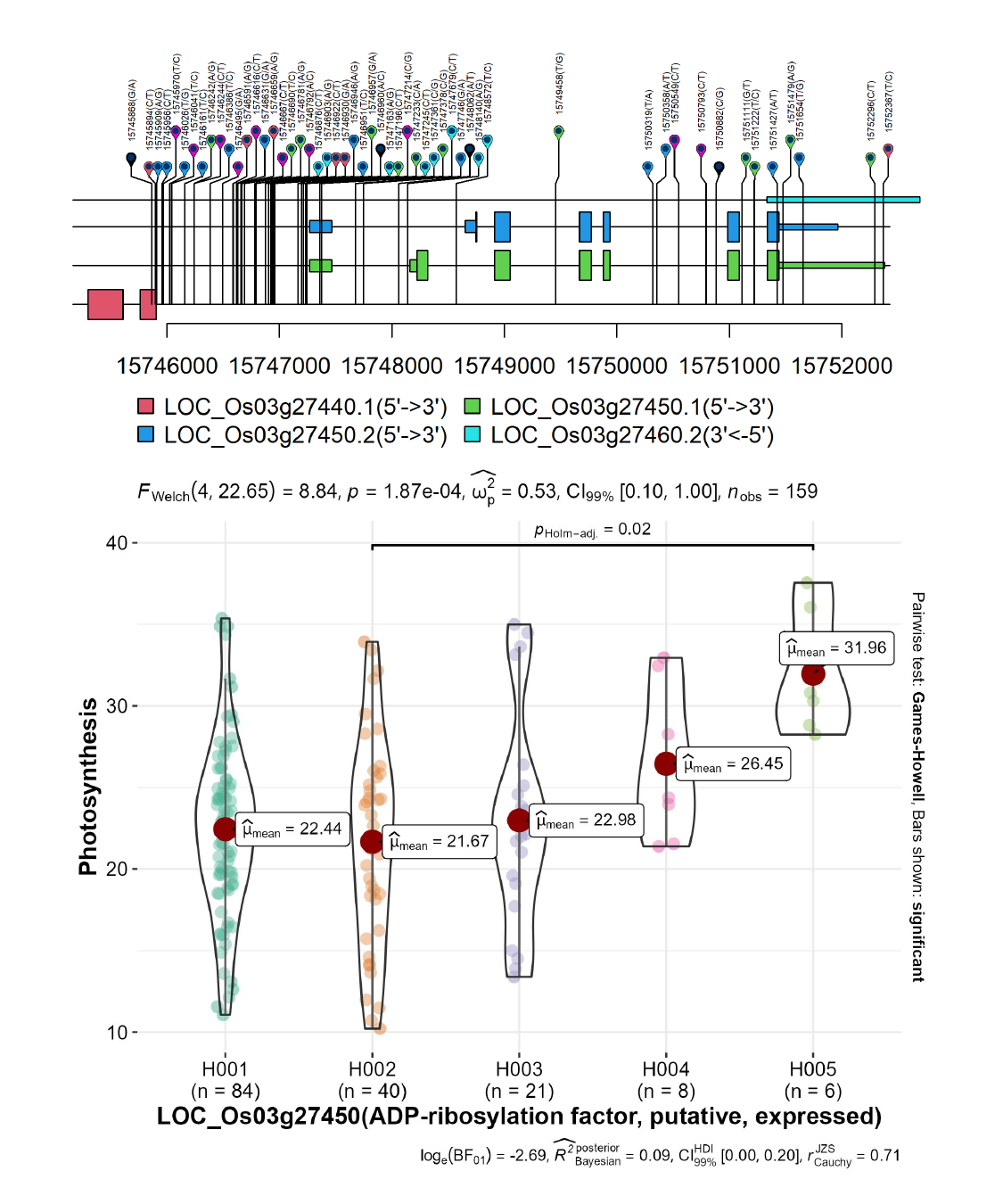

## Slide 17
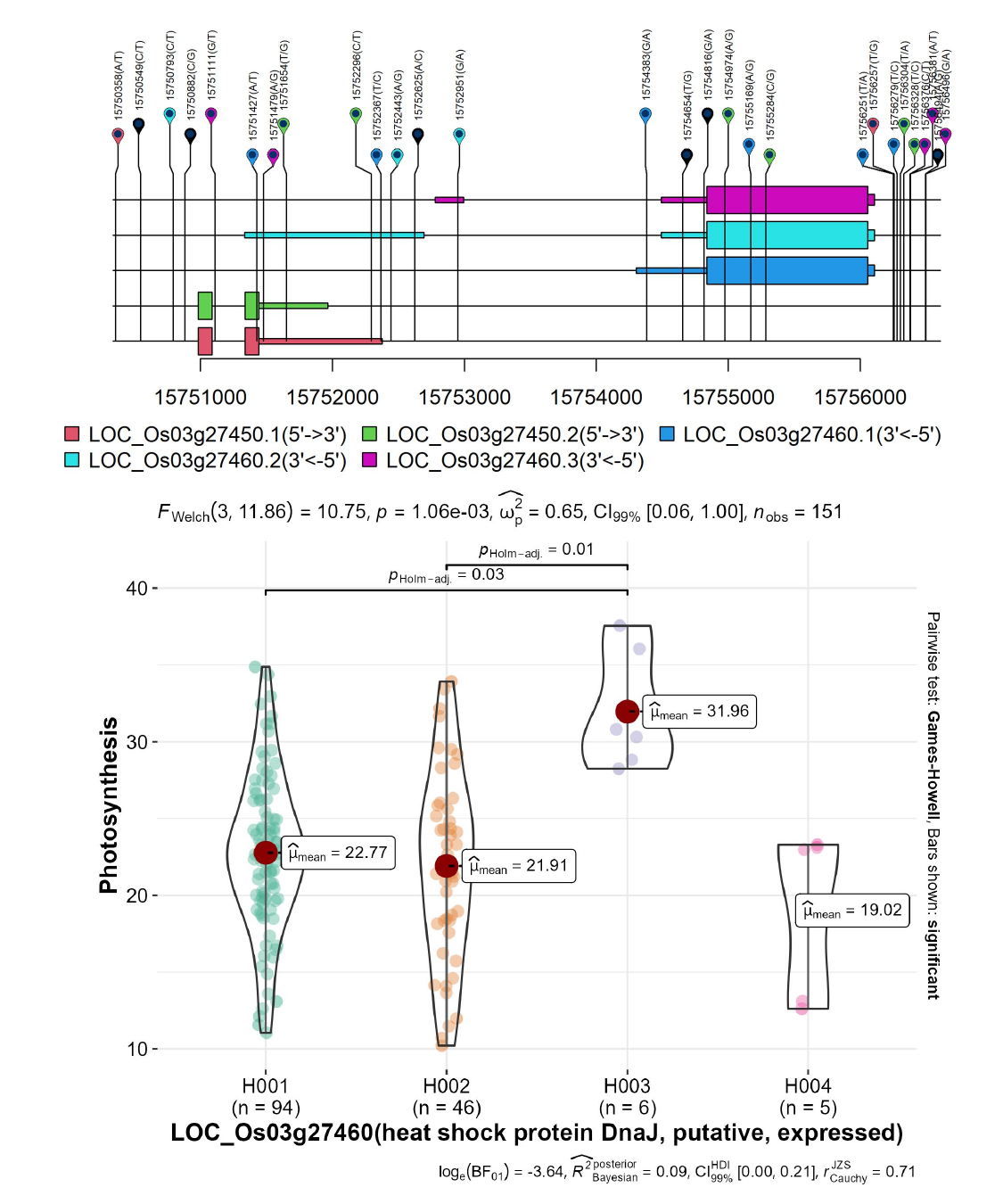

## Slide 18
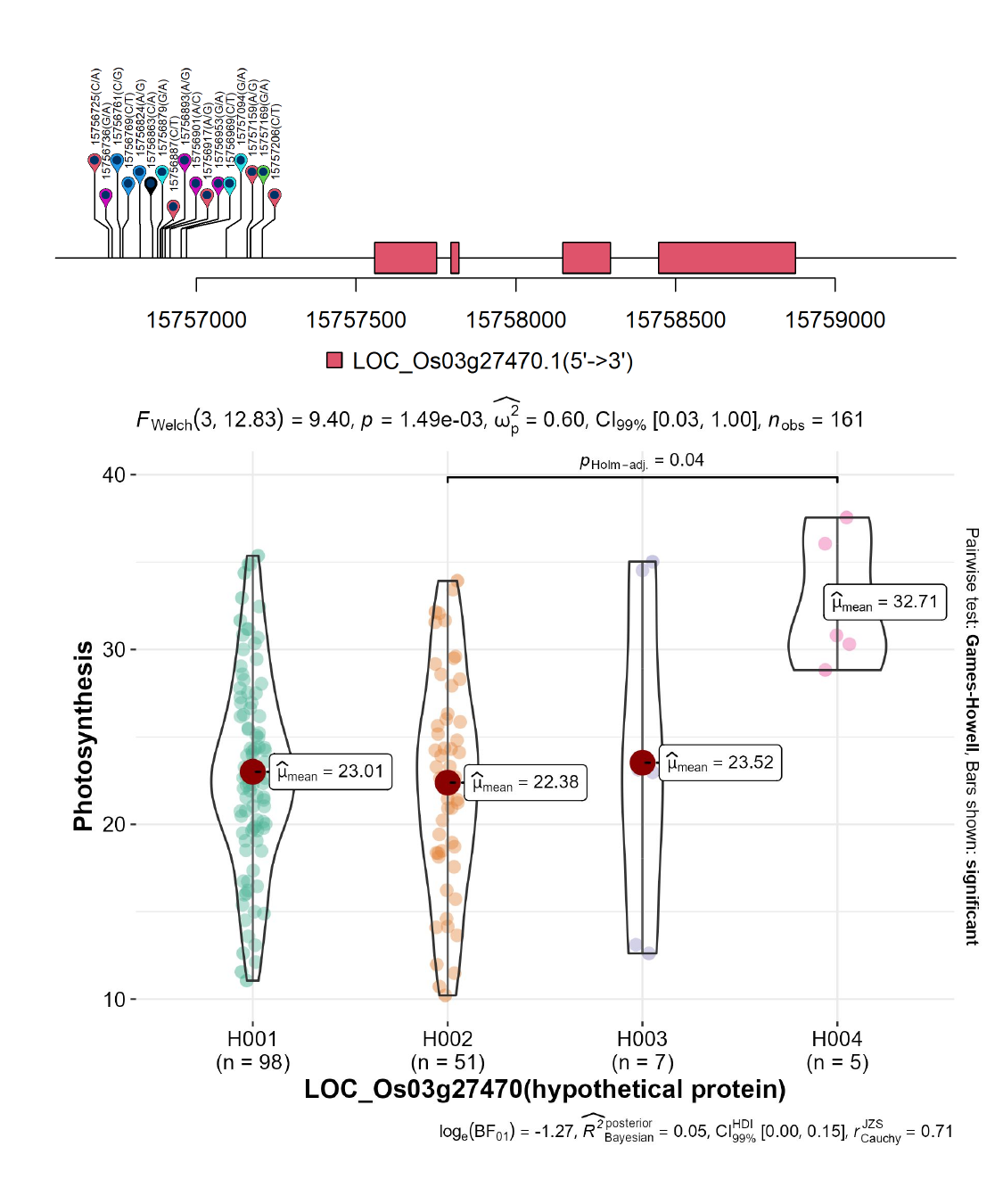

## Slide 19
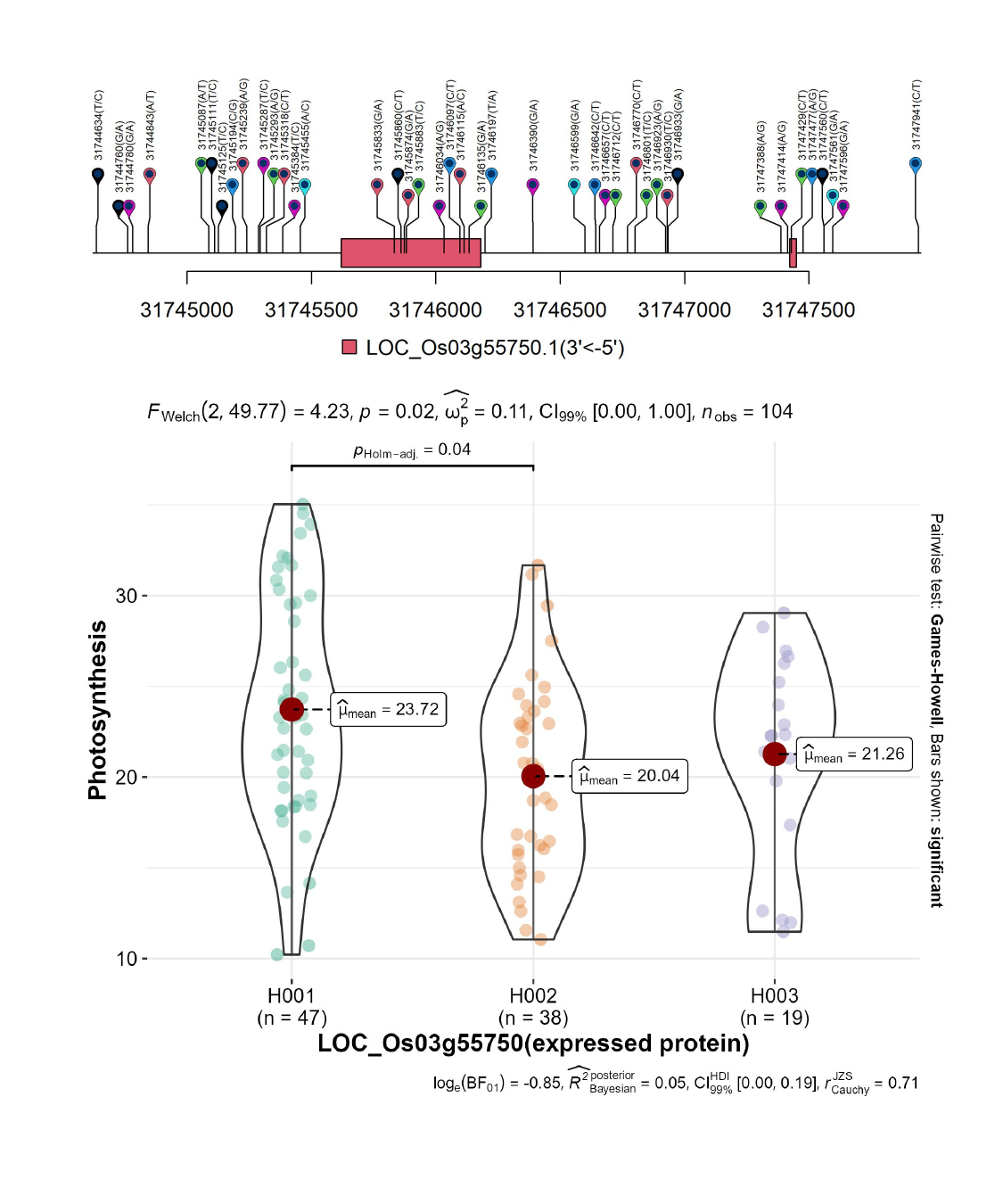

## Slide 20
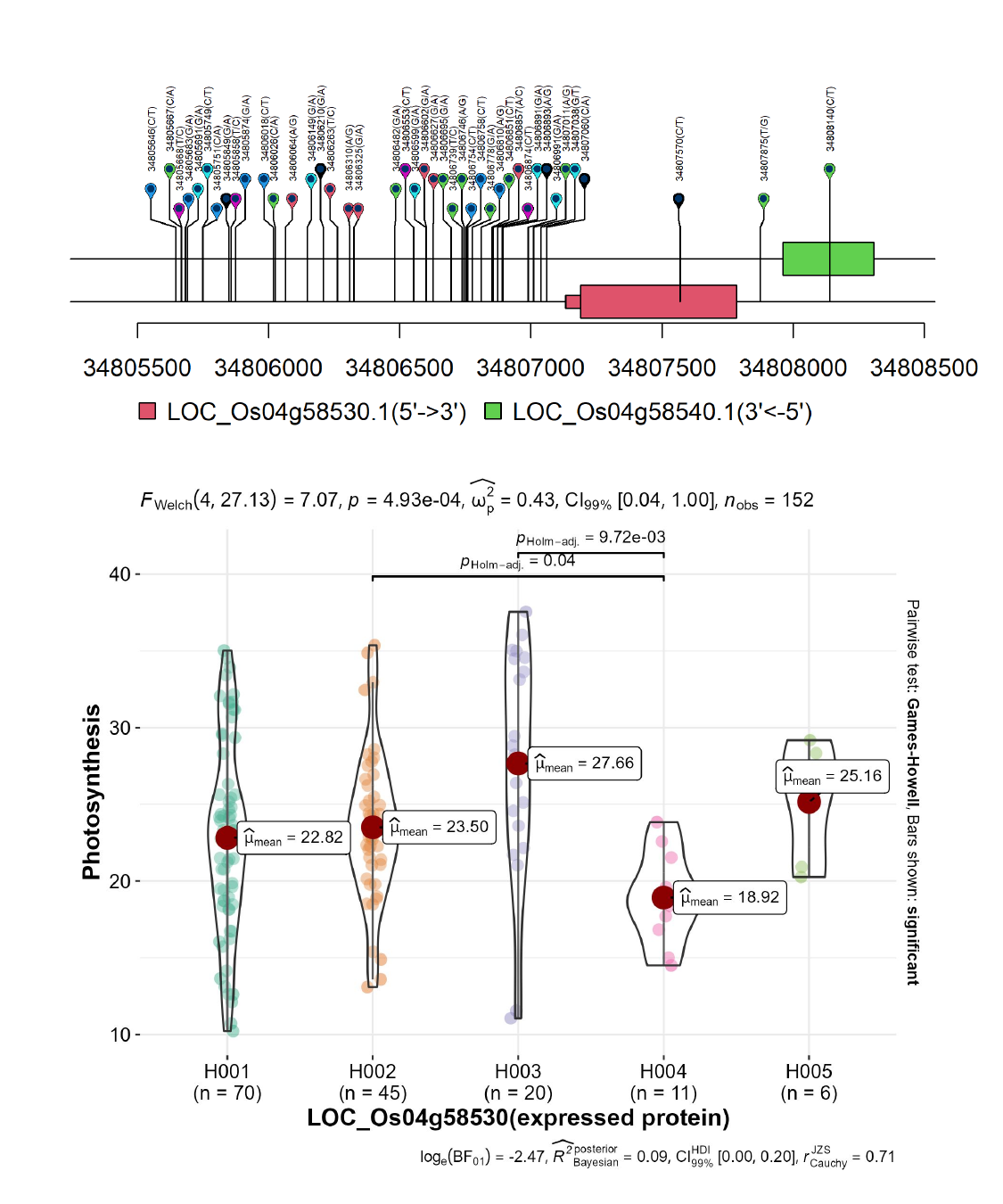

## Slide 21
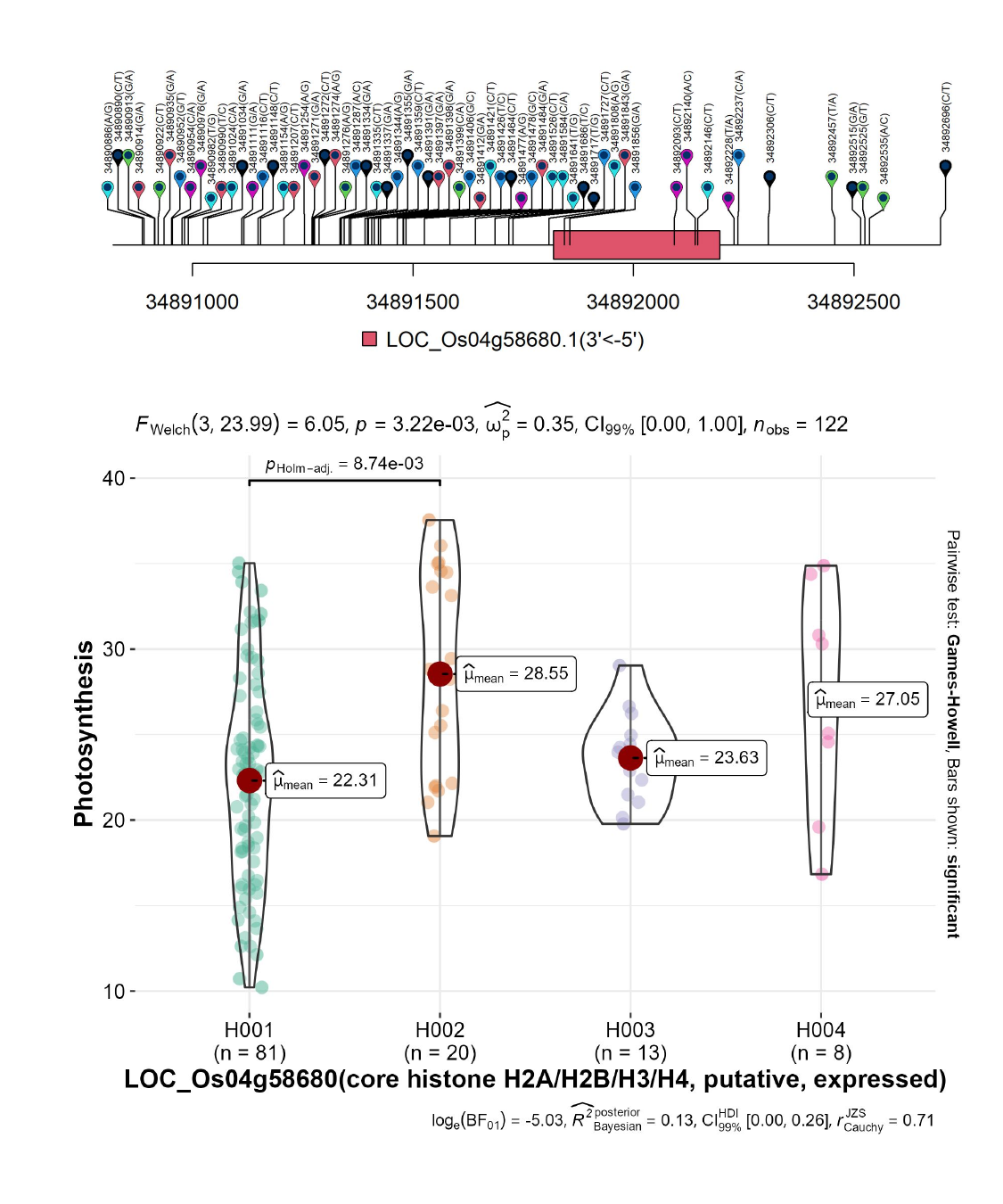

## Slide 22
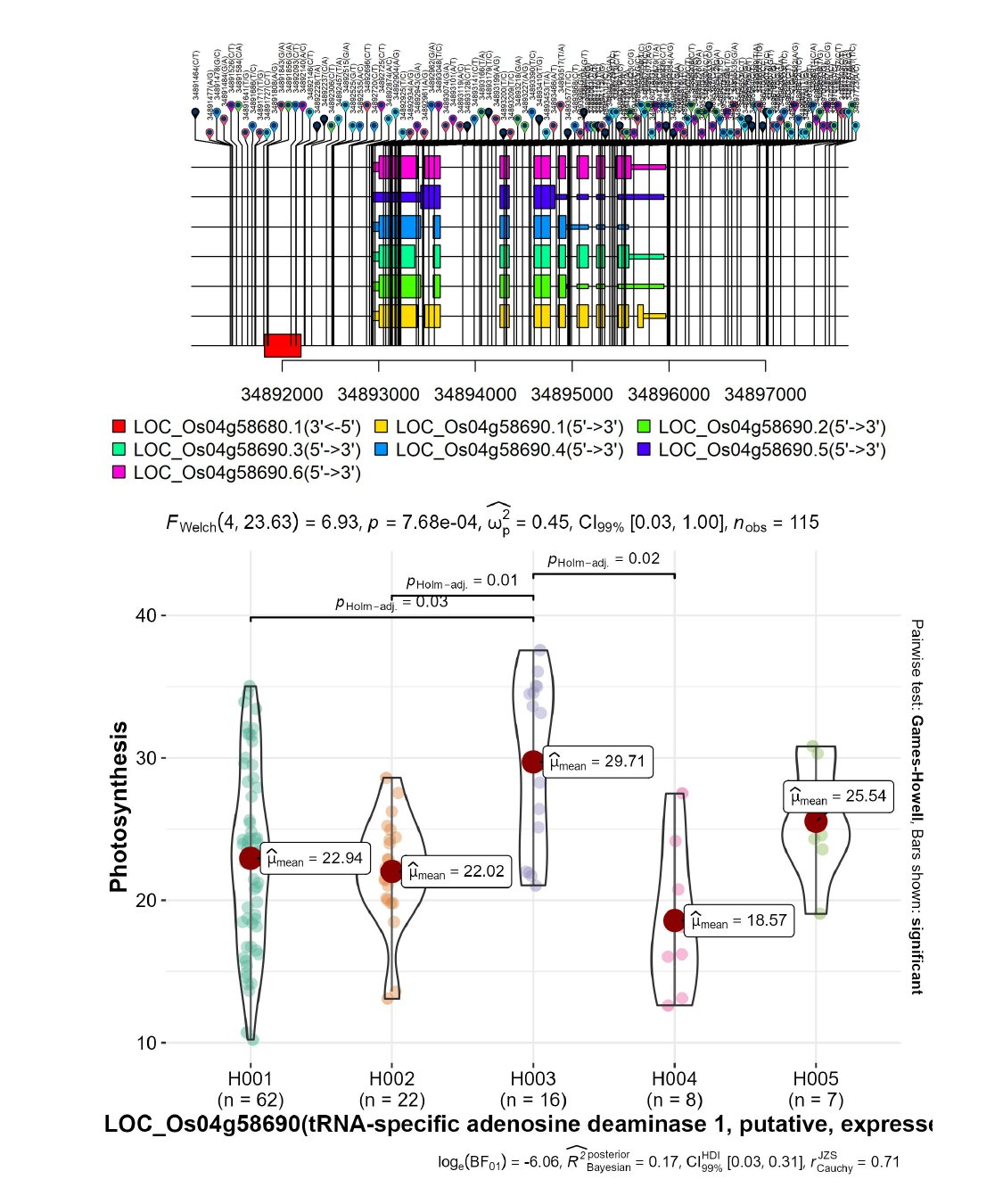

## Slide 23
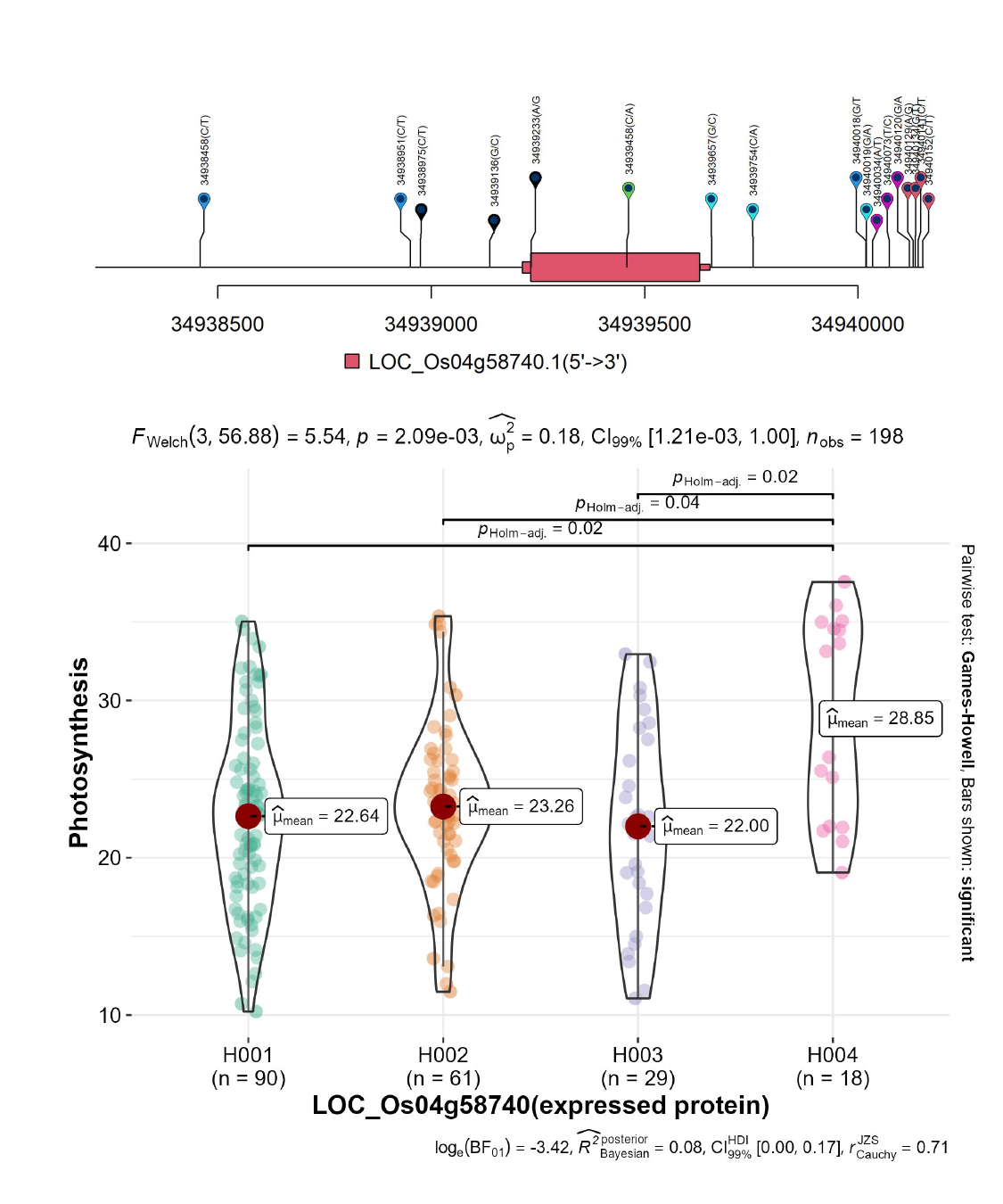

## Slide 24
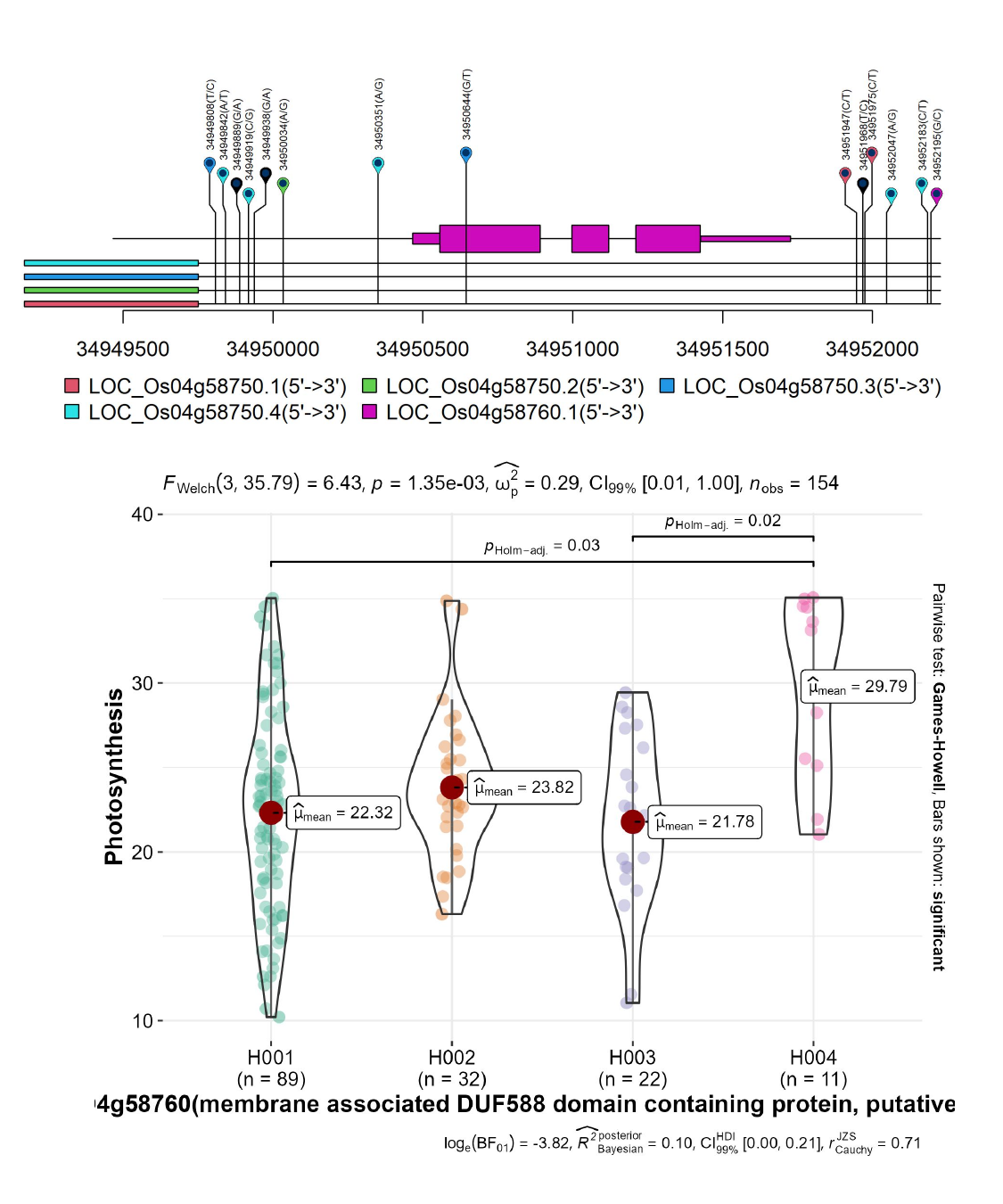

## Slide 25
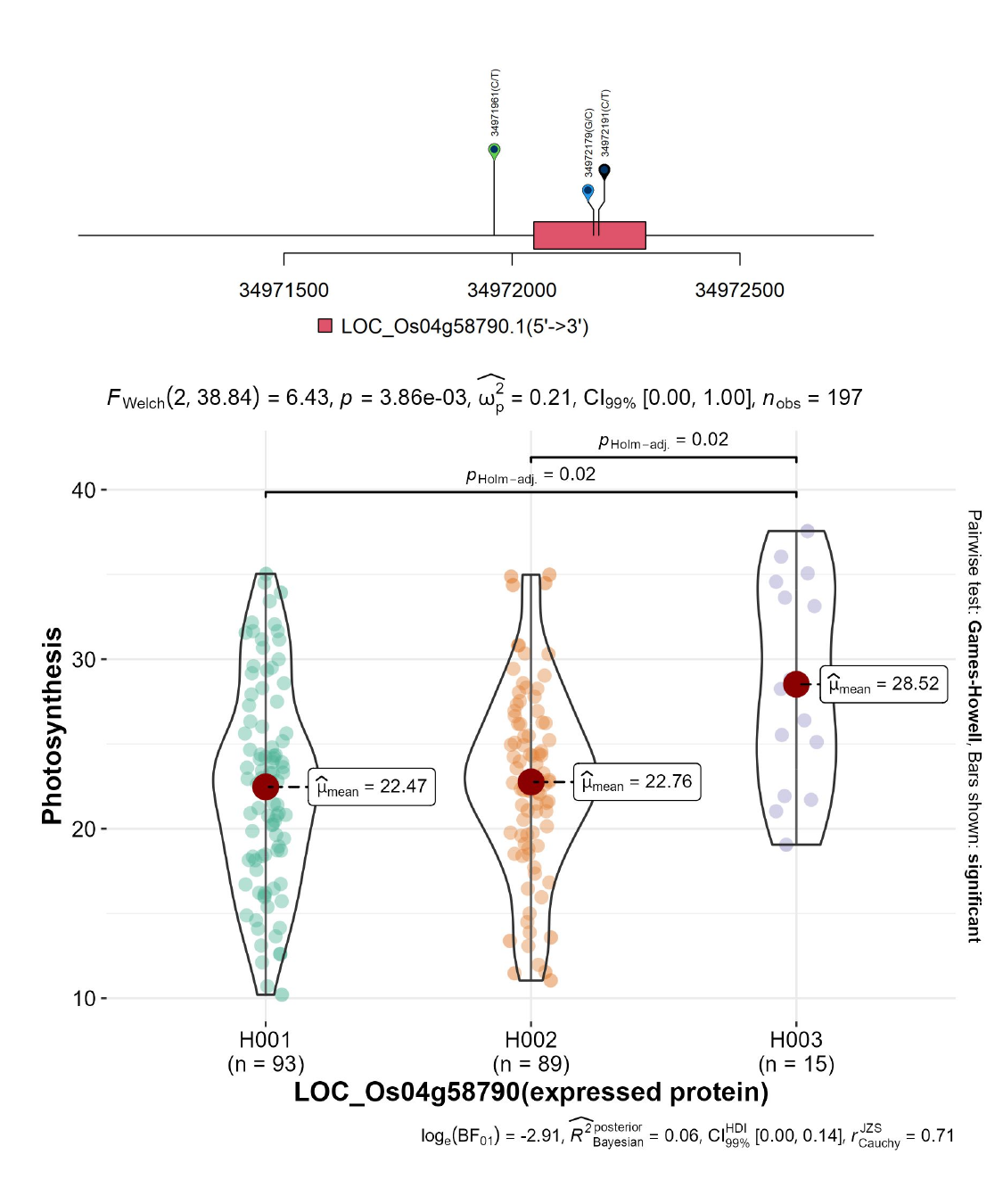

## Slide 26
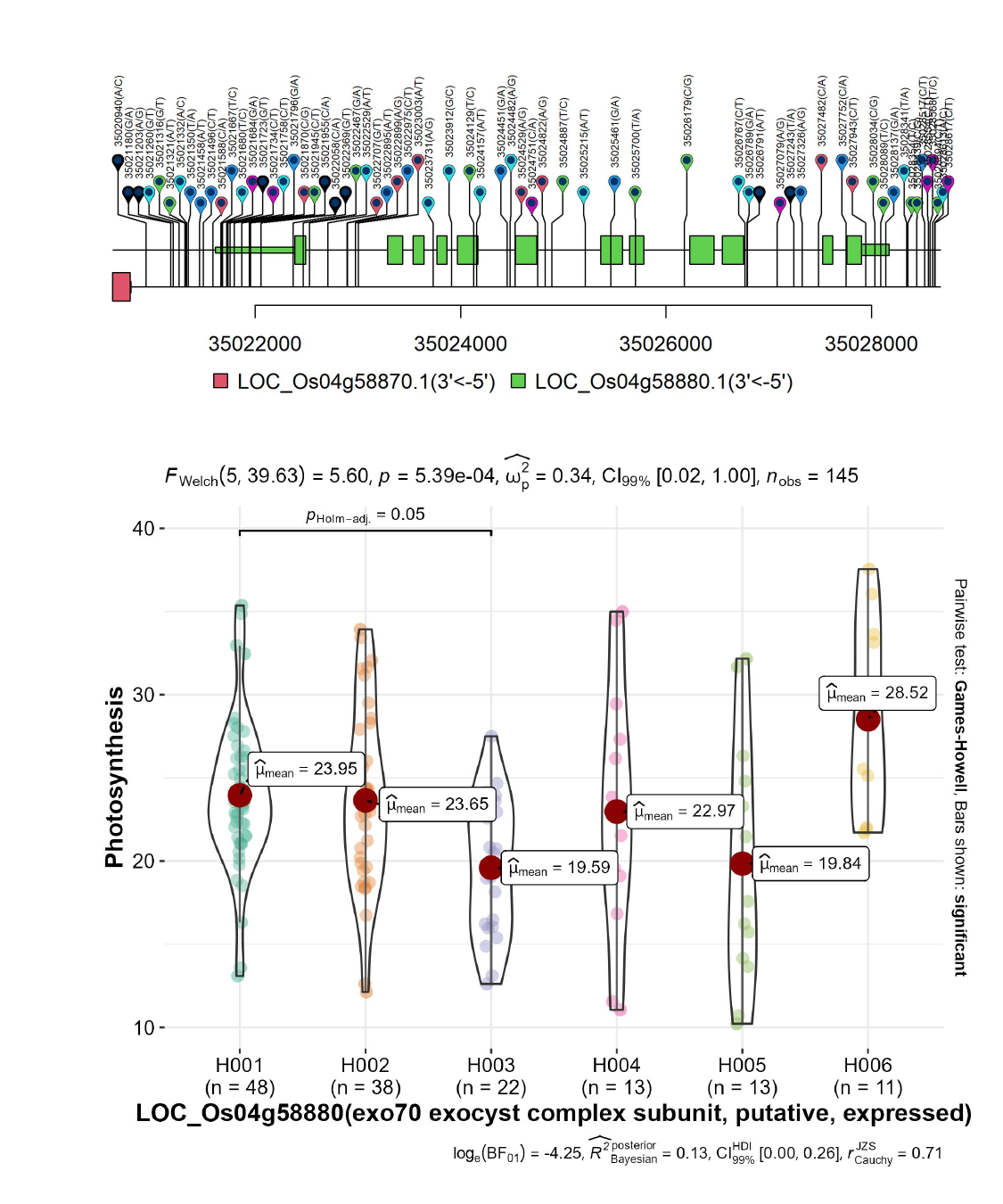

## Slide 27
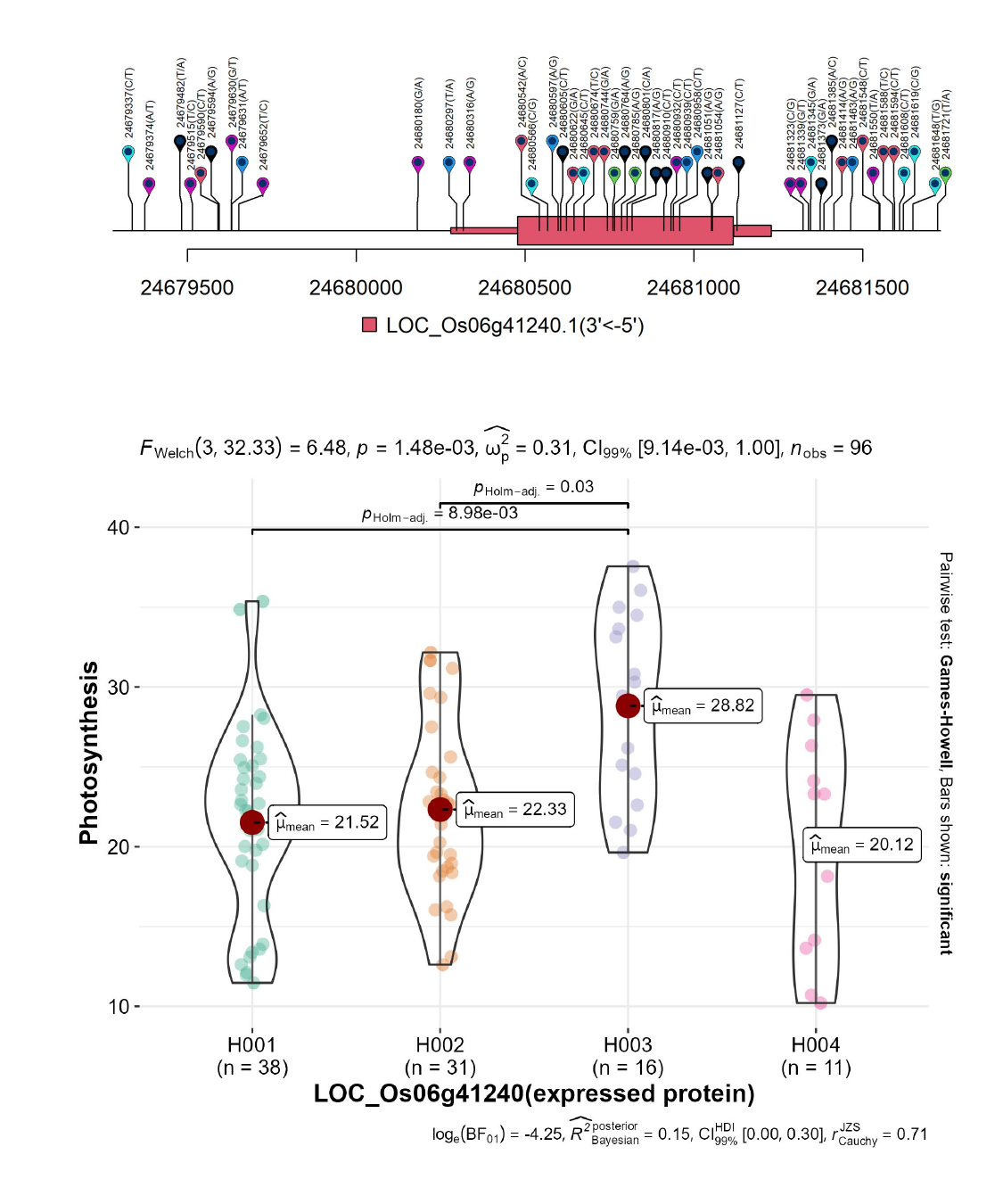

## Slide 28
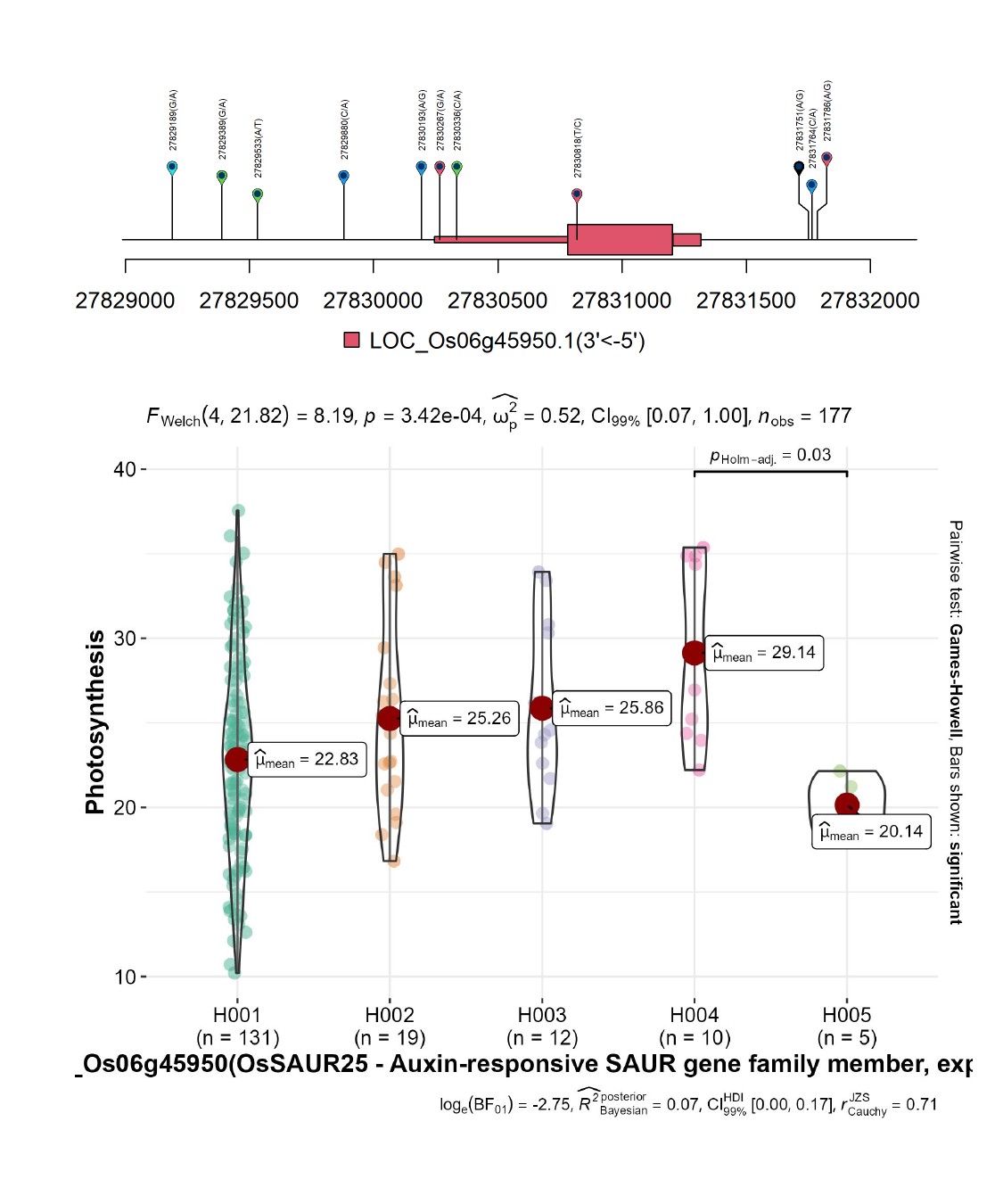

## Slide 29
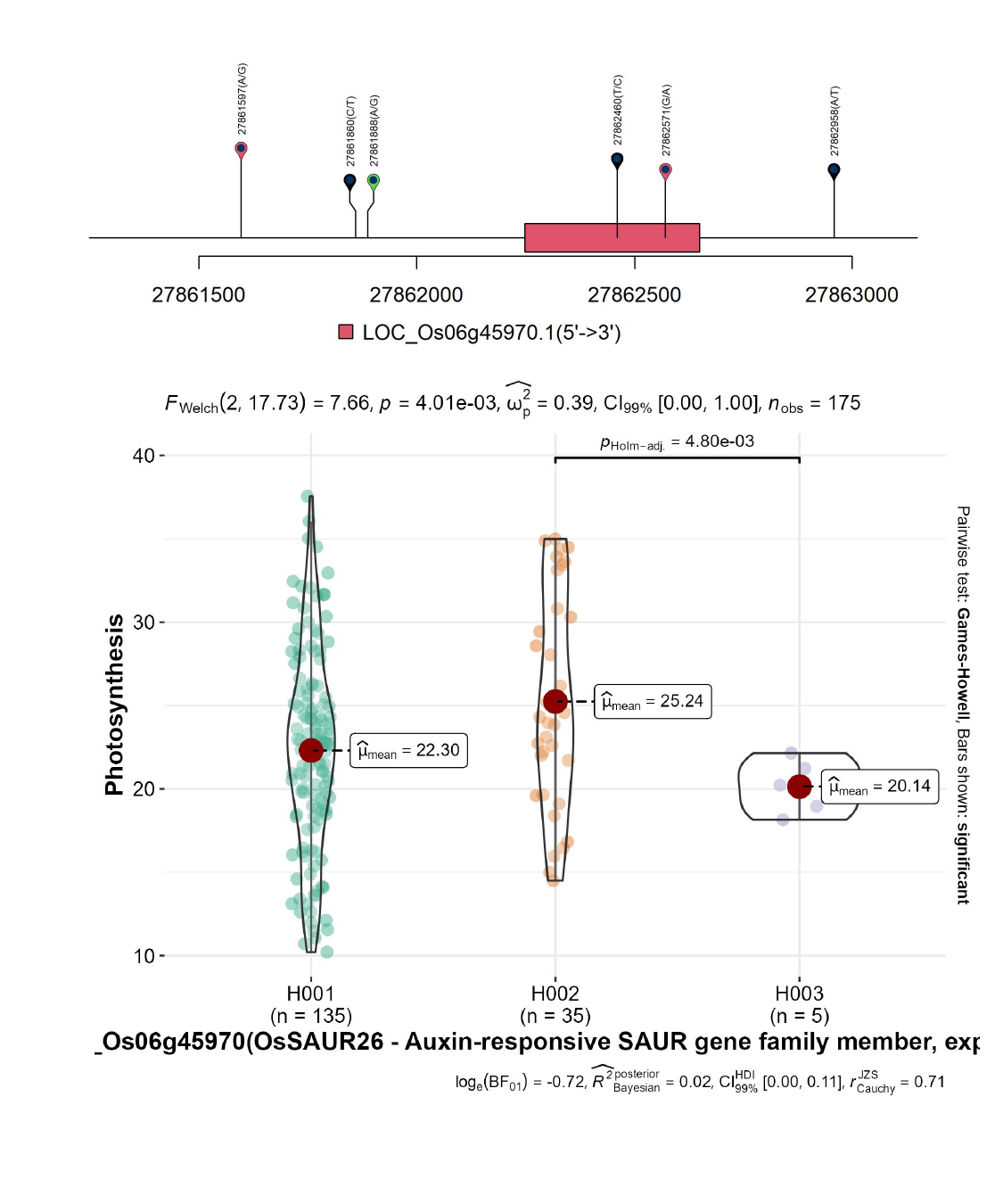

## Slide 30
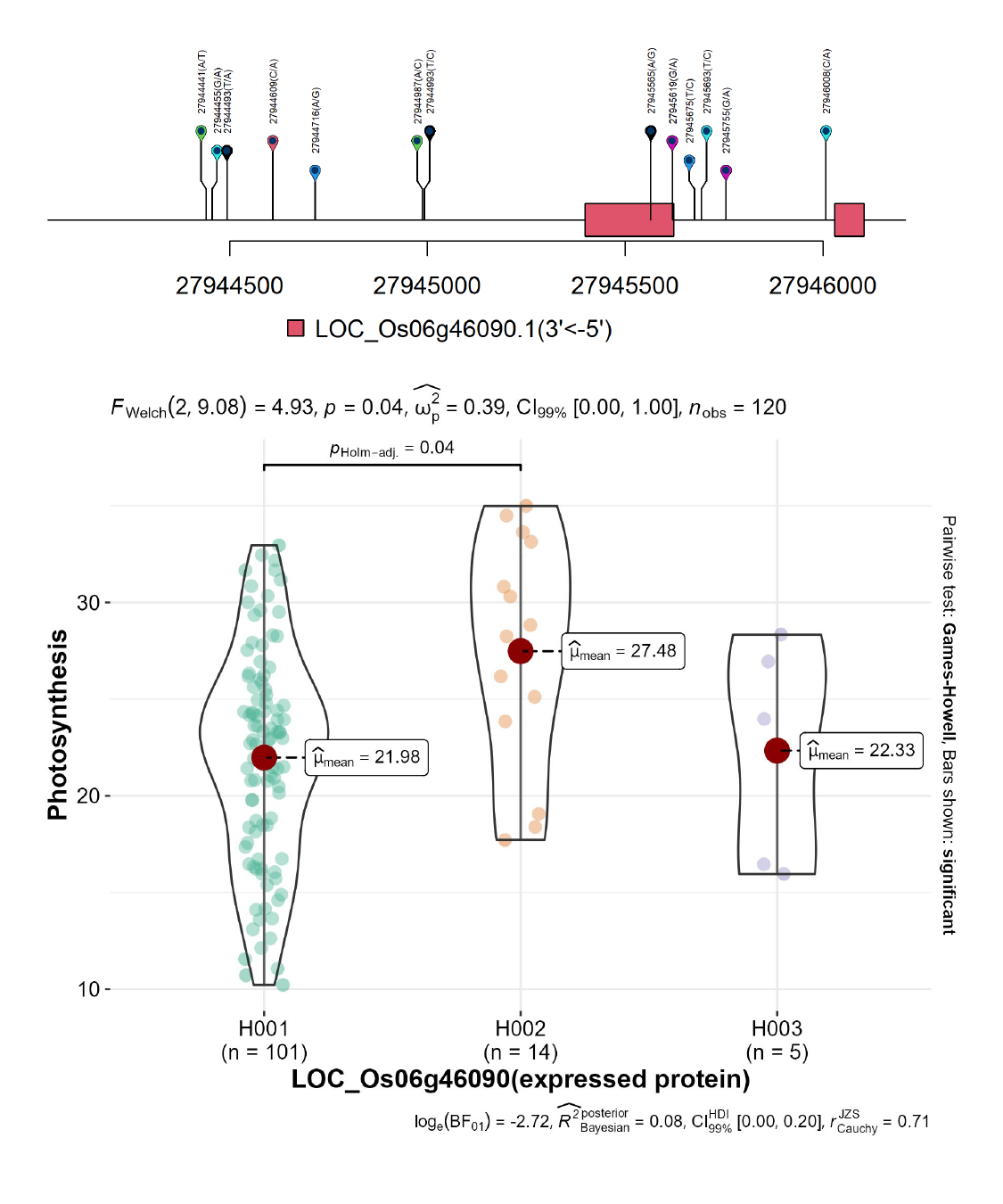

## Slide 31
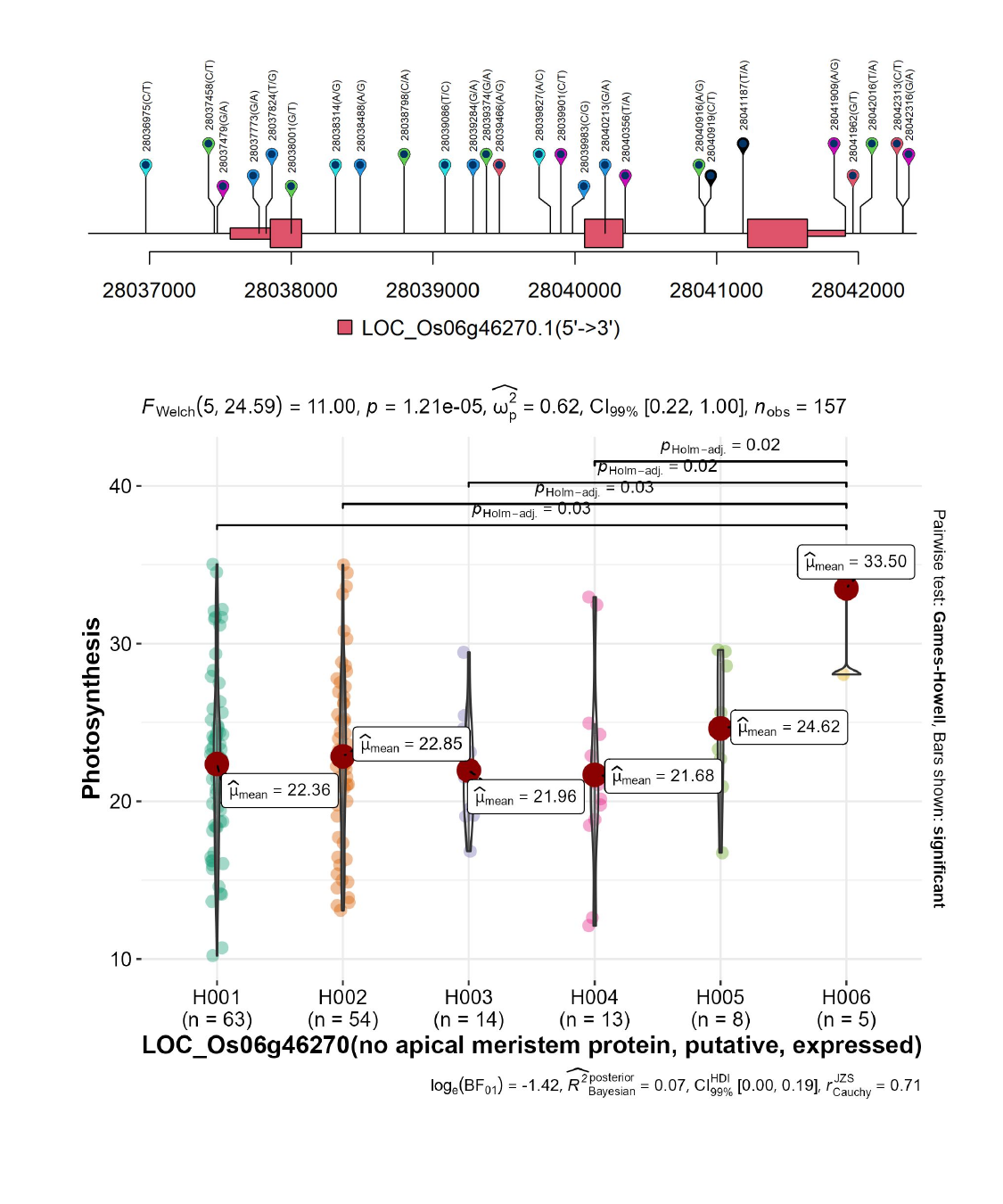

## Slide 32
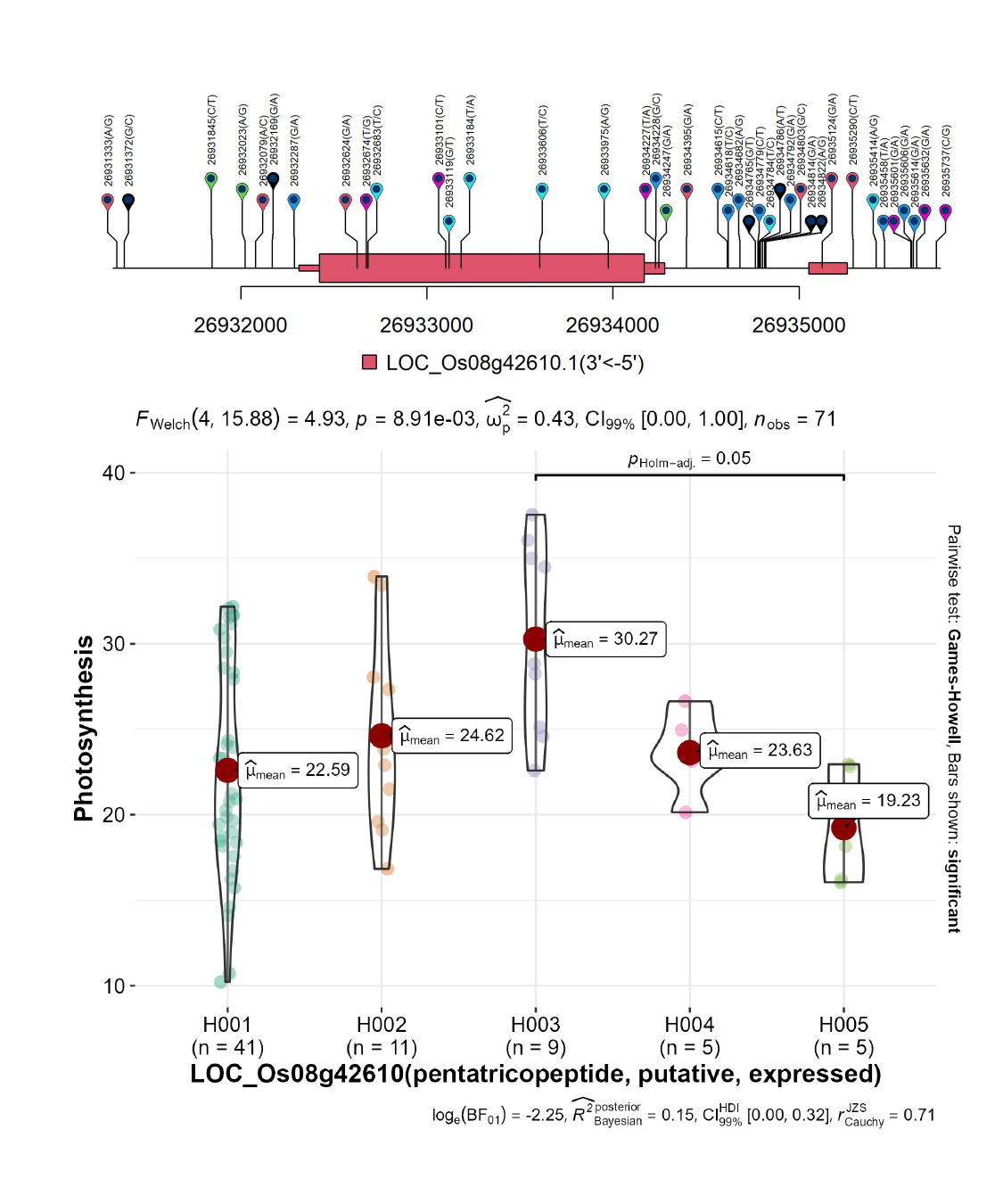

## Slide 33
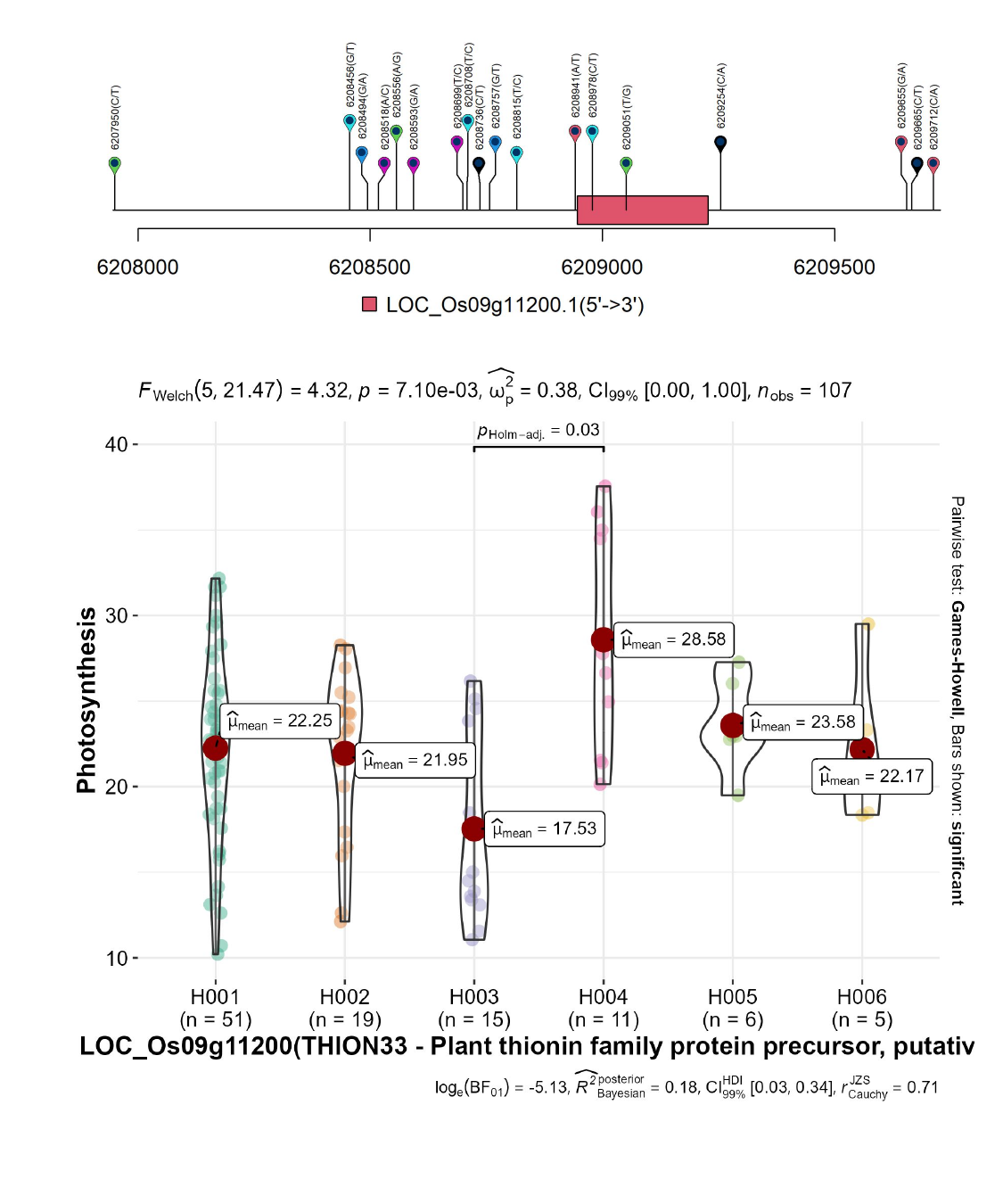

## Slide 34
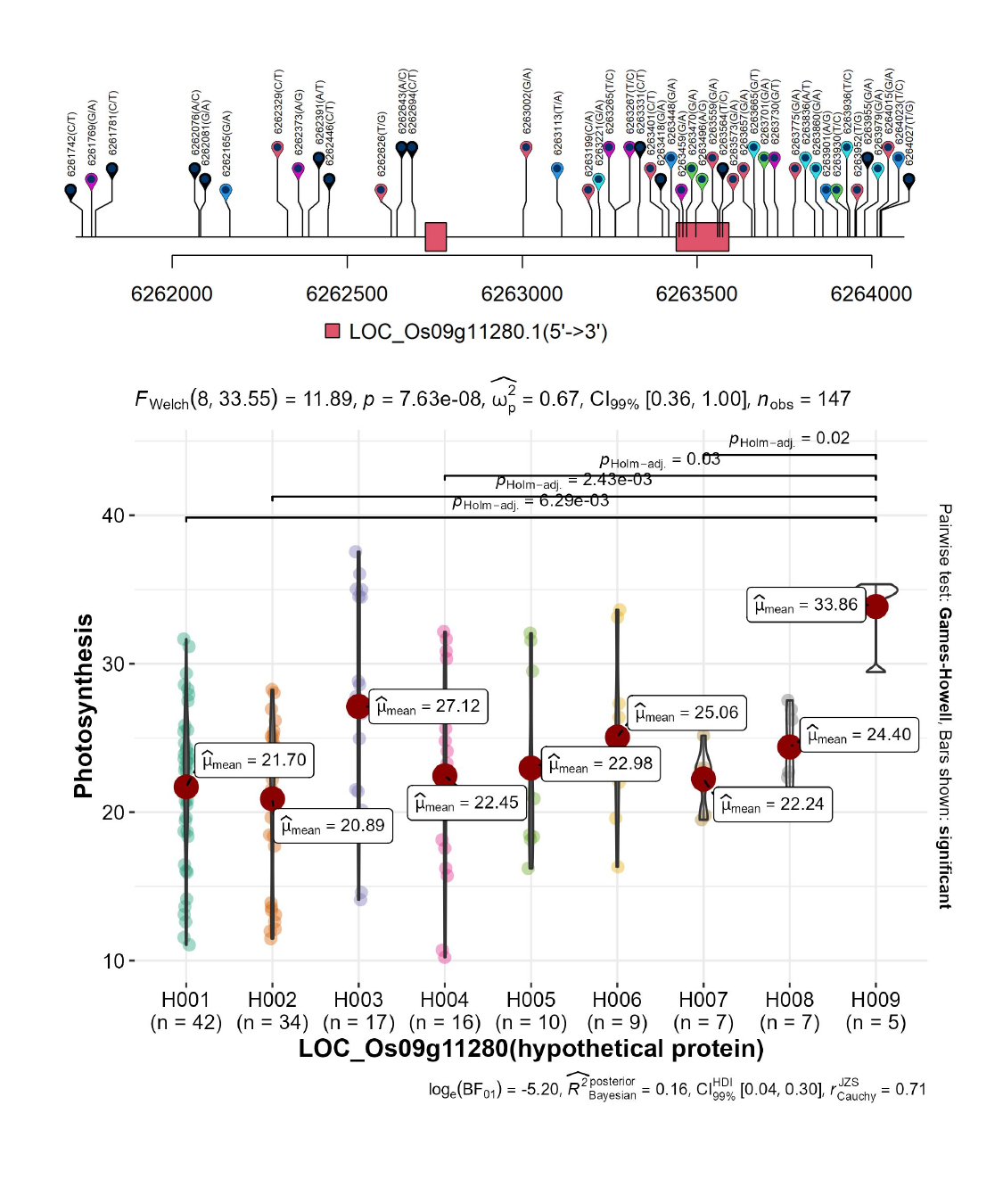

## Slide 35
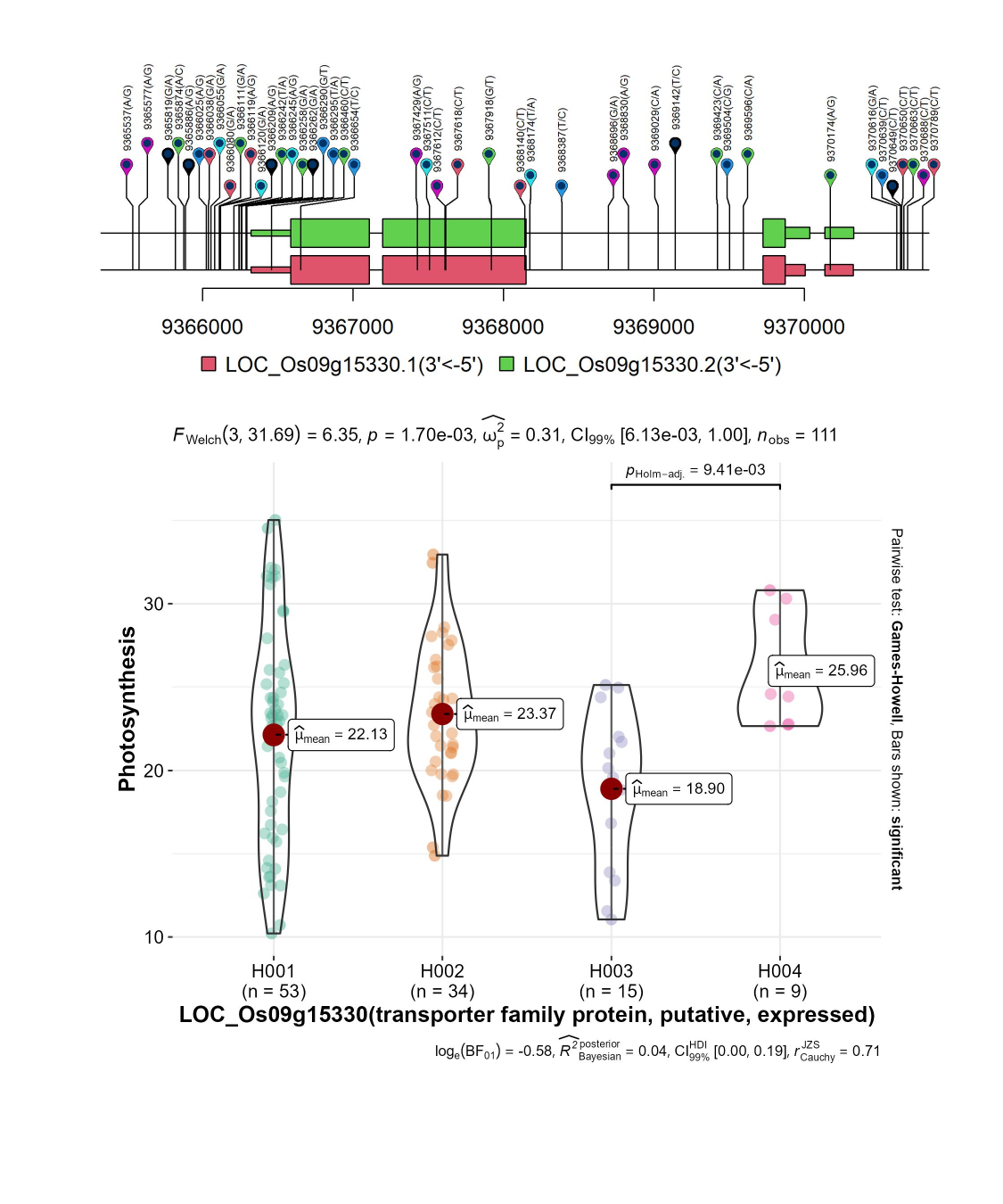

## Slide 36
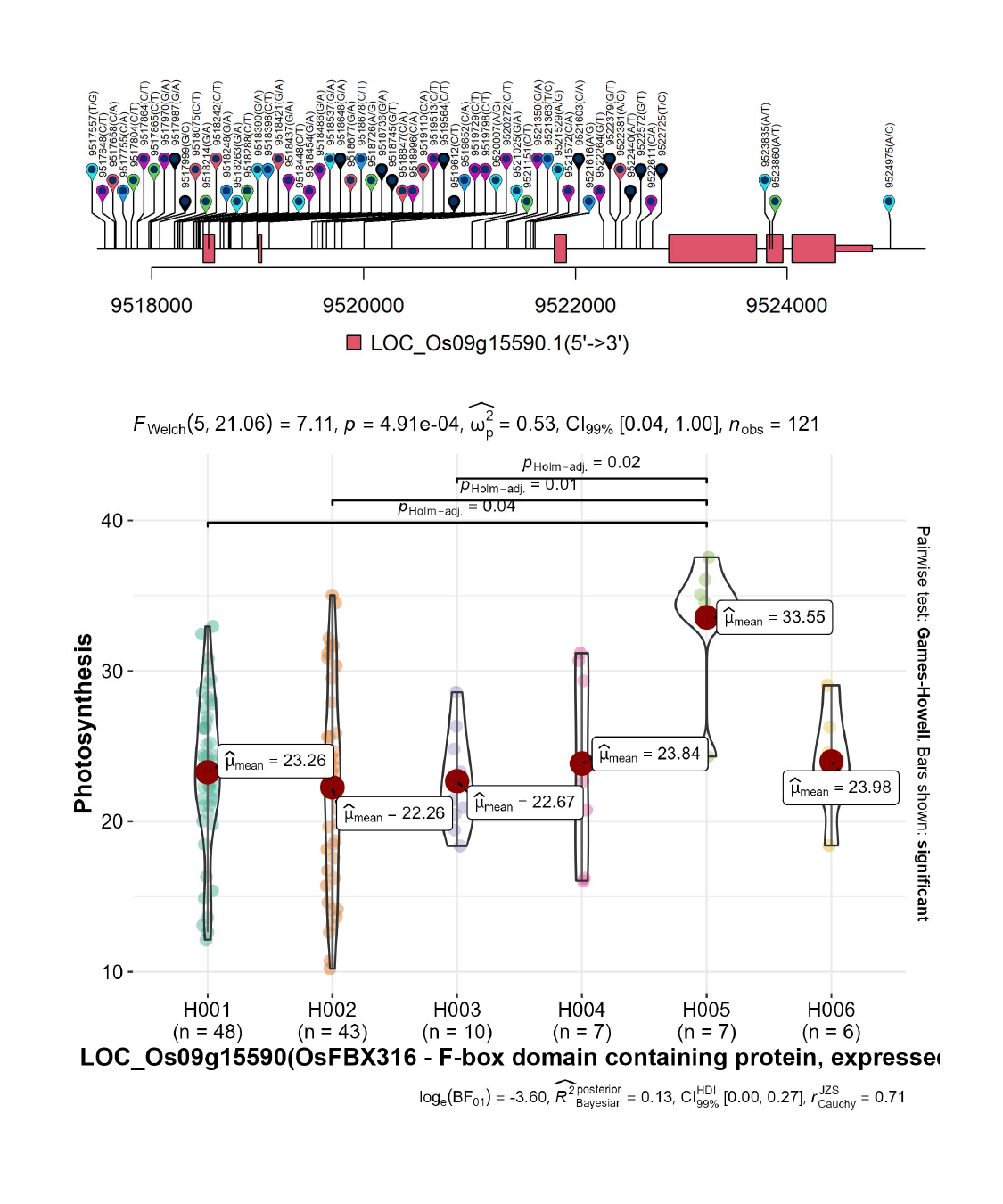

## Slide 37
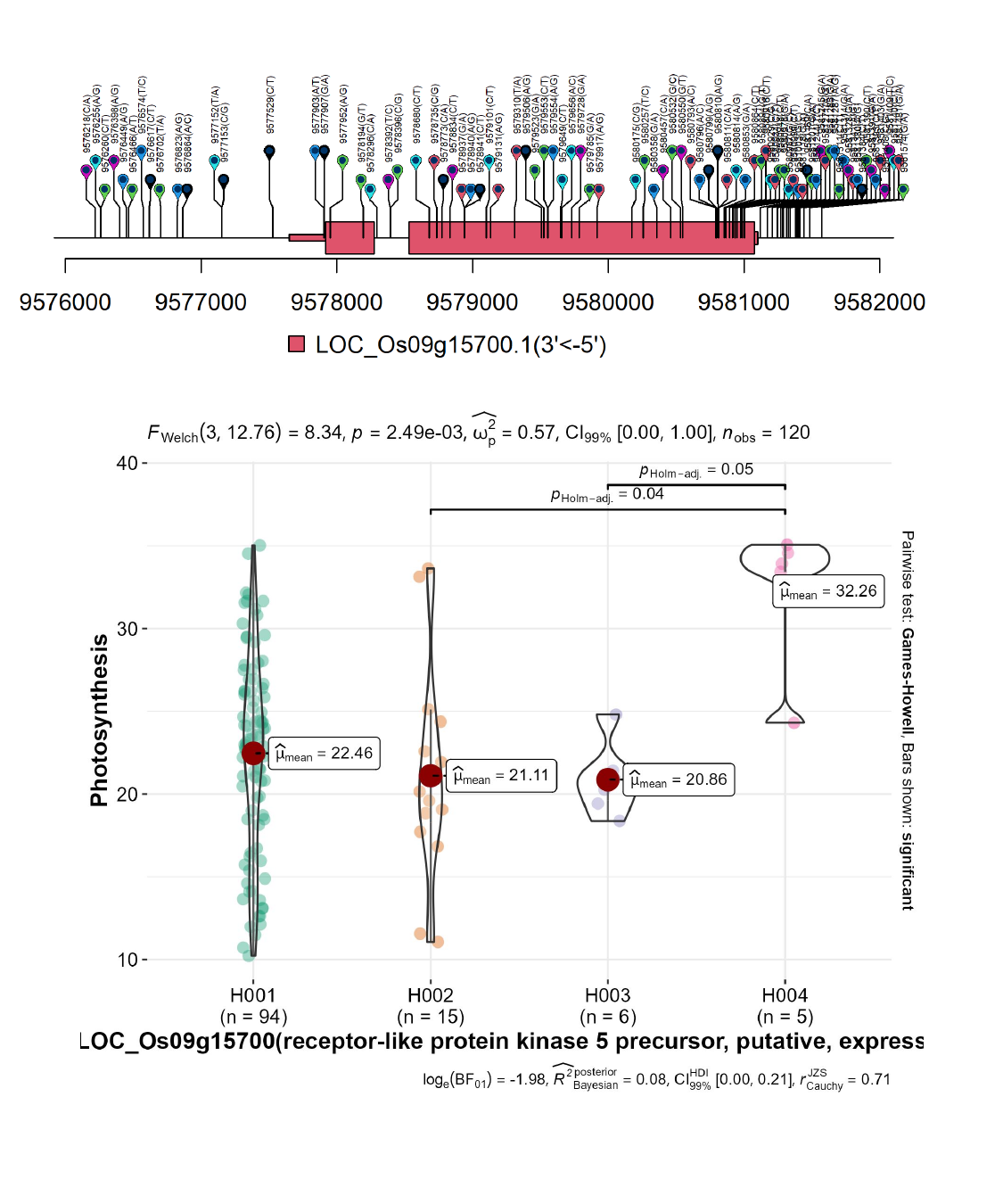

## Slide 38
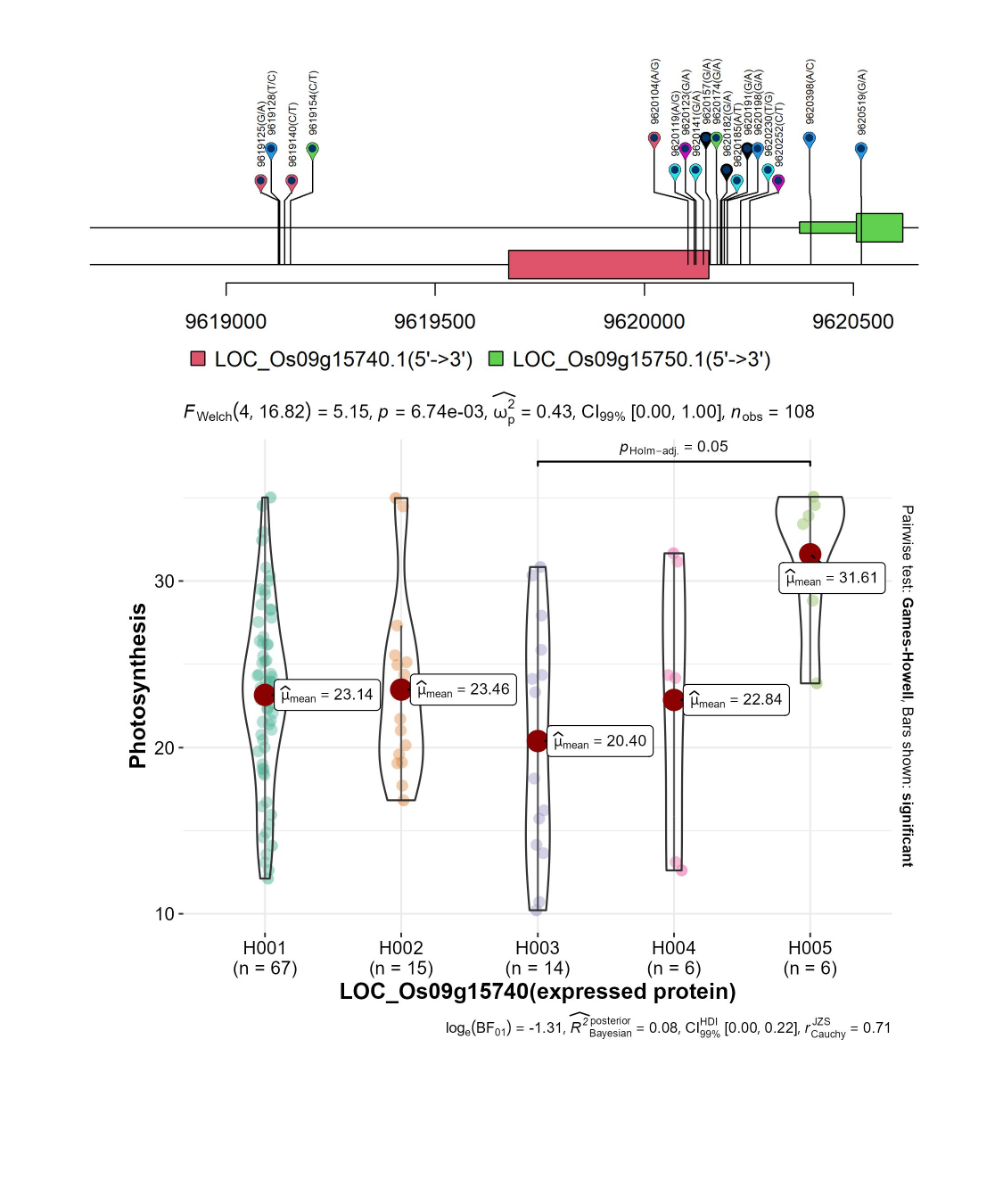

## Slide 39
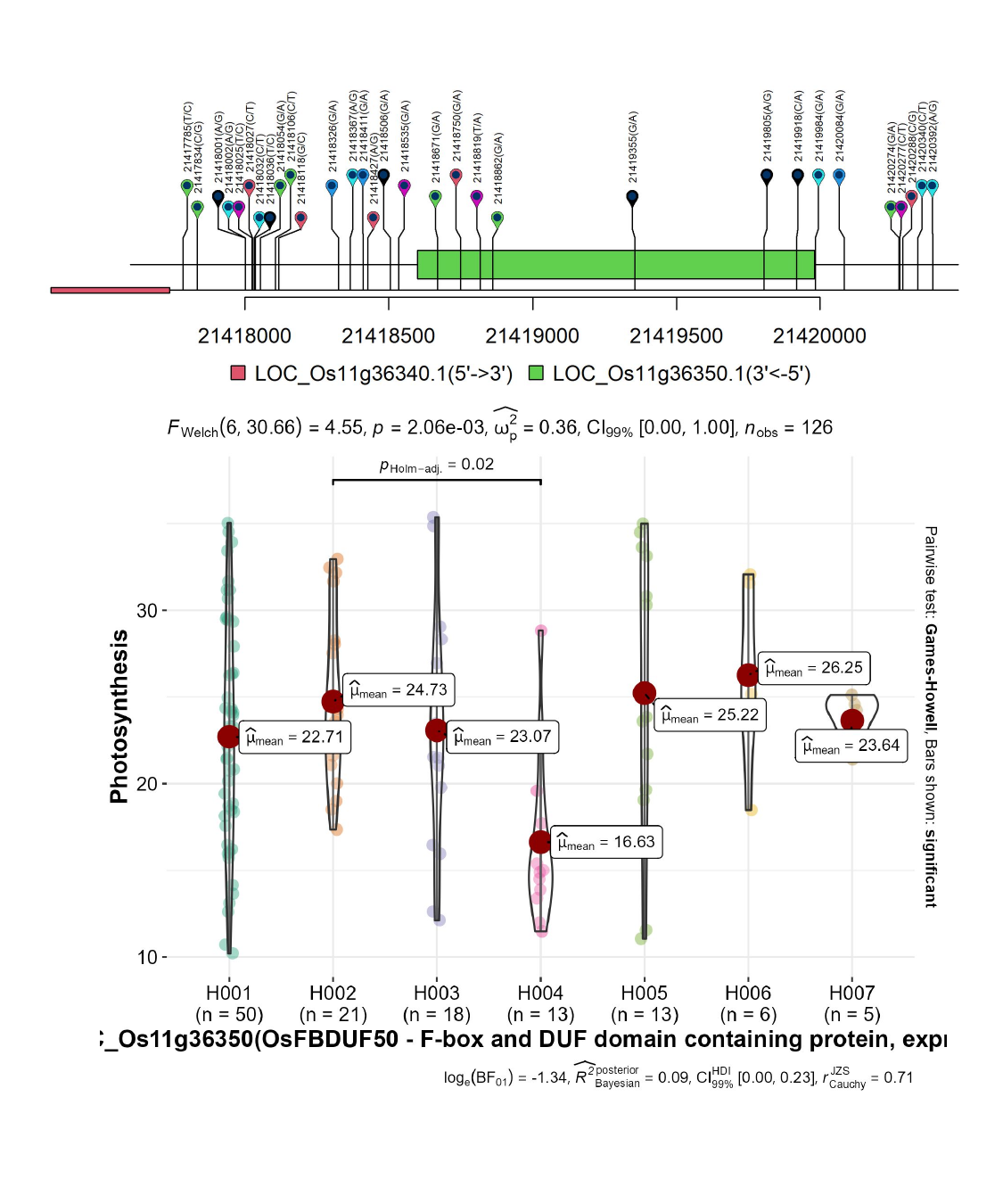

## Slide 40
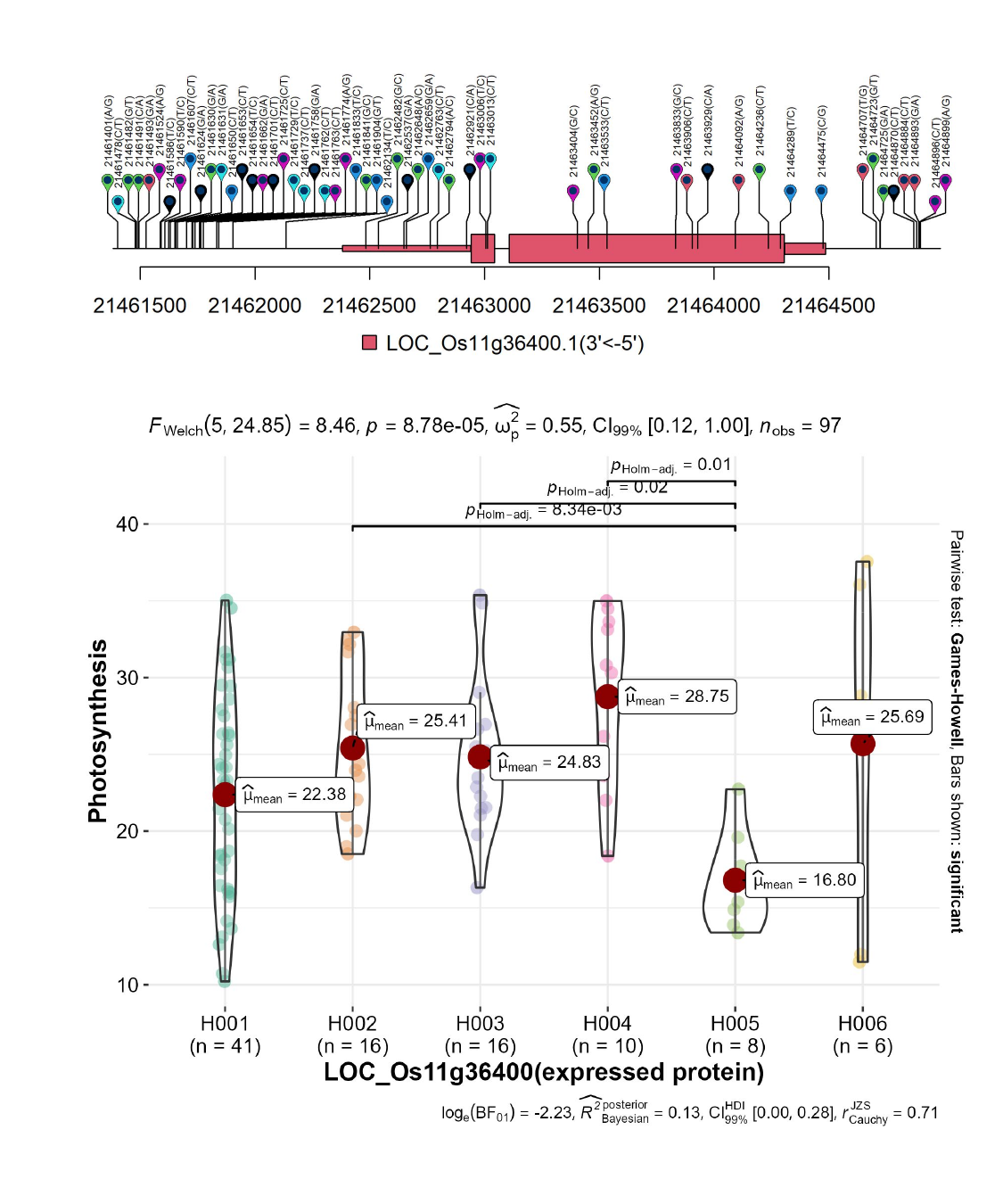

## Slide 41
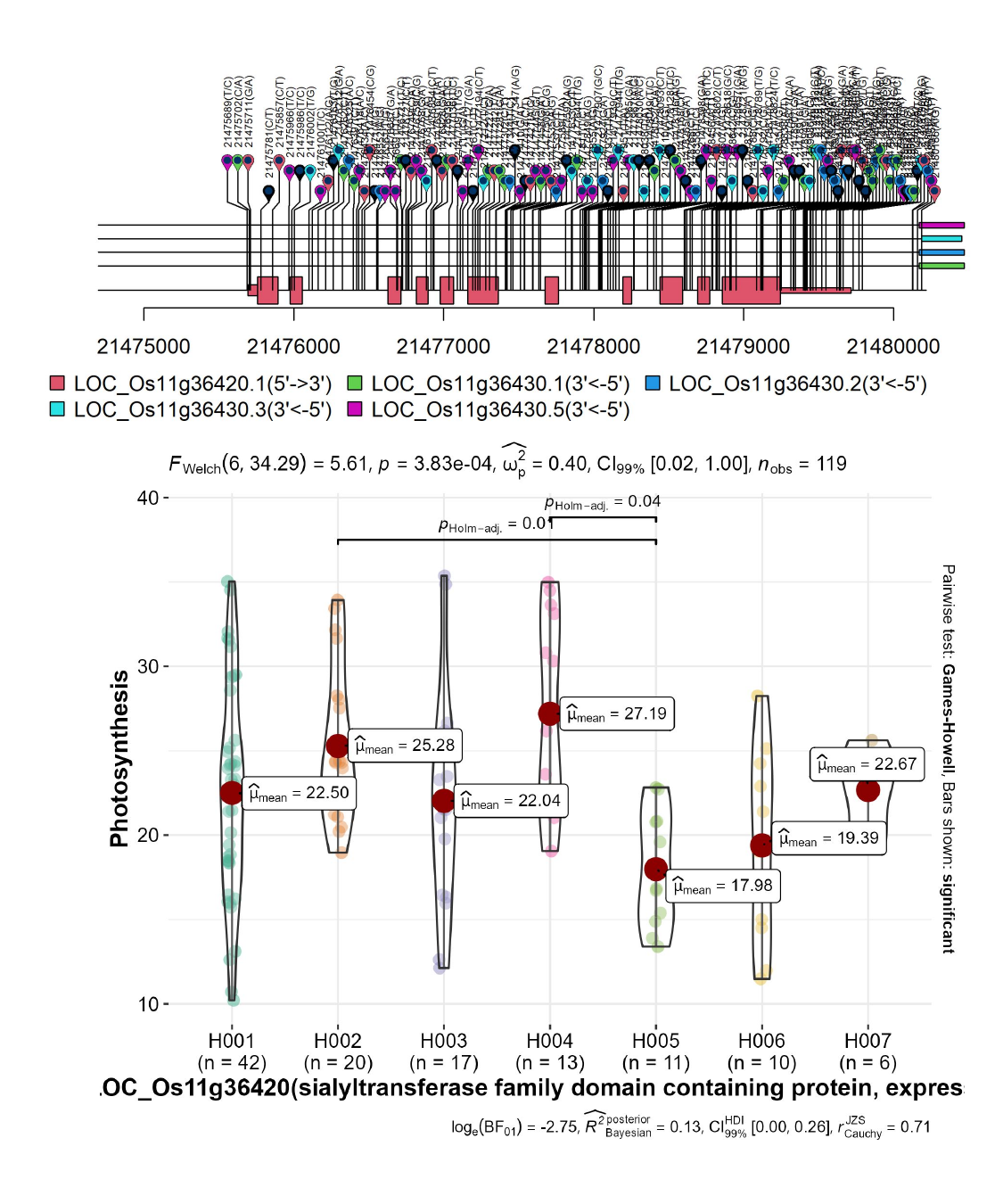

## Slide 42
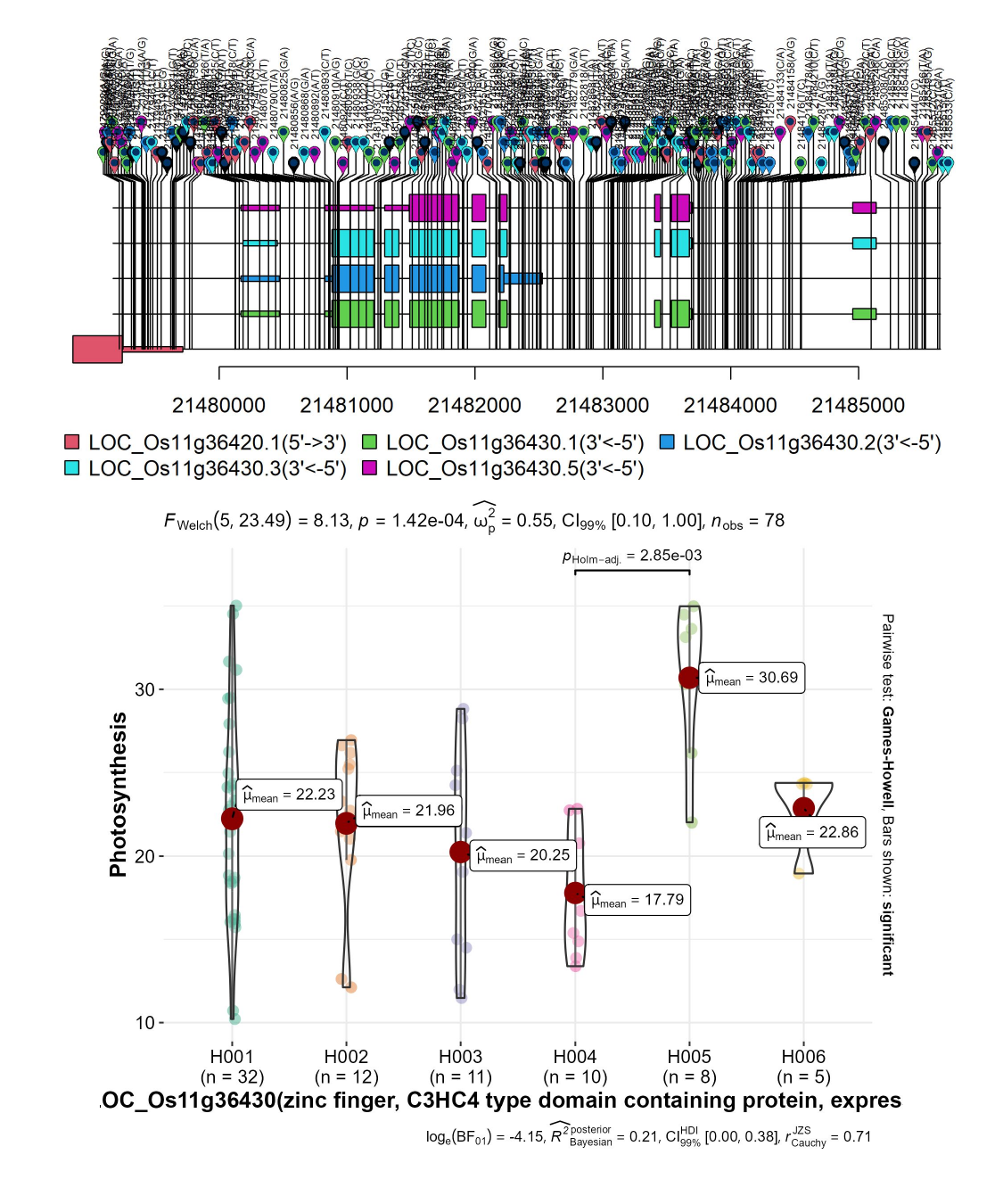

## Slide 43
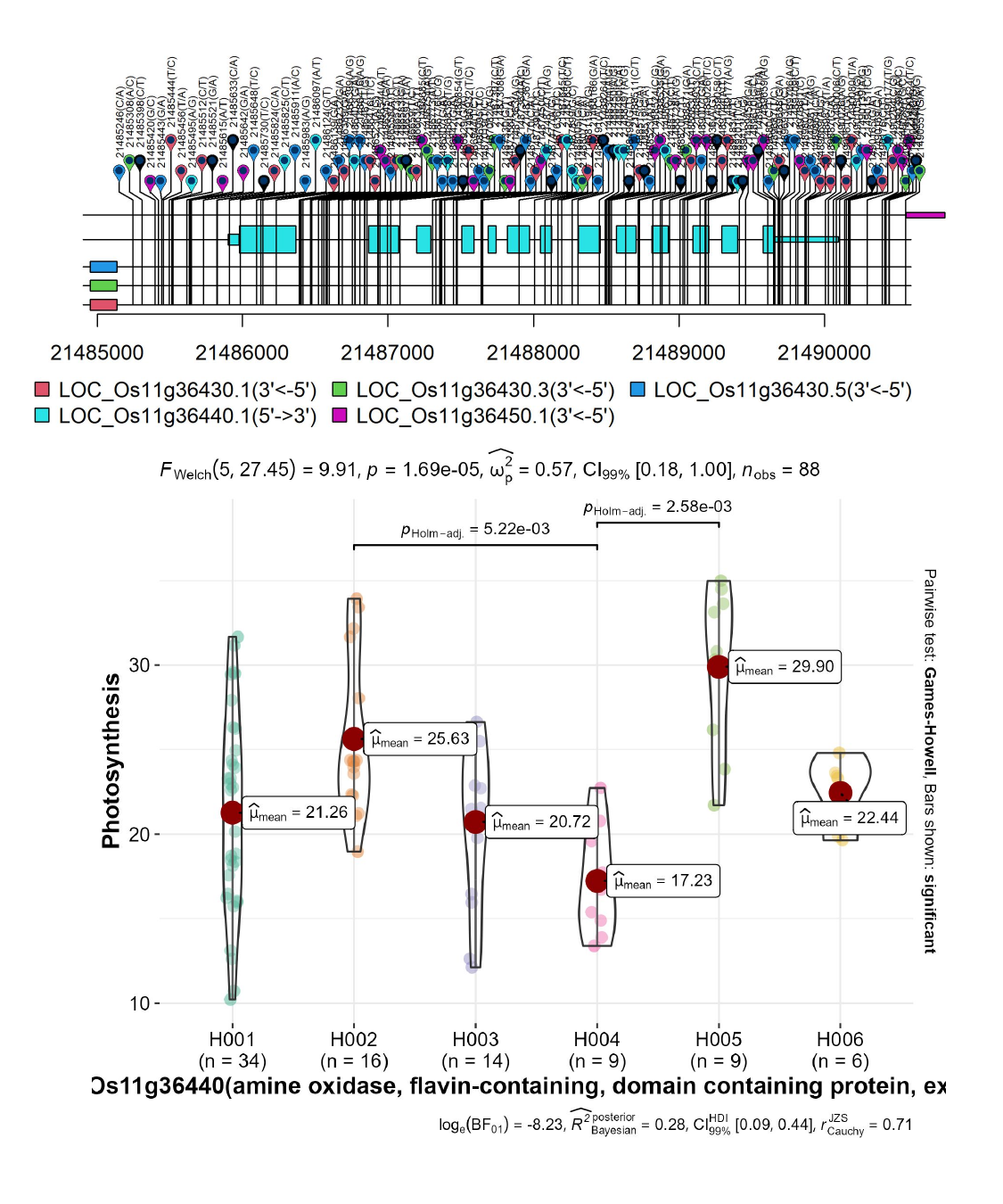

## Slide 44
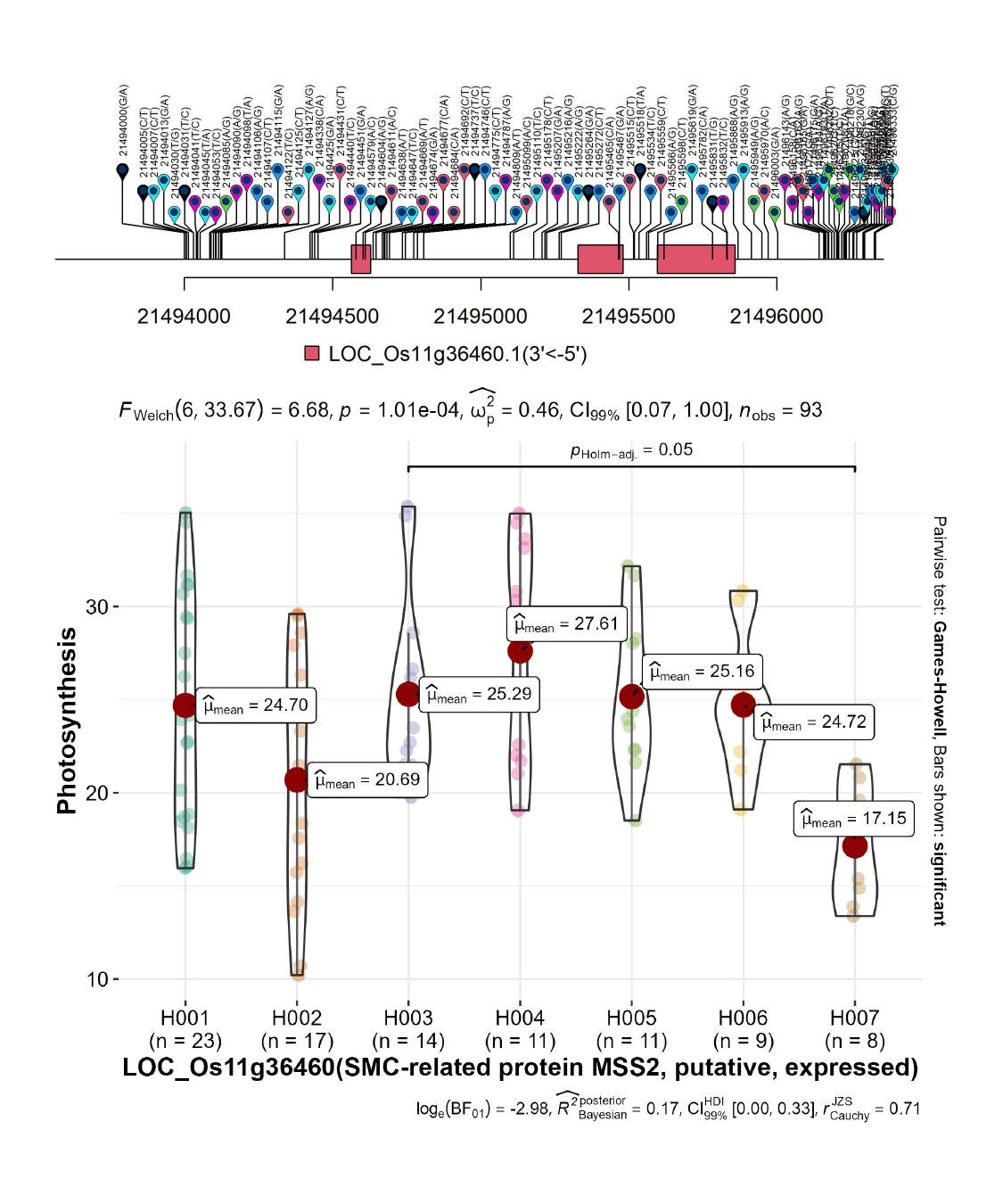

Supplement: Supplementary file 5 [file Presentation4.pptx]
